# Investigation of ancestral alleles in the Bovinae subfamily

**DOI:** 10.1186/s12864-021-07412-9

**Published:** 2021-02-08

**Authors:** Maulana M. Naji, Yuri T. Utsunomiya, Johann Sölkner, Benjamin D. Rosen, Gábor Mészáros

**Affiliations:** 1 University of Natural Resources and Life Sciences (BOKU), Vienna, Austria; 2 São Paulo State University (Unesp), School of Veterinary Medicine, Department of Production and Animal Health, Araçatuba, São Paulo Brazil; 3 International Atomic Energy Agency (IAEA) Collaborating Centre on Animal Genomics and Bioinformatics, Araçatuba, São Paulo Brazil; 4 AgroPartners Consulting. R. Floriano Peixoto, 120-Sala 43A-Centro, Araçatuba, SP 16010-220 Brazil; 5 Personal-PEC. R. Sebastiao Lima, 1336-Centro, Campo Grande, MS 79004-600 Brazil; 6 Agricultural Research Service USDA, Beltsville, MD USA

**Keywords:** Ancestral allele, Bovinae, Gene ontology, Whole genome sequences

## Abstract

**Background:**

In evolutionary theory, divergence and speciation can arise from long periods of reproductive isolation, genetic mutation, selection and environmental adaptation. After divergence, alleles can either persist in their initial state (ancestral allele - AA), co-exist or be replaced by a mutated state (derived alleles -DA). In this study, we aligned whole genome sequences of individuals from the Bovinae subfamily to the cattle reference genome (ARS.UCD-1.2) for defining ancestral alleles necessary for selection signatures study.

**Results:**

Accommodating independent divergent of each lineage from the initial ancestral state, AA were defined based on fixed alleles on at least two groups of yak, bison and gayal-gaur-banteng resulting in ~ 32.4 million variants. Using non-overlapping scanning windows of 10 Kb, we counted the AA observed within taurine and zebu cattle. We focused on the extreme points, regions with top 0. 1% (high count) and regions without any occurrence of AA (null count). High count regions preserved gene functions from ancestral states that are still beneficial in the current condition, while null counts regions were linked to mutated ones. For both cattle, high count regions were associated with basal lipid metabolism, essential for survival of various environmental pressures. Mutated regions were associated to productive traits in taurine, i.e. higher metabolism, cell development and behaviors and in immune response domain for zebu.

**Conclusions:**

Our findings suggest that retaining and losing AA in some regions are varied and made it species-specific with possibility of overlapping as it depends on the selective pressure they had to experience.

**Supplementary Information:**

The online version contains supplementary material available at 10.1186/s12864-021-07412-9.

## Background

Divergence and speciation result from long periods of adaptation, selection, and genetic drift after separation of subpopulations. Separation forces individuals to adapt within the current isolated environment and gradually differ from the initial population. Various methodologies and theories have been proposed in efforts for deciphering this process since nineteenth century [1].

Recently, the availability of whole genome sequences (WGS) has become of increasing importance in genetic studies [2]. In cattle studies for example, WGS data of various breeds have been used for inference of demographic history, identification of production traits, calculation of effective population size, estimation of genetic relationships, and population structure analysis [3–5].

In evolutionary analysis, synteny blocks can be inferred as conserved relationships of genomic regions in different species anchored by sets of orthologues genes. With varying size, these blocks can be co-localized in different karyotypes of modern species’ respective genomes. Moreover, synteny blocks can be clustered into lineage-specific ones, such as to primates, Rodentia, Felidae, Camelidae, Chiroptera and Bovidae as suggested in a study of syntenic analysis using 87 mammalian genomes [6]. However, orthologous genes within these lineage-specific synteny blocks may present allele variations due to independent evolutionary event after the speciation [7].

Alleles having diverged through mutation are called derived alleles (DA), while alleles that persist in their initial state are termed ancestral alleles (AA) [8]. A reasonable method to assess AA is by comparing shared polymorphic sites of closely related species. Alleles that are still intact and shared by all the related species are most likely the ancestral allele [9]. Another method consists of verifying the allelic state of the last common ancestor (LCA) or the allele within current populations that least differs from the LCA [10].

In a study of autosomal single nucleotide polymorphisms (SNP) in pig, ancestral and derived allelic states of SNP were inferred using four Sus species (*Sus celebensis, Sus barbatus, Sus cebifrons,* and *Sus verrucosus*) and one outgroup species of African warthog for focal species of *Sus scrofa* [11]. In human studies, the out-group species for inferring AA are primates, namely orangutan (*Pongo sp.*), macaques (*Macaca sp.*), gorilla (*Gorilla sp.*), and bonobos (*Pan paniscus*) [12]. In a cattle study of Utsunomiya et al. (2013) using HD-SNP, Gaur (*Bos gaurus*), water buffalo (*Bubalus bubalis*) and Yak (*Bos grunniens*) were utilized as focal species for cattle.

Defining the ancestral and derived states at polymorphic nucleotide sites is required to test proposed hypotheses regarding molecular evolution processes, such as estimation of allele ages, formation of linkage disequilibrium (LD) patterns and genomic signatures as a result of selection pressures [13, 14]. Human WGS studies benefit from AA database for population analysis, but such a database is lacking in cattle. Consequently, each study repeatedly generates its own putative AA list [5, 12, 15].

Therefore, the goal of this study is to fill this gap and to determine a fixed set of AA in cattle by using outgroup species in the *Bovinae* subfamily, namely gaur, yak, bison, wisent, banteng, and gayal sequences. In addition, we scanned the list of AA for physical regions linked to conserved and mutated traits in taurine and zebu cattle.

## Results

### Read alignments and principal component analysis

**Fig. 1:**
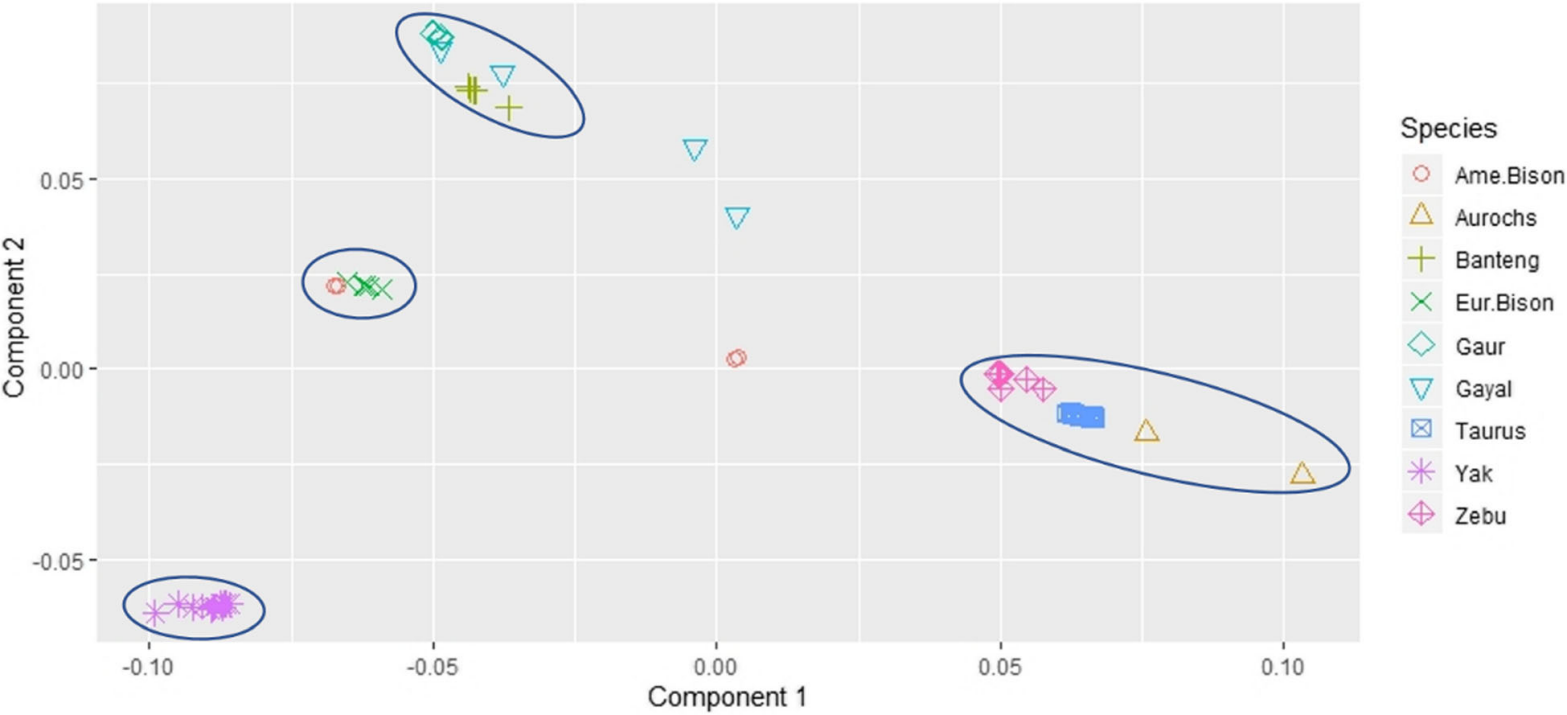
Principle component analysis

We evaluated alignment results of different species within the Bovinae subfamily against the latest cattle reference sequence ARS-UCD1.2 [16]. On average, the genome was covered by ~5x for banteng, taurine cattle, European bison, gayal, and yak, ~4x for American bison and zebu cattle, and ~ 3x for aurochs. Principle component analysis (PCA) formed clusters and separation of individuals among these nine groups (Fig. 1). Four principal components (PC) explained 36.7, 24.9, 20.5, and 17.7% of the variance for first, second, third, and fourth PC, respectively. Projected by the PC1 and PC2, these Bovinae individuals are clustered together with its closest relatives evidencing genetic relatedness within its sub-species. PC1 explains divergence of cattle (aurochs, zebu, and taurine), from the rest. PC2 gives divergence between cluster containing gayal-gaur-banteng (gagaba) from clusters containing yak and bison. Thus, we can group these individuals into four, namely cattle-aurochs cluster, gagaba cluster, bison cluster, and yak cluster. Outlier individuals, i.e. two gayals and the American bison, may indicate individuals carrying introgression from cattle.

### Phylogenetic trees

**Fig. 2:**
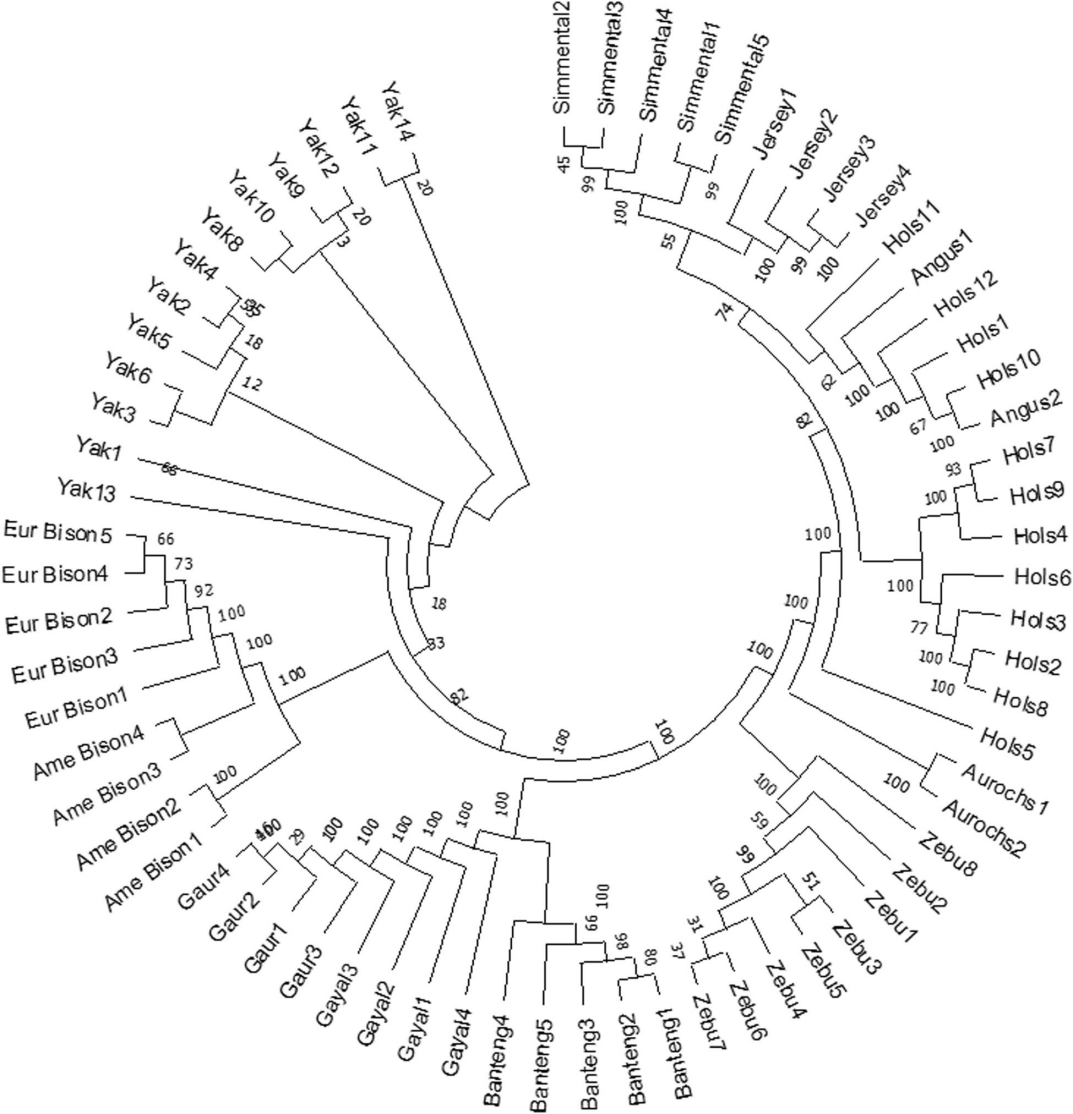
Phylogenetic tree based on chromosome 1

Maximum Likelihood phylogenetic trees were constructed for each chromosome [see Additional file 1]. Inferred trees were all similar with Fig. 2 below displaying the tree from chromosome one. In concordance with the principal component analysis, 13 yak individuals are situated together in the top clade of the tree. European bison and American bison have the same node of ancestor, with American bison perceived to be more ancestral. This is in line with a previous study where sister relationships were indicated between American bison and European bison and also between bison clade and yak [17]. Banteng-gaur-gayal share a clade together, however, variations in the order within these three species exist in trees inferred from different chromosomes [see Additional file 1]. Zebu cattle reside on the same upper node with the taurine cattle group. Each breed of taurine cattle is well clustered together except for several Holstein individuals. Based on all trees, we defined yak as the most distant relative as it is positioned on the furthest node from cattle.

### Inferring ancestral allelic states

**Fig. 3:**
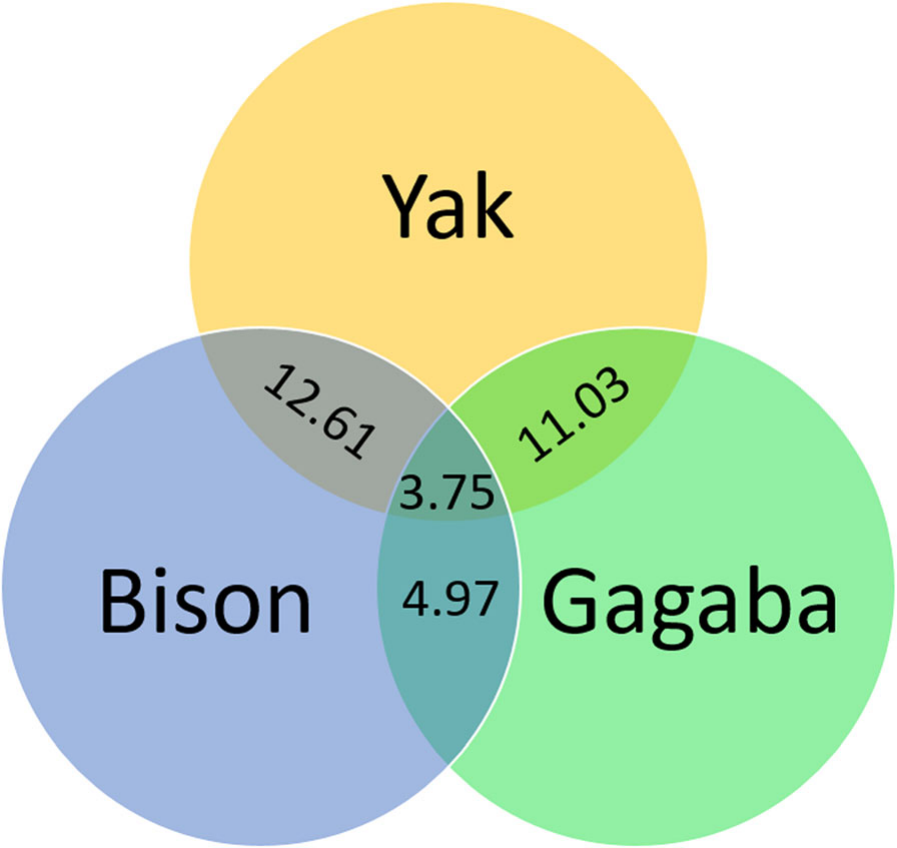
Intersection of defined ancestral alleles (in millions) from three lineages; bison, yak, and gayal-gaur-banteng (Gagaba)

The main output of this paper is a list of defined ancestral alleles for cattle, available at https://tinyurl.com/cattle-aa . This list is necessary for several tools used for studying selection signature such as iSAFE, iHS, xp-EHH, EHHST, and hapFLK [18–23] which were built for human population genetics study. We provide this dataset as a foundation for future comparisons of selection signatures in various cattle breeds. It is stored in a simple format of .txt and comprised of 6 columns of chromosome, position, number of alleles, defined ancestral allele, frequency, and which groups agree on the defined ancestral allele. AA were determined as alleles that are fixed in two of three outgroup lineages. Using allele frequency over all individuals in outgroup, we defined ~ 32.4 million variants that are fixed across 29 chromosomes as AA corresponding to 1.2% of the total genome. As shown in Figs. 3, 3.75 million alleles were defined as ancestral from all three lineages of bison, yak, and gayal-gaur-banteng (gagaba). GC content percentage of ancestral alleles is 58%, which is higher than the GC content of the reference genome (~ 42%). Yet, it is worth noting that 22% of these AA are within active transcript regions.

### Windows with high ancestral allele counts in taurine and zebu cattle

**Fig. 4:**
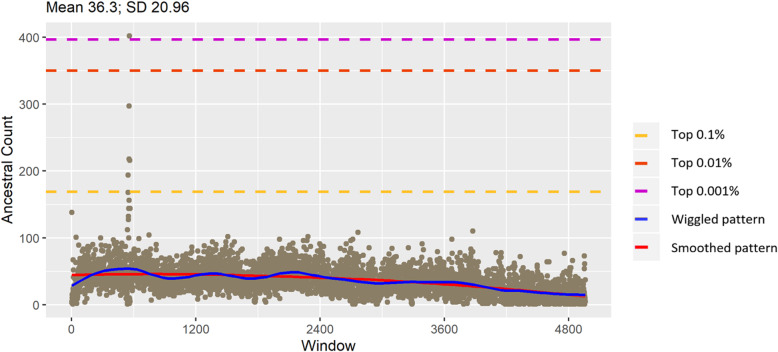
Distribution of ancestral count in taurine chr 29

**Fig. 5:**
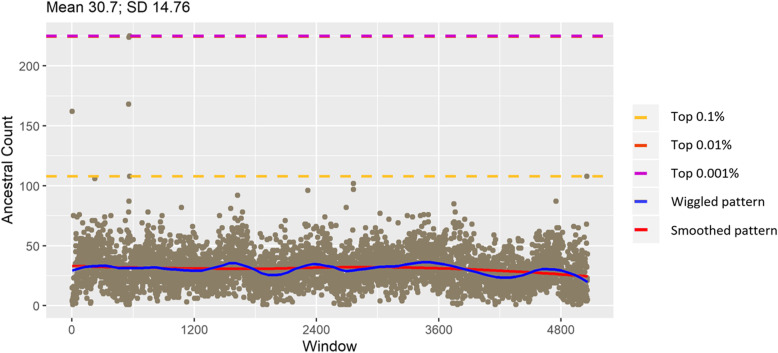
Distribution of ancestral count in zebu chr 29

We counted AA by non-overlapping windows of 10 Kb in taurine and zebu cattle separately. Figures 4 and 5 present the distribution of AA on chromosome 27 for taurine and zebu, respectively (The distribution of AA for all chromosomes can be found in Additional file 2). For taurine cattle, ancestral allele counts arguably tend to decrease towards the end of chromosome, as demonstrated by the fitted red trend lines. In zebu cattle, ancestral counts are relatively flat throughout the chromosome. Yet, the amplitude pattern is stable for taurine, but more variable for zebu cattle (blue trend line). Peaks of high ancestral alleles count regions in contrast with background averages number of ancestral alleles are clearly distinguished in chromosome 1, 4, 5, 7, 10, 12, 13, 14, 15, 18, 27, 29 in taurine cattle and 1, 2, 3, 4, 6, 10, 12, 13, 14, 15, 18, 23, 27 in zebu cattle [see Additional file 2].

**Fig. 6:**
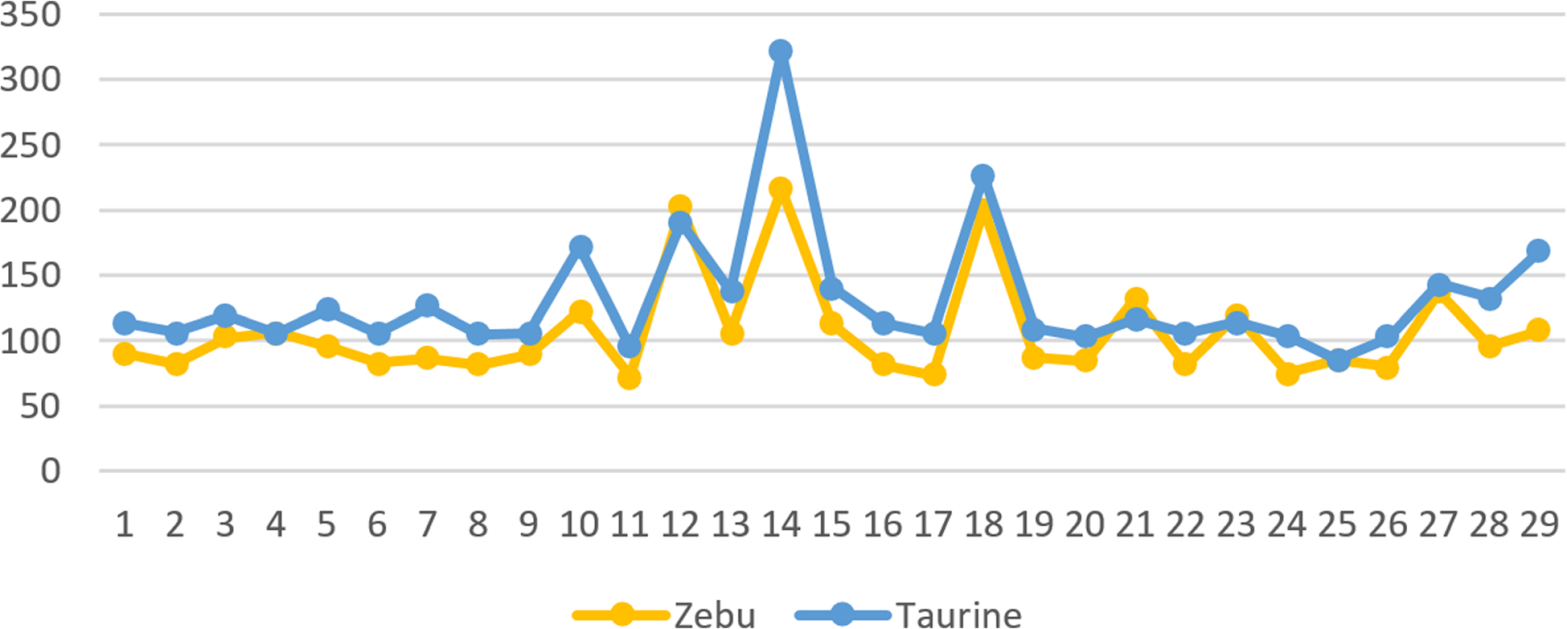
Threshold of 0.1% top ancestral count

Ancestral counts for the top 0.1% are beyond the mean plus three standard deviations. For taurine cattle, the lowest chromosome specific threshold for ancestral count was 122 on chromosome 25 while the highest was 302 on chromosome 14, while for zebu cattle, it was 102 in chromosome 1 while the highest 200 on chromosome 12. The trends for both groups were similar as shown in Fig. 6. Taurine cattle has mostly higher thresholds implying there are more windows with higher counts of AA compared to zebu cattle.

### Windows without the occurrence of ancestral alleles

**Fig. 7:**
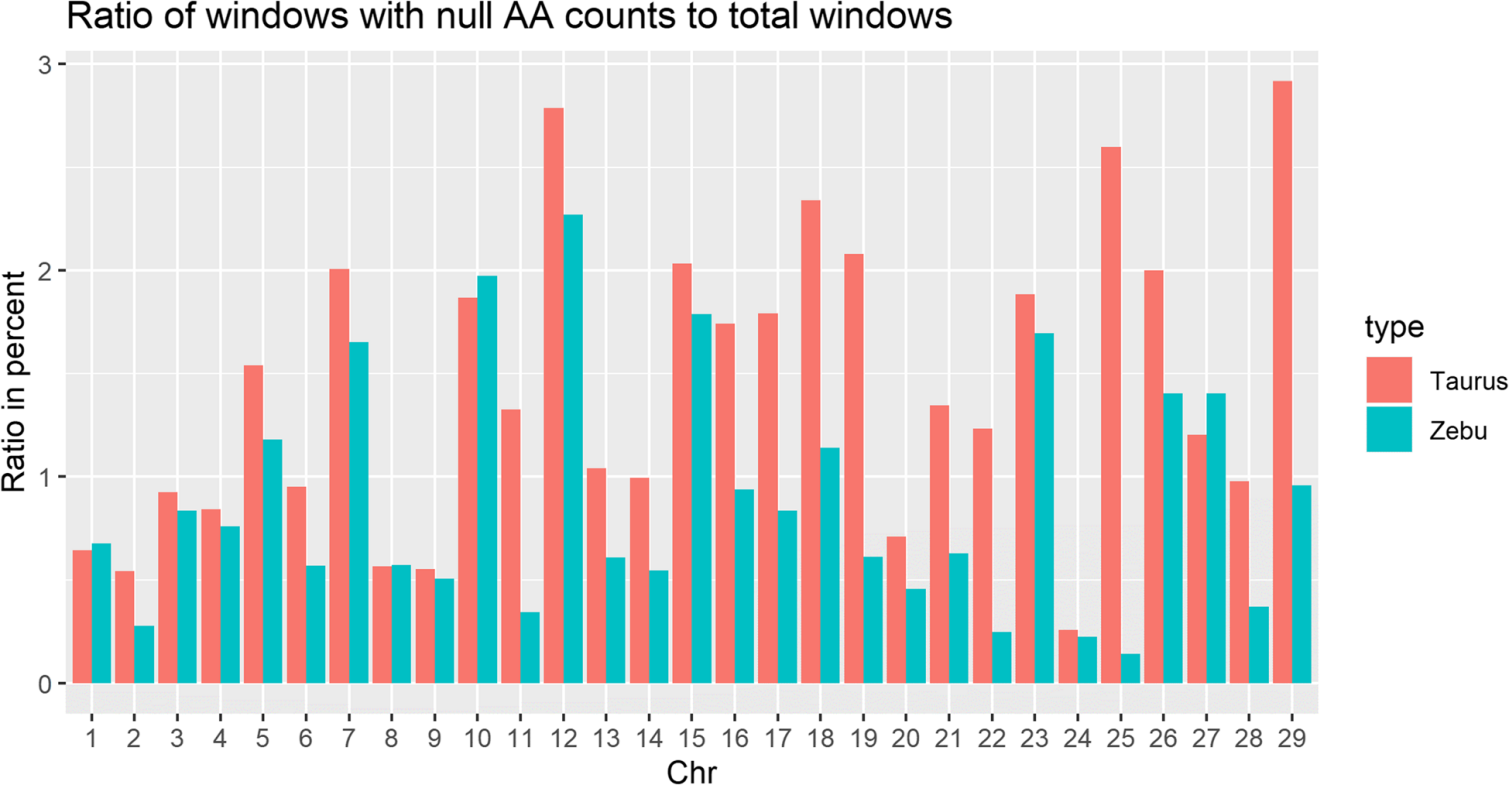
Ratio of windows with null AA counts to total windows

We found 3306 windows without AA in taurine and 2189 windows in zebu. The highest ratio of windows with null AA counts to total windows was 2.9% on chromosome 29 in taurine and the lowest is 0.14% in chromosome 25 of zebu cattle (Fig. 7). Overall, taurine has more windows without AA except for chromosome 1, 8, 10, and 27. Windows without AA could be explained by a lack of defined AA from outgroups, meaning, there were no fixed alleles that can be found in at least two lineages. Another reason could be that derived alleles are now the major alleles on polymorphic sites, therefore we could not find AA within these windows. In taurine cattle, 65% of windows without AA are due to the latter reason, while in zebu it is 46%.

### Annotation of scanning windows with high number of ancestral alleles

We annotated each scanning window passing the respective threshold of top 0.1%, corresponding to 255 regions in taurine and 258 regions in zebu across 29 chromosomes. These regions contained 20 genes in taurine and 40 genes in Zebu. Both groups retained genes functioning in arachidonic acid secretion (GO:0050482), phospholipid metabolic process (GO:0006644), and lipid catabolic process (GO:0016042) indicated by LOC100125947 and PLAG2A, as shown in Table 1. These three terms are mainly functioning in primary metabolic process of lipid. Function of defense response to bacterium (GO:0042742) was exclusive to taurine. DEFB genes family in GO:004742 were secreted by leukocytes and epithelial tissues. It is known for its function similar to antimicrobial defense by penetration to microbial’s cell membrane and cause microbial death [24]. While calcium ion imports (GO:0070509), represented by SLC8A1 and CACNA1D, was exclusive to zebu defined as function of maintaining and transporting cellular entity in a specific location.

**Table 1:** GO terms of genes indicated by high count ancestral alleles

GOTerm	Function	Count	PValue	Genes	Fold Enrichment	Bonferroni
Taurine						
GO:0050482	Arachidonic acid secretion	3	5.0E-04	LOC100125947, PLA2G2A	84.12	0.02
GO:0006644	Phospholipid metabolic process	3	7.7E-04	LOC100125947, PLA2G2A	67.76	0.03
GO:0016042	Lipid catabolic process	3	2.9E-03	LOC100125947, PLA2G2A	34.85	0.10
GO:0042742	Defense response to bacterium	2	9.0E-02	DEFB7, DEFB3	20.08	0.97
Zebu						
GO:0050482	Arachidonic acid secretion	3	8.7E-04	LOC100125947, PLA2G2A	65.00	0.06
GO:0006644	Phospholipid metabolic process	3	1.3E-03	LOC100125947, PLA2G2A	52.36	0.10
GO:0016042	Lipid catabolic process	3	5.0E-03	LOC100125947, PLA2G2A	26.93	0.32
GO:0070509	Calcium ion import	2	2.4E-02	SLC8A1, CACNA1D	78.55	0.85

### Annotation of scanning windows without ancestral alleles

There were 713 windows in taurine with protein coding genes, while in zebu 121 windows were found. GO terms of regions within scanning windows without AA are attached [see Additional file 3]. There are 42 GO terms defined for taurine and 7 GO terms for zebu. Among those, three terms were found in both, i.e. two antigen processing terms (GO: GO:0002474 and GO:0019882) and negative regulation of endopeptidase activity (GO:0010951).

In taurine cattle, apart from terms related to immune system process and cellular function, there are GO terms exclusive to taurine cattle that are related to production traits. For example, GO:0008654, GO:0043410, GO:0045725, GO:0060048, GO:0008016, are related to metabolic process of phospholipid, protein, glycogen, and regulation of muscle and heart contraction. GO:0007613 and GO:0035176 are related to mental information processing systems and is part of learning or memory abilities which can affect cognition and behavior as indicated by CRTC1, TH, ITPR3, DBH, SORCS3 genes. ITPR3 is known as well for process of sensory perception of taste. CRTC1 gene in human has highest transcript expression in brain compared to other tissues and is known for affecting eating behavior [25].

GO:0009611, GO:0071364, GO:0071560 and GO:0008286 are related to response of stimulus such as stress from wounding and transforming growth factor. GO:0048469, GO:0010976, GO:0060425, GO:0002062, are terms related to development of cell, neuron, lung morphogenesis and chondrocyte differentiation in cartilage outgrowth as part of skeletal system and animal organ development as pointed by PTH1R, COL2A1, COL11A2, WNT7A, RUNX3, SOX10, GATA2, PTH1R, and SOX18 genes.

**Fig. 8:**
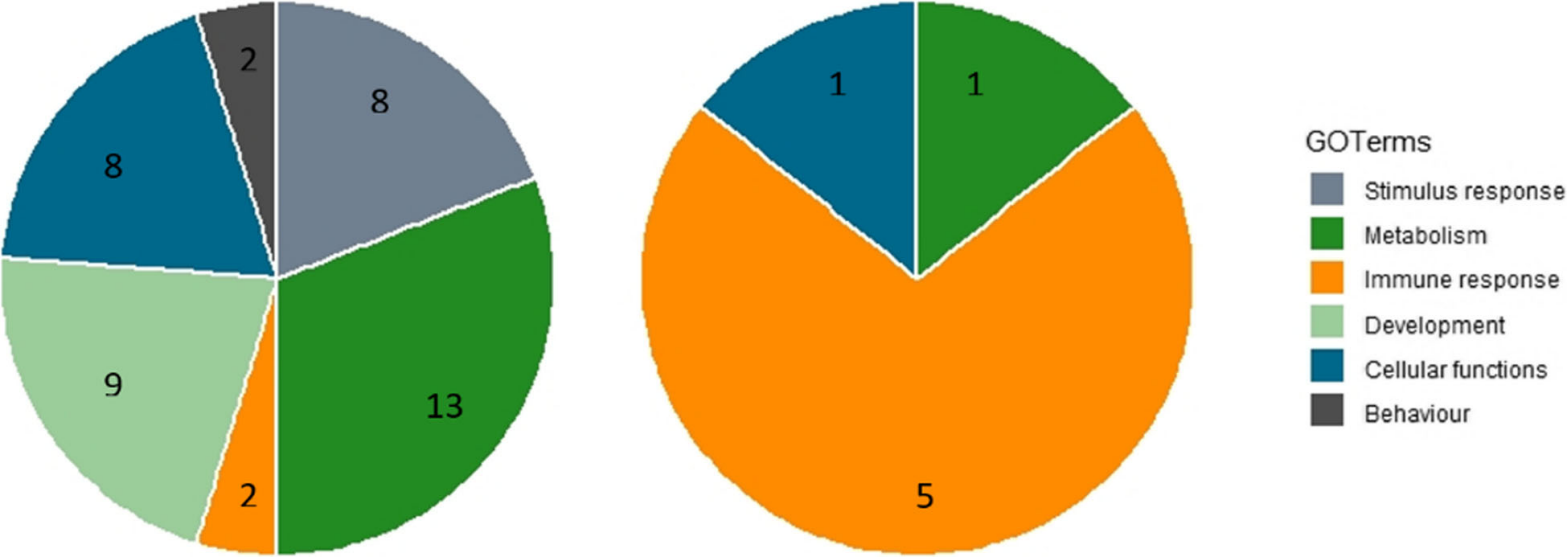
GO terms for regions without ancestral alleles (taurine-left; zebu-right)

Regions without AA in zebu were mainly related to 5 GO terms in domain of immune response and one term related to cellular process of transmembrane transport. Figure 8 represented distribution of terms found in regions without AA. It is dominated by metabolism terms in taurine and immune response in zebu.

## Discussion

We forced mapping short read sequences of different species within Bovinae subfamily into the latest cattle RefSeq ARS-UCD1.2 irrespective of their actual genome structure. Phylogenetic trees were built based on the SNP variants in autosomes. We used subsets of all variants per chromosome to comply with maximum 50,000 markers/sequences per output of the analysis as directed by the software [26]. Despite an unequal number of individuals representing each group, we could infer relationships based on variant similarity and defined four lineages of yak, bison, gagaba and cattle. Even though still related, none of outgroups were in ancestor-descendant relationships apparently.

Defining AA by only a single lineage was not an option since any of the current lineages could have undergone independent evolutionary events and might have diverged from the initial ancestral state. Alleles were set to be ancestral strictly if they are fixed and shared by at least two lineages of yak, bison and gagaba, complying with other similar studies [9, 15]. Using the same dataset, we infered the ancestral alleles several times resulting in the same list of alleles as we strictly considered only variants with fixed allele (100% frequency) in each species. Although, we used the best dataset available in terms size, sequence read quality, and coverage for the outgroup species, additional re-sequencing data of the outgroup species might have slightly modified the defined ancestral alleles as the frequency for those fixed alleles might be changed by new individuals. However, as a rigid solution, we defined fixed alleles as ancestral only if they are fixed and shared by at least two different lineages.

Scanning windows of 10Kb were chosen after a preliminary comparison between 1Kb, 10Kb, and 50 Kb windows and considering the average gap between high density markers of 4Kb in identifying different types of selection in a previous study [27]. Ancestral allele counts within scanning windows in taurine and zebu cattle varied in the genome. We took two extreme ends of the occurrence distribution; one is windows with the top 0.1% highest count and second is windows without ancestral allele count. Based on the knowledge that mutation occurs across autosomes with different rates on different scales [28], we expected ancestral allele frequency to be changing as the mutations emerge. Thus, we assumed windows with highest count of AA are the conserved ones while windows without AA are the ones containing relevant mutations, considering important traits or genes that were retained along evolutionary process [7, 8].

Regions with high ancestral counts have GO terms related to primary metabolic process of lipid in both cattle. Genes within these GO terms are likely retained in ancestral states because their basic function are still beneficial. Despite different environments, both cattle need to store energy efficiently in form of lipids. Although cattle diet usually contains two to 4 % lipid, it contributes up to 50% of fat in milk and the most concentrated source of energy. In contrast to human, where liver is the primary site, fatty acid synthesis occurs at adipose tissue in ruminants [29, 30]. Adipose tissue acts as reservoir for efficient energy storage in allowing cattle and mammals in general for surviving adversities such as food shortages during severe winter for taurine or drought for zebu [31]. Defense response to bacteria (GO:0042742) was detected from regions with high ancestral counts in taurine, but found in regions without AA in zebu. In taurine high count regions, DEFB7 and DEFB3 are within this term, while regions without AA in zebu are DEFB6, LOC781146, DEFB1, DEFB3.

For regions without AA where expected mutation occurs, GO terms may have correlated and not necessarily independent from each other as pointed by its function. For grouping, we used the prevalent terms within ancestor charts in quickGO. In taurine, terms are related to behavior, cellular functions, tissue development, immune system, metabolism, and stimulus response. These are in line with suggestion from previous study for likelihood of genes function without AA and positive selection [32]. Within this scope, more GO terms found in taurine cattle compared to zebu possibly due to more intensive selection for production traits. Aiming for higher growth rate, carcass quality, feed efficiency, calving interval, milk production and body conformity has directed animals to be more efficient with higher metabolism rates [33–35]. These selection events might not only be affecting a narrow-region of genome. Instead, it altered several regions simultaneously as production traits are complex involving many QTLs or regions across chromosome with small contribution by each for the expression [36, 37].

In zebu, mutated regions were mainly linked to GO terms of immune response and little to cellular functions and metabolism. Concordance to suggested previously where zebu has been bred to adapt with more marginal production environments compared to taurine [38, 39]. Evidences showed different in relative importance on innate and adaptive immune response towards cattle tick *Rhipicephalus microplus* infestation between zebu and taurine. Skin inflammatory response by high secretion of granulocytes and T-lymphocytes in taurine is not necessary could cease tick invasion. But, an earlier inflammatory response and secretion of an alternate non-volatile T-cell in zebu were more efficient in repel this tick invasion [40, 41].

Nevertheless, not all genes within previously mentioned GO terms can be linked directly to positive selection. As mentioned in previous study, BOLA gene families, which we found also in regions without AA, are a result of balancing selection aiming for preserving genetic diversity as heterozygous animals have more advantage than the homozygous ones [27]. Similarly, we cannot confirm whether genes here are main targets of selection or as hitchhiking effect from genes of interests. For example, genes within GO:0007613, related to behavior memory and taste preferences, could be intended for selection because breeder preferences of tame, good mothering ability and non-picky animals in terms of feed and housing. Alternatively, it could be indirectly selected because animals have to cope with commercial environment as suggested that behavioral patterns were altered for animals in pasture and confinement cases [42, 43].

Our findings suggest that retaining and losing AA in some genes or regions are varied and made it species-specific with possibility of overlapping as it depends on the selective pressure they had to experience. Future work in finding overlapped domains detected by different tools for selection signatures would confirm specific regions/functions peculiar for each various cattle breeds.

## Conclusions

We inferred ancestral alleles by combining fixed alleles in three lineages of cattle outgroups. Regions conserving more primitive functions indicated by high count ancestral alleles were linked to lipid metabolism in taurine and zebu. Meanwhile, regions undergone mutation indicated by no preserved ancestral alleles were found more on taurine than zebu. These regions were linked to production traits in taurine and robustness traits in zebu.

## Methods

### Dataset

**Fig. 9:**
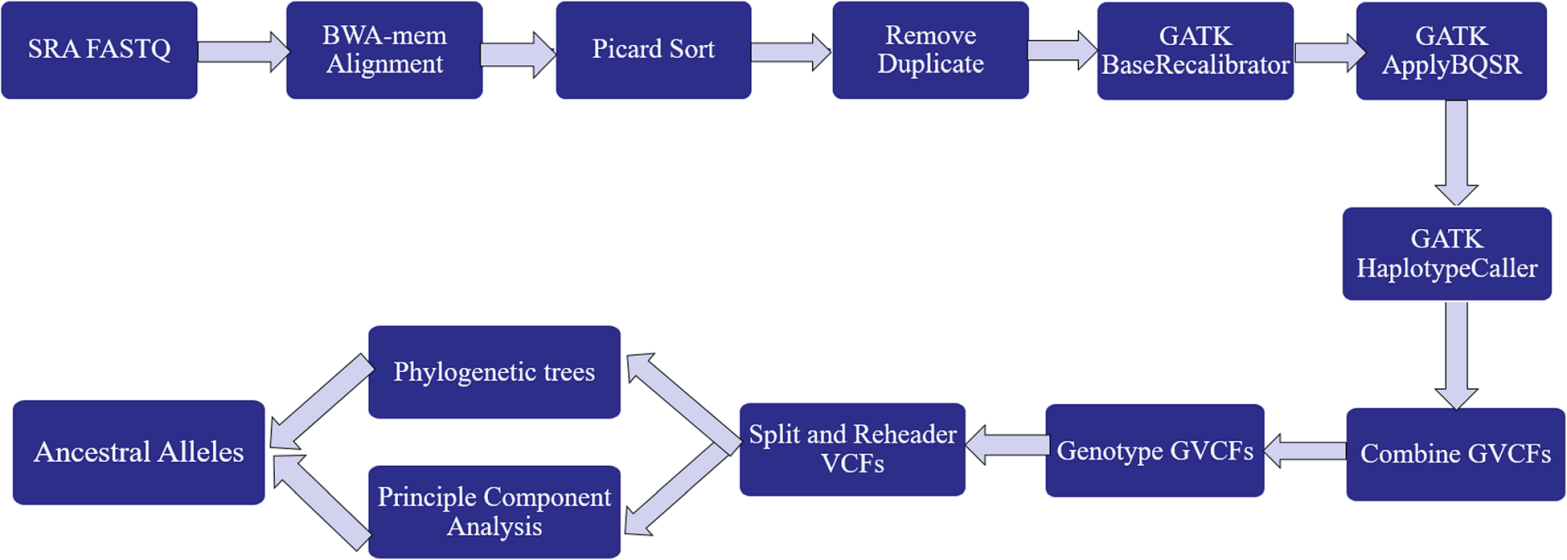
Workflow of the ancestral allele analysis

WGS of different (sub)species were obtained from NCBI BioProject in fastq format as listed in Table 2, please refer to ‘Availability of Data and Materials’ section for the accession numbers. Taurine cattle group was represented by several commercial breeds, i.e. Holstein, Angus, Jersey, and Simmental. Workflow of the ancestral analysis pipeline is shown in Fig. 9.

**Table 2:** List of whole genome sequences data

Name	Species	N	Avg. Mbases	Avg. read length	Mapped reads (%)	Clean reads^a^ (%)	Coverage	BioProject	Reference
Taurine cattle	*Bos taurus*	23	18,913	248	98.37	78.61	5x	PRJNA238491, PRJNA277147	[44, 45]
Banteng	*Bos javanicus*	5	16,596	250	98.36	84.30	5x	PRJNA427536	[46]
Gaur	*Bos gaurus*	4	18,428	300	98.50	71.61	5x	PRJNA427536	[46]
Yak	*Bos grunniens*	13	22,177	201	98.51	79.47	4x	PRJNA285834	[47]
American Bison	*Bison bison*	4	13,364	200	98.51	82.81	4x	PRJNA427536	[46]
European Bison	*Bison bonasus*	5	18,113	250	98.51	89.67	5x	PRJNA427536	[46]
Gayal	*Bos frontalis*	4	18,610	250	98.49	86.71	5x	PRJNA427536	[46]
Aurochs	*Bos taurus primigenius*	2	17,105	62	98.51	45.17	3x	PRJNA294709	[32]
Zebu cattle	*Bos taurus indicus*	8	10,863	428	98.60	76.98	4x	PRJNA507259, PRJNA427256	[48]

^a^Reads remaining after base quality score recalibration process and used for calling variants

### Alignment and variant calling

Following Best Practice procedure by Genome Analysis Tool Kit [49–51], single interleaved data sets of FASTQ from each individual were not trimmed based on phred score, because GATK tool takes care of these low quality reads on later step during recalibration process. Datasets were mapped against the cattle reference sequence ARS.UCD-1.2 [16] using BWA-MEM [52] with default parameters. The raw mapped reads were sorted by chromosome position using SortSAM function. Sorted BAM files then underwent duplicates marking using Picard MarkDuplicates. Base Quality Score Recalibration (BQSR) was carried out to adjust the base scores towards various possibly systematic errors. BQSR required supporting files, such as known variant sites in vcf format [44], index and dict files of reference sequence created by using Samtools [53]. Report file in table form was needed for the next step of ApplyBQSR with an output of analysis ready BAM files. Analysis ready BAM files were individually called for variants using HaplotypeCaller with GVCF mode for preparation in cohort analysis workflow. Individual VCFs then combined using CombineGVCFs and went through joint-call cohort for GenotypeGVCFs. SplitVCFs tool was used to split SNPs and Indel variants from cohort VCF file. SNP variants were filtered out for parameter of mapping quality less than 40, QUAL less than 30 and quality by depth less than 30. Header editing of vcf files and splitting by each chromosome were done using bcftools and vcftools.

### Principal component analysis

Multisample VCF file was converted to binary plink format using VCFtools. The indep algorithm in PLINK [54] was used with default parameters of 50 variants window size units shifting for every 5 variants with pairwise r^2^ threshold of 0.7. This step selected a set of independent variants for reducing redundancy. Then, we set four components to reduce dimension of the whole independent variants and plotted the species based on the first two components.

### Phylogenetic trees

We constructed phylogenetic trees from autosomes of our species similar to other studies, so called phylogenomes [55, 56]. SNPhylo [26] processed original multisample VCF files of chromosome 1 to 29 separately to reduce redundant variants based on LD. Parameters were set to 0.1 Low Coverage Samples (PCLS), depth coverage of two, 0.9 LD threshold, 0.1 minor allele frequency and 0.1 missing rate. These parameters were set to meet the maximum variants output by the program and roughly reduce the variants to 10% in output fasta. MEGA X built initial tree using Maximum Parsimony method and inferred final phylogenetic trees for each chromosome by using Maximum Likelihood method and Jukes-Cantor model with 200 bootstraps [57, 58].

### Inferring ancestral allelic states

VCFtool was used to call allele frequency spectrum from un-prunned VCF files. Considering branches in phylogenetic trees and clusters of PCA, we defined three lineages of cattle outgroup, i.e. Yak, Bison (American bison and European bison), and Gagaba (Gayal-Gaur-Banteng). For each site, frequency of two alleles of A and a represented by p(A) and q(a) frequency. If p(A) frequency of 1 and found in at least two lineages, we defined “A” allele as ancestral for that site.

We used R [59] to create list of these defined AA for all autosomes. Following packages in R were used to support data analysis and visualization: dplyr [60], ggplot2 [61], and stringr [62]. R functions for calling the ancestral allele in this study are provided in https://github.com/mas-agis/ances-al with an example run for all the scripts provided in [63].

### Comparison to cattle groups

A custom script was used to compute summary statistics of allele frequencies and to compare which AA are still intact in zebu and taurine cattle. Notation 1 below, defining how we calculated *ϑ*, the changing frequency of ancestral allele compared to cattle group:

$$ \left(\mathrm{Notation}\ 1\right):\vartheta =x-p\left({A}_{AA}\right), $$

where x is the frequency of same allele A in cattle as the ancestral *p*(*A*_*AA*_).

Given ancestral allele denotes as *p*(*A*_*AA*_) with frequency of 1 for A allele, *ϑ* is calculated by subtract *p*(*A*_*AA*_) from *x*. Where *x* can be both major *p*(*A*_*cattle*_) or minor *q*(*A*_*cattle*_) allele in cattle with condition that *x* must represent the same allele A as the ancestral one. We assigned *ϑ* for each site of SNP data across the autosome. For example, if major allele in cattle is A matching to *p*(*A*_*AA*_), thus

$$ \vartheta ={p}_{cattle}-p\left({A}_{AA}\right)=100\%-100\%=0 $$

while if minor allele A in cattle matching *p*(*A*_*AA*_), then

$$ \vartheta ={q}_{cattle}-p\left({A}_{AA}\right)=30\%-100\%=-0.7 $$

We filtered *ϑ* with value of 0 meaning ancestral allele persist in cattle groups. To count how many sites persisting with AA, we assigned f(*ϑ*) score is 1 for every *ϑ* equal to zero, otherwise we assigned zero to the f(*ϑ*) as notation 2 below. We used non-overlapping windows of 10 Kb to sum up sites that have value of 1. By this scanning windows, autosomes were divided into regions and total counts were reported. We selected two extreme conditions of windows with highest count and null count of AA. Indicated regions from both conditions were used for further analysis.

(Notation 2):

$$ \boldsymbol{T}\left(\boldsymbol{\vartheta} \right)=\sum \limits_{\boldsymbol{t}=\mathbf{1}}^{\boldsymbol{n}}\ \sum \limits_{\boldsymbol{i}=\mathbf{1}\mathbf{0}\mathbf{0}\mathbf{0}\mathbf{0}\left(\boldsymbol{t}-\mathbf{1}\right)}^{\mathbf{10000}\boldsymbol{t}}\boldsymbol{f}\left({\boldsymbol{\vartheta}}_{\boldsymbol{i}}\right),\boldsymbol{where}\ \boldsymbol{f}\left(\boldsymbol{\vartheta} \right)=\left\{\begin{array}{c}\mathbf{1},\boldsymbol{\vartheta} =\mathbf{0}\\ {}\mathbf{0},\boldsymbol{\vartheta} \boldsymbol{\ne}\mathbf{0}\end{array}\right. $$

### Annotation region of interest

Physical regions indicated by previous step were taken as input for ANNOVAR [64]. We then excluded regions that are fall in the intergenic, downstream and upstream of known genes, leaving only regions that overlapping with functional genes. We filtered out genes defined by highest count regions if were found also in regions without ancestral counts. We used this list of genes for GO analysis using DAVID 6.8 [65, 66]. We report GO of biological process with the Bonferroni corrected *P*-values. Definition and supporting information related to GO were retrieved from database of European Bioinformatics Institute [67].

## Supplementary Information

**Additional file 1.** Phylogenetic trees from each chromosome

**Additional file 2.** Distribution of ancestral allele in all chromosomes of taurine and zebu

**Additional file 3.** Annotation of regions without ancestral alleles

**Additional file 4.** Accession numbers of individual sequencing reads

## Data Availability

Accession numbers for each individual used in this study are provided in ‘Additional file 4’ and can be retrieved from NCBI repository https://www.ncbi.nlm.nih.gov/sra/. Cattle reference sequences ARS_UCD1.2 is available at NCBI repository https://www.ncbi.nlm.nih.gov/genome/?term=cattle. Defined cattle ancestral alleles is available at https://tinyurl.com/cattle-aa. Custom R functions are available https://github.com/mas-agis/ances-al. Example of scripts for this paper including application of custom R functions is provided in [63].

## Abbreviations

AA: Ancestral allele
DA: Derived allele
BWA: Burrows-Wheeler Aligner
GO: Gene Ontology
NCBI: National Center for Biotechnology
SNP: Single-nucleotide polymorphism
