# Supplementary material for: Investigation of ancestral alleles in the Bovinae subfamily
Source: BMC Genomics. 2021 Feb 8;22:108. doi: 10.1186/s12864-021-07412-9 (PMC7871596; doi:10.1186/s12864-021-07412-9)
Supplement: Supplementary file 1 — Additional file 1. Phylogenetic trees from each chromosome [file 12864_2021_7412_MOESM1_ESM.pdf]

Additional files 1. Phylogenetic tree per chromosome

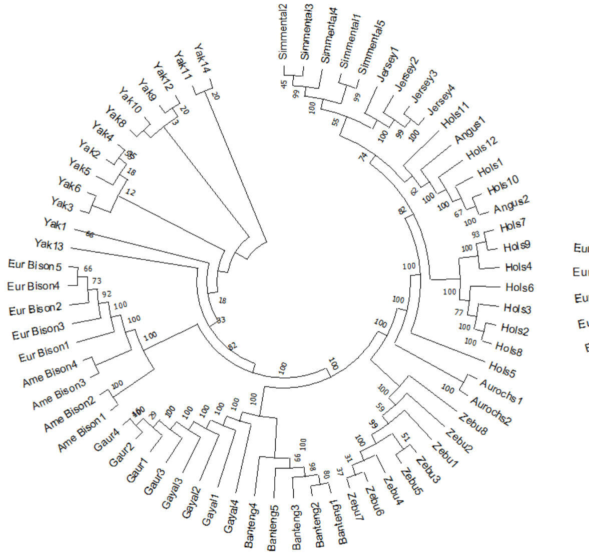

Chromosome 1

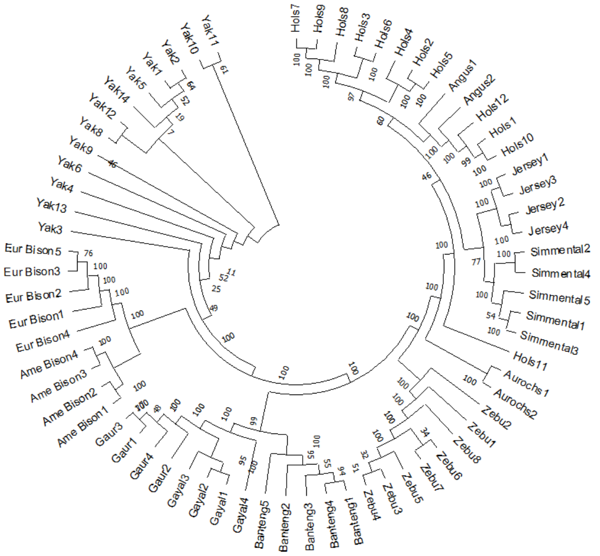

Chromosome 2

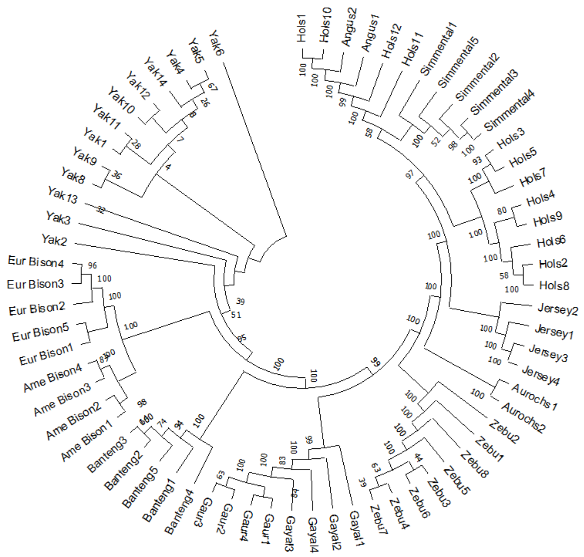

Chromosome 3

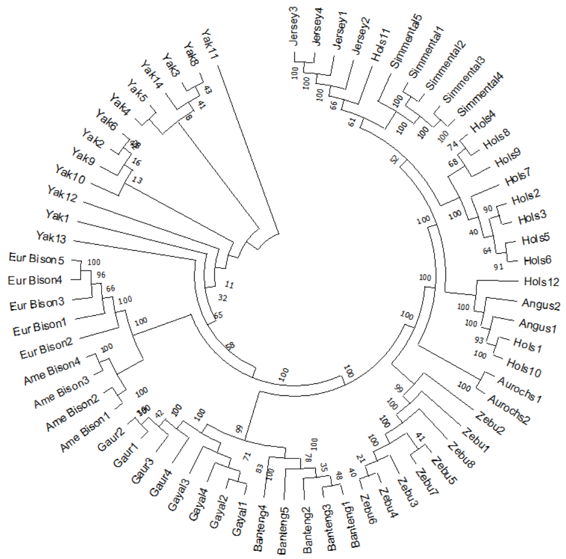

Chromosome 4

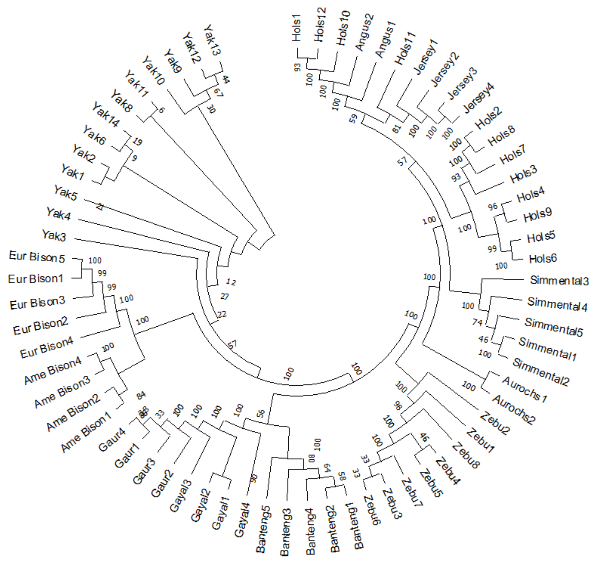

Chromosome 5

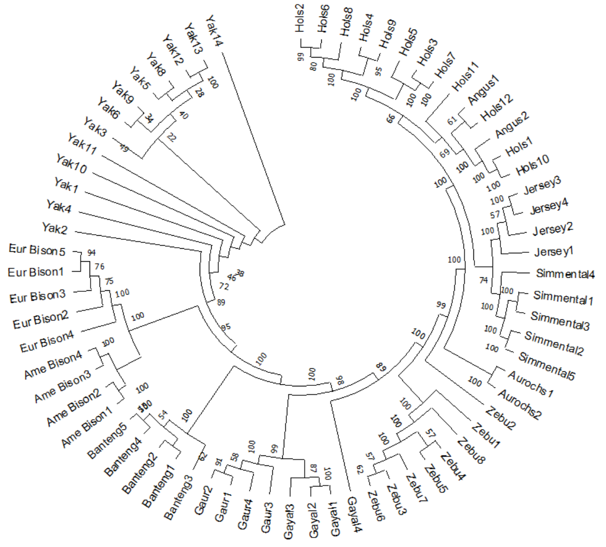

Chromosome 6

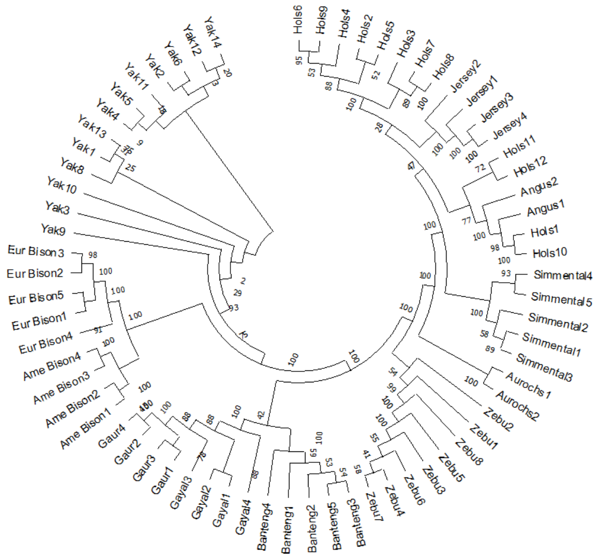

Chromosome 7

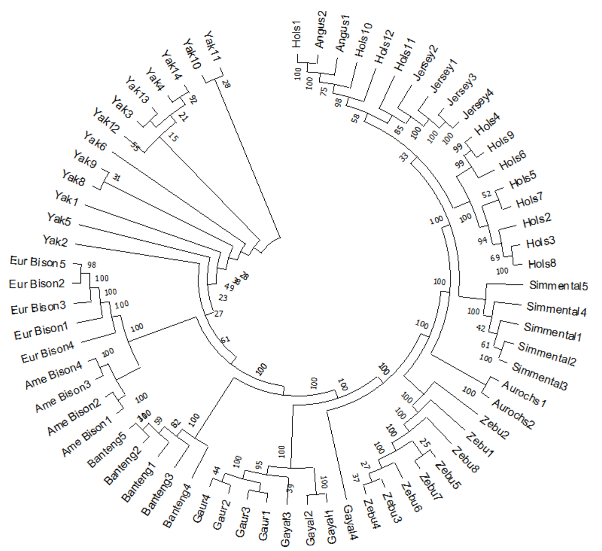

Chromosome 8

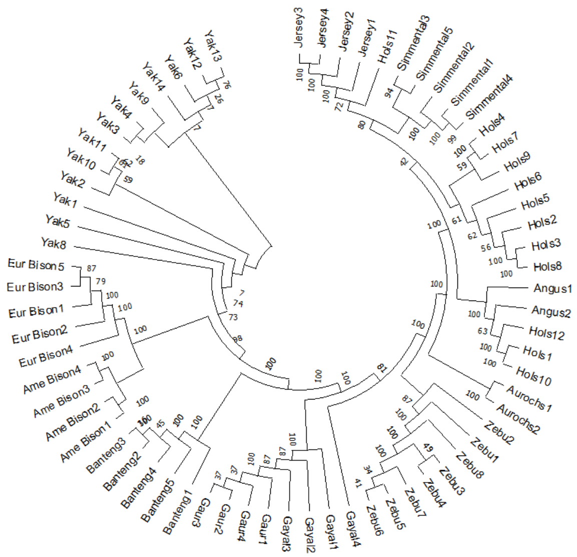

Chromosome 9

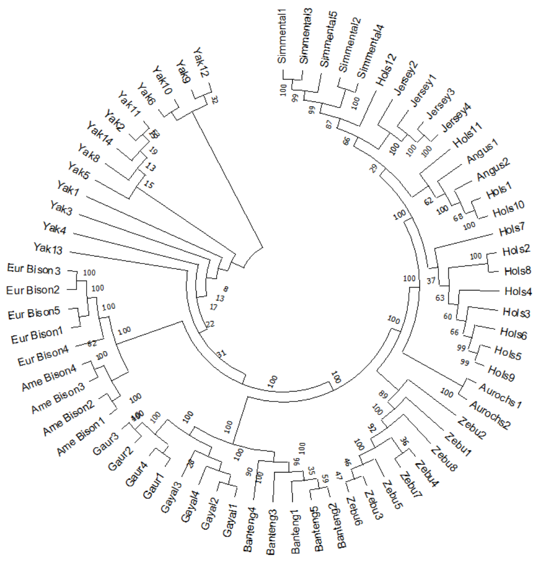

Chromosome 10

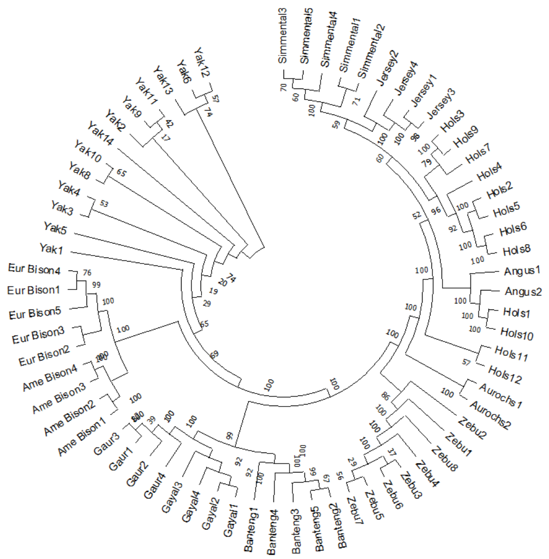

Chromosome 11

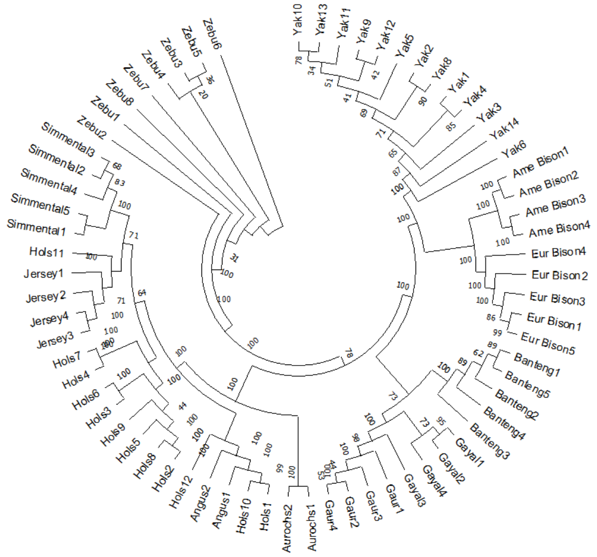

Chromosome 12

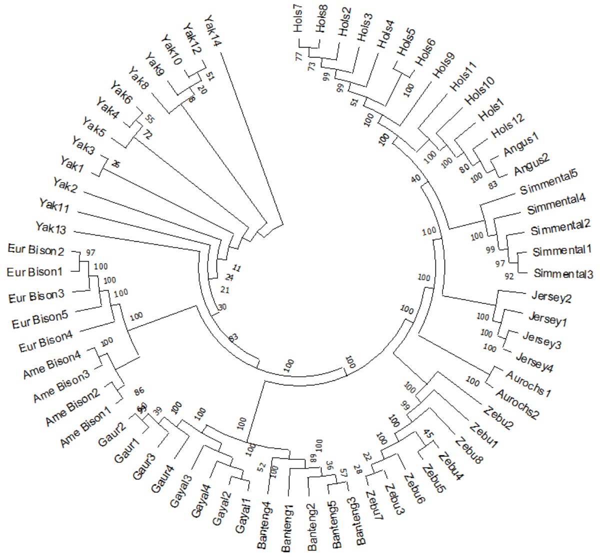

Chromosome 13

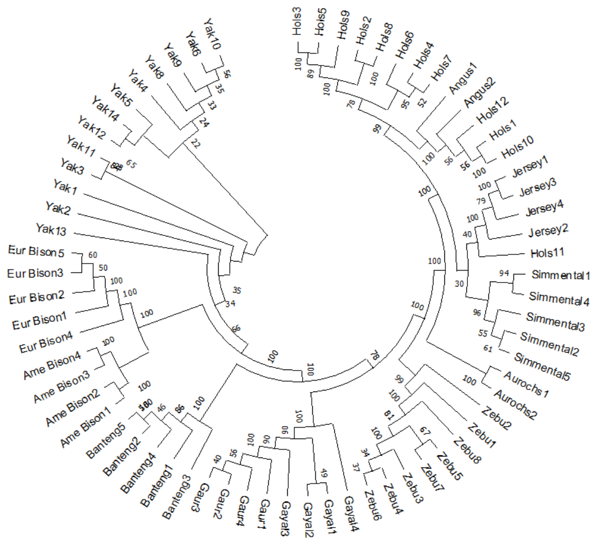

Chromosome 14

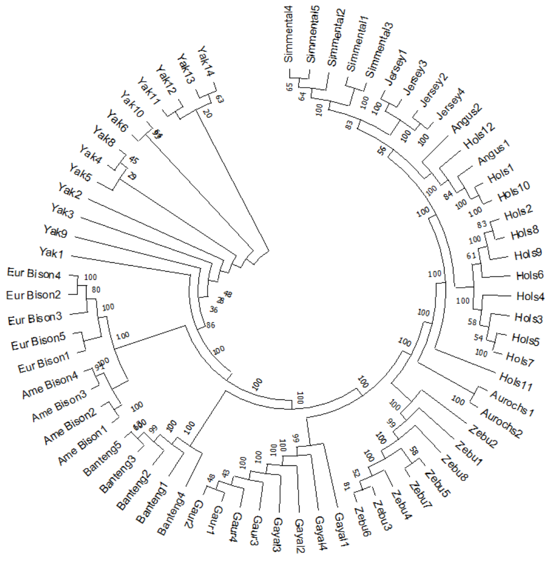

Chromosome 15

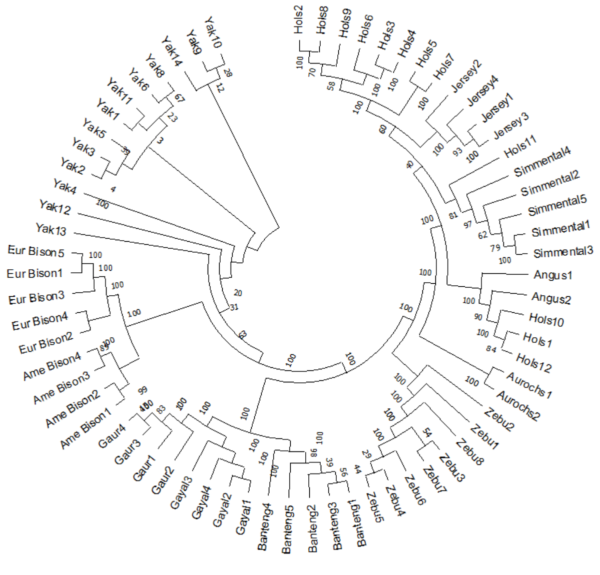

Chromosome 16

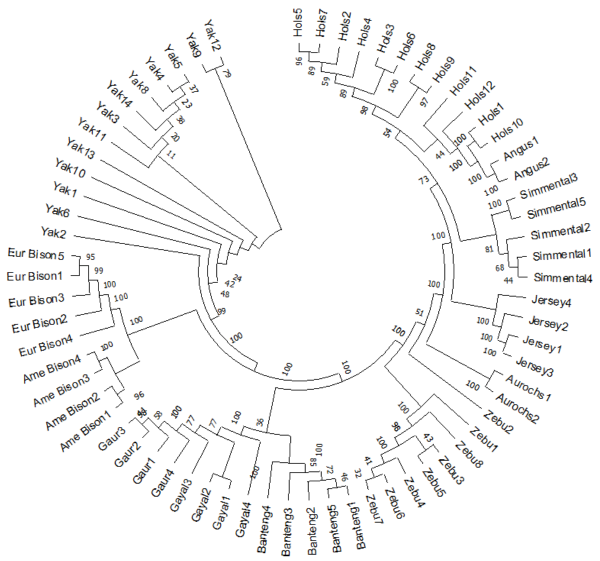

Chromosome 17

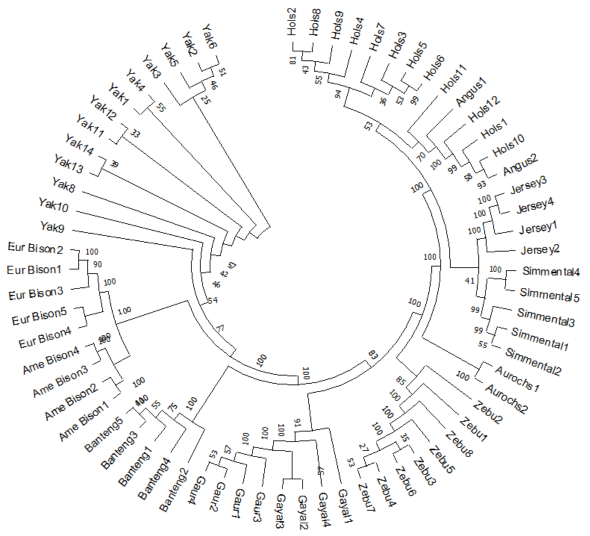

Chromosome 18

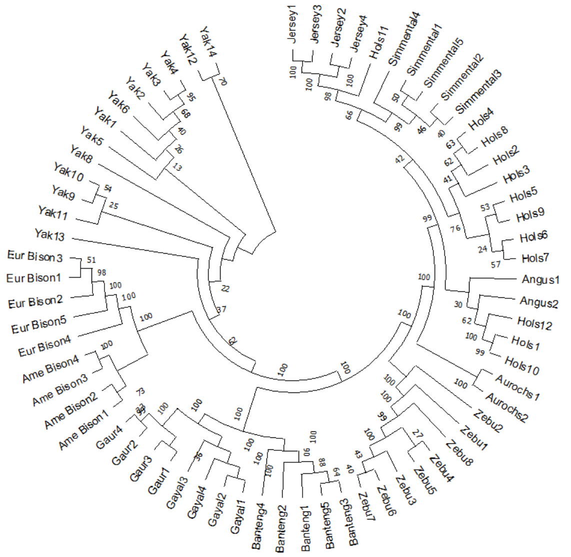

Chromosome 19

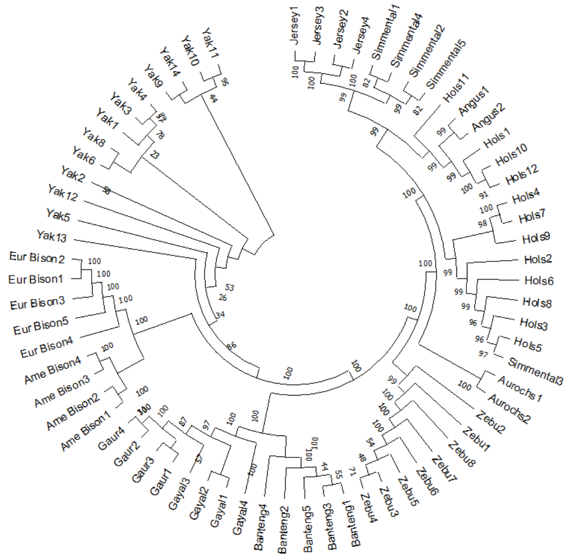

Chromosome 20

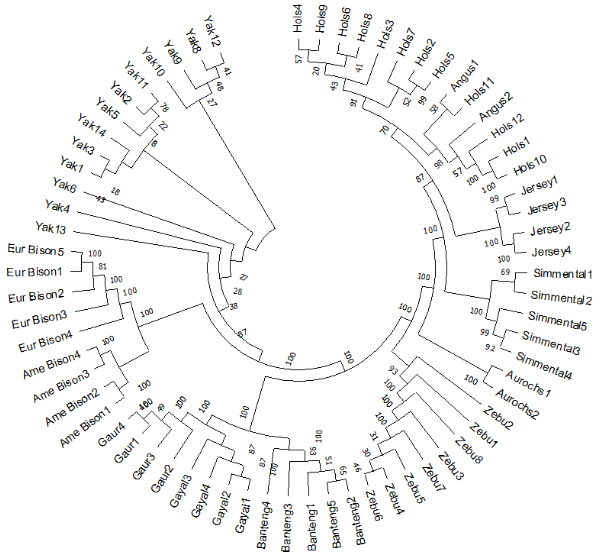

Chromosome 21

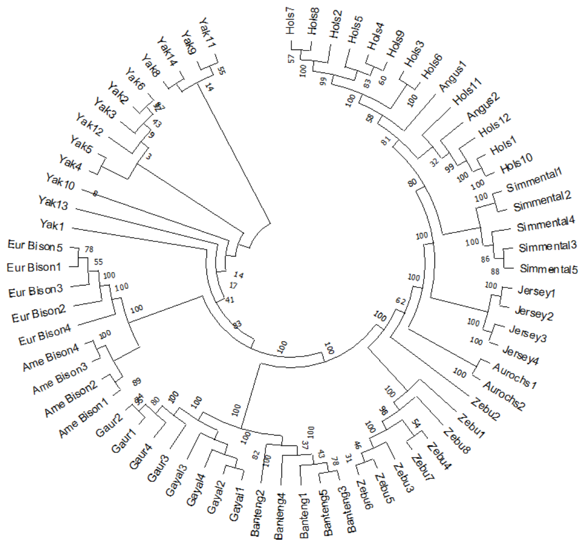

Chromosome 22

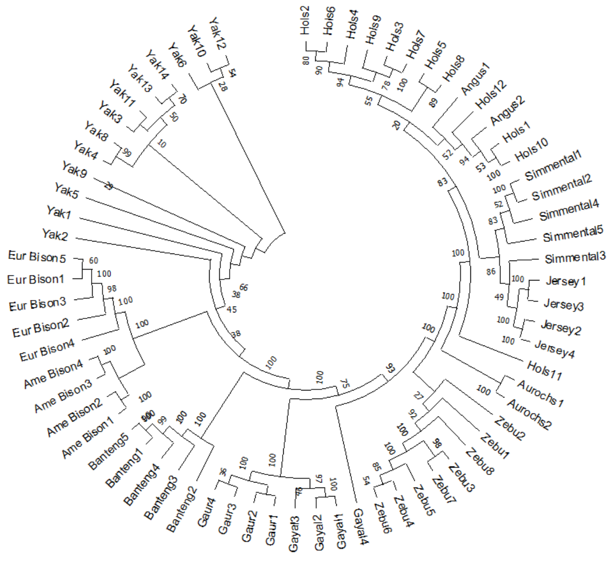

Chromosome 23

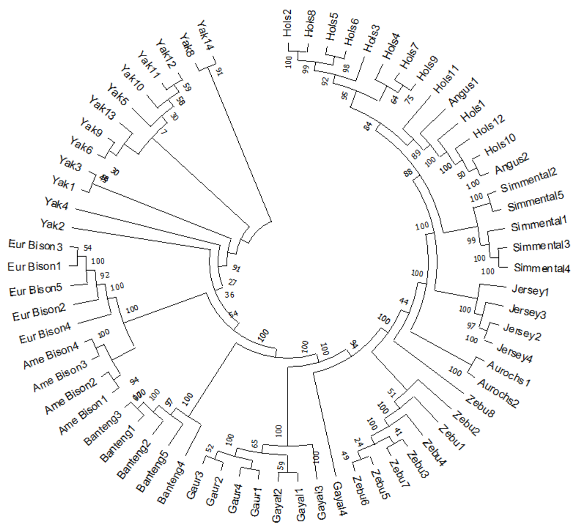

Chromosome 24

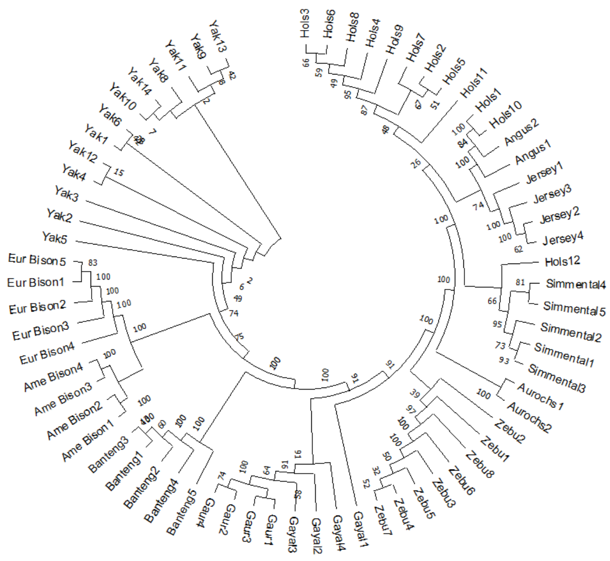

Chromosome 25

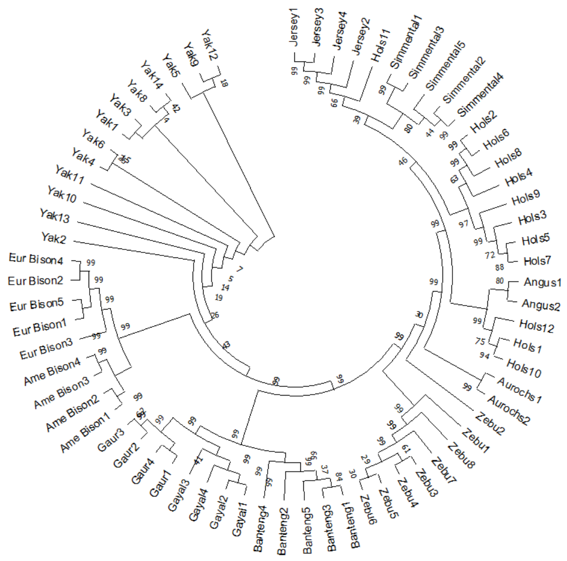

Chromosome 26

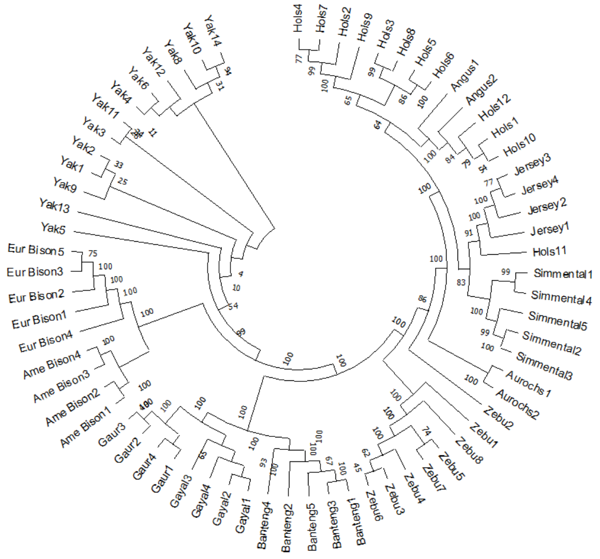

Chromosome 27

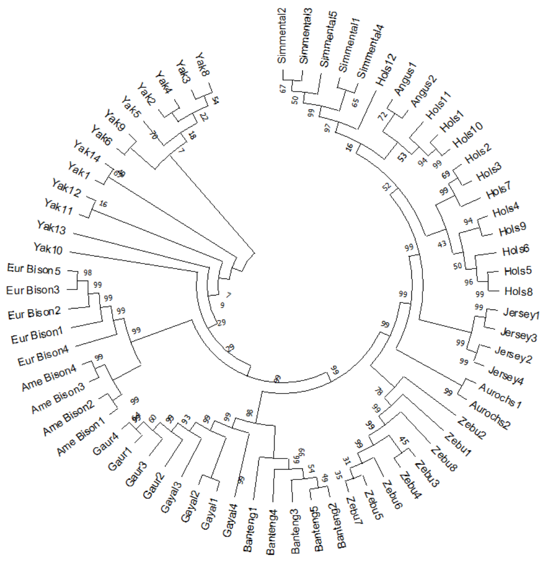

Chromosome 28

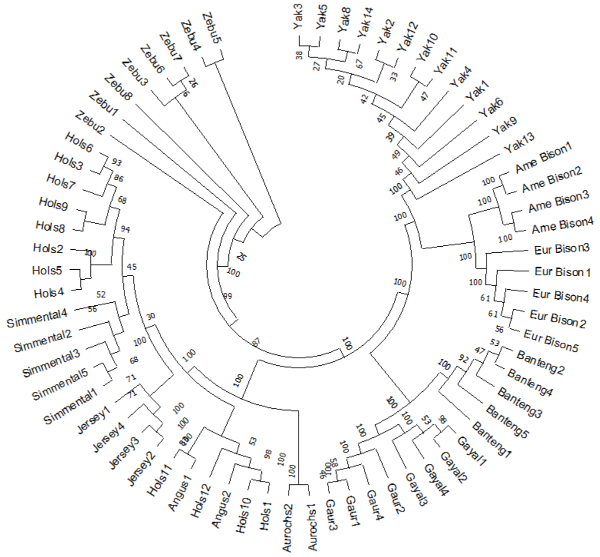

Chromosome 29
