# Supplementary material for: Investigation of ancestral alleles in the Bovinae subfamily
Source: BMC Genomics. 2021 Feb 8;22:108. doi: 10.1186/s12864-021-07412-9 (PMC7871596; doi:10.1186/s12864-021-07412-9)
Supplement: Supplementary file 2 — Additional file 2. Distribution of ancestral allele in all chromosomes of taurine and zebu [file 12864_2021_7412_MOESM2_ESM.pdf]

Additional files 3. Distribution of ancestral allele in all chromosomes of taurine (page 1-15) and zebu (15-29)

Taurine cattle

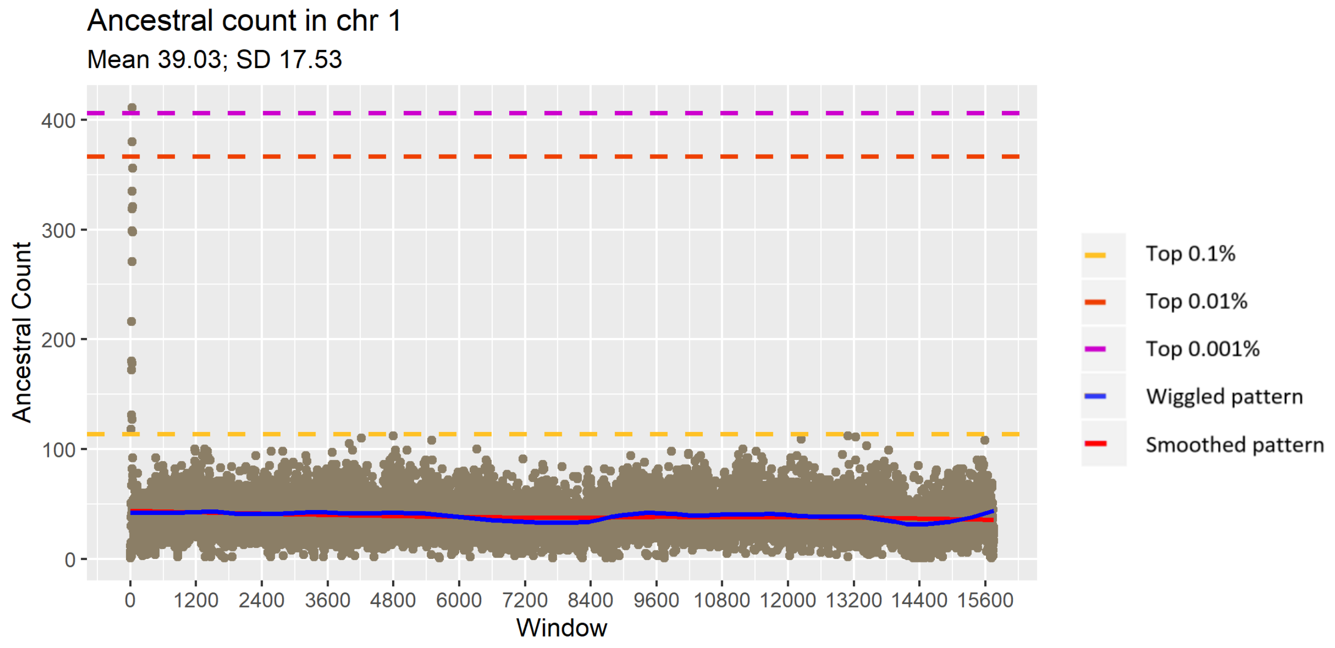

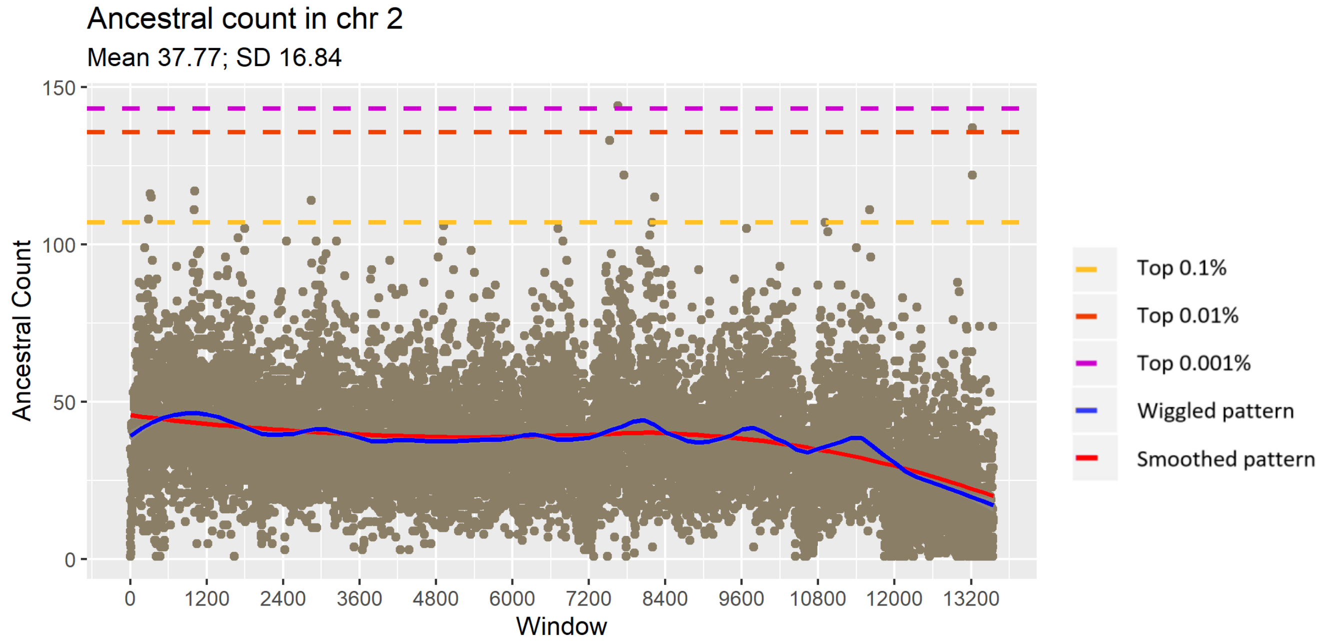

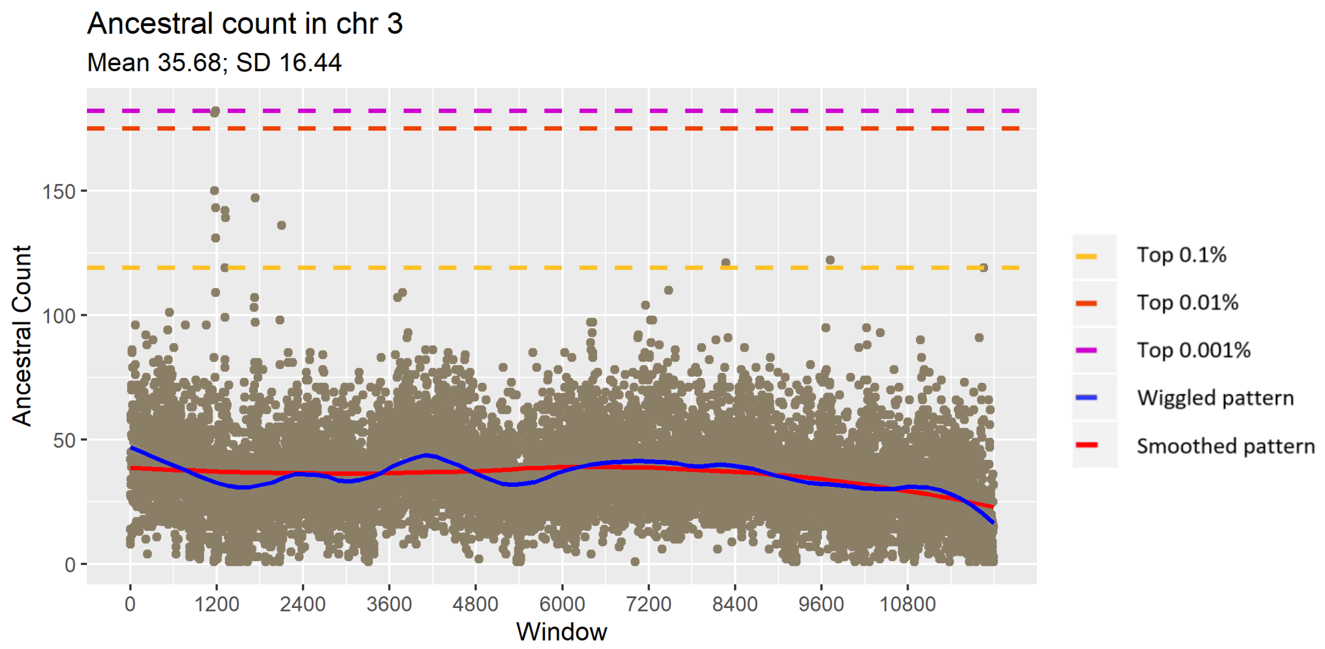
Ancestral count in chr 3
Mean 35.68; SD 16.44
Ancestral Count
0
50
100
150
0
1200
2400
3600
4800
6000
7200
8400
9600
10800
Window
Top 0.1%
Top 0.01%
Top 0.001%
Wiggled pattern
Smoothed pattern

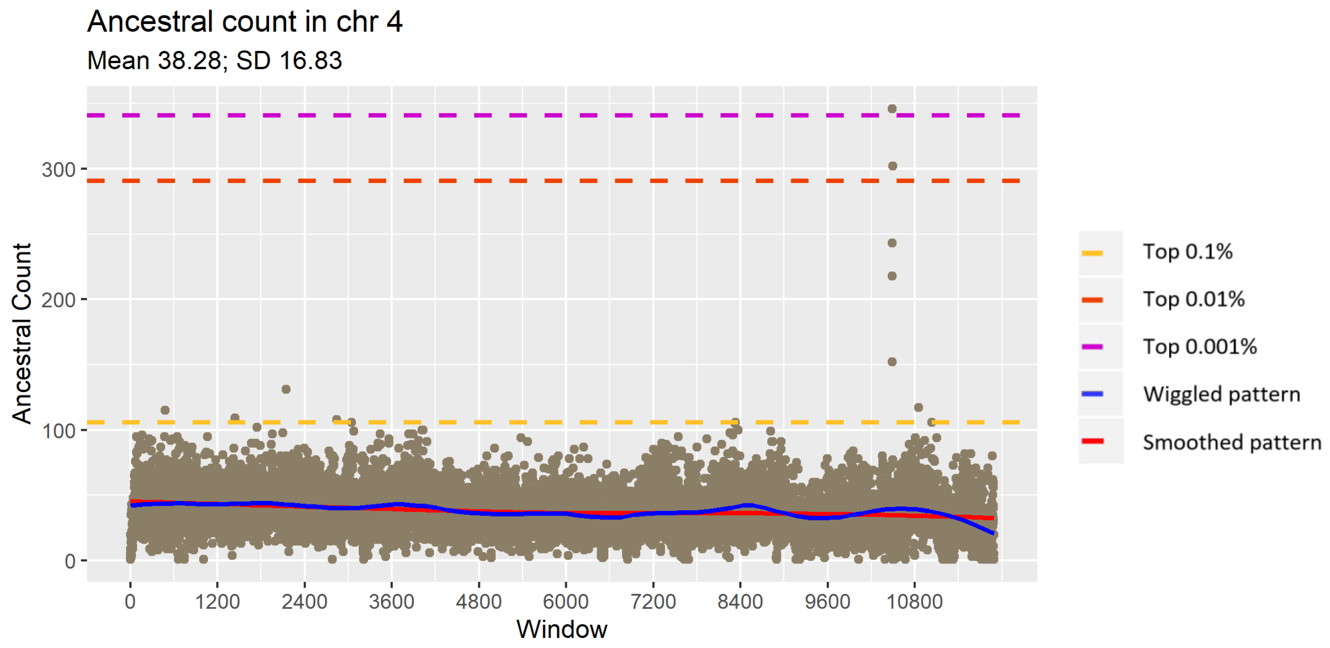
Ancestral count in chr 4
Mean 38.28; SD 16.83
Ancestral Count
0
100
200
300
0
1200
2400
3600
4800
6000
7200
8400
9600
10800
Window
Top 0.1%
Top 0.01%
Top 0.001%
Wiggled pattern
Smoothed pattern

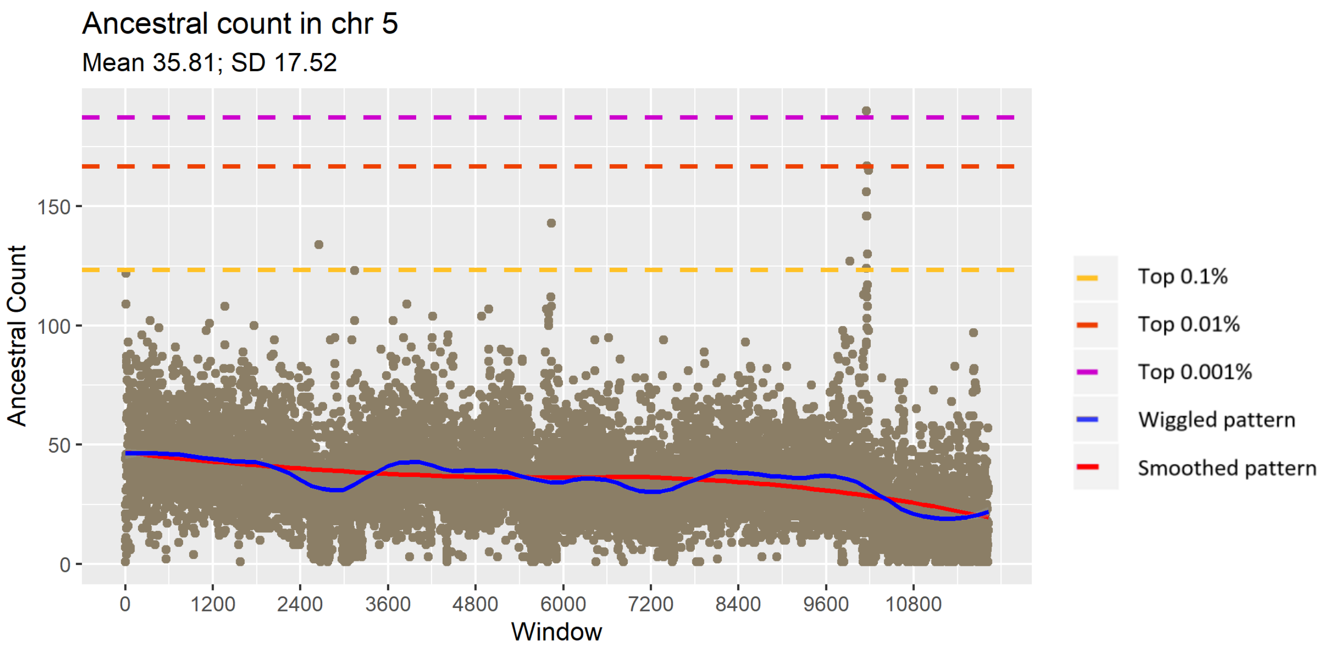
Ancestral count in chr 5
Mean 35.81; SD 17.52
Ancestral Count
Window
0
50
100
150
0
1200
2400
3600
4800
6000
7200
8400
9600
10800
Top 0.1%
Top 0.01%
Top 0.001%
Wiggled pattern
Smoothed pattern

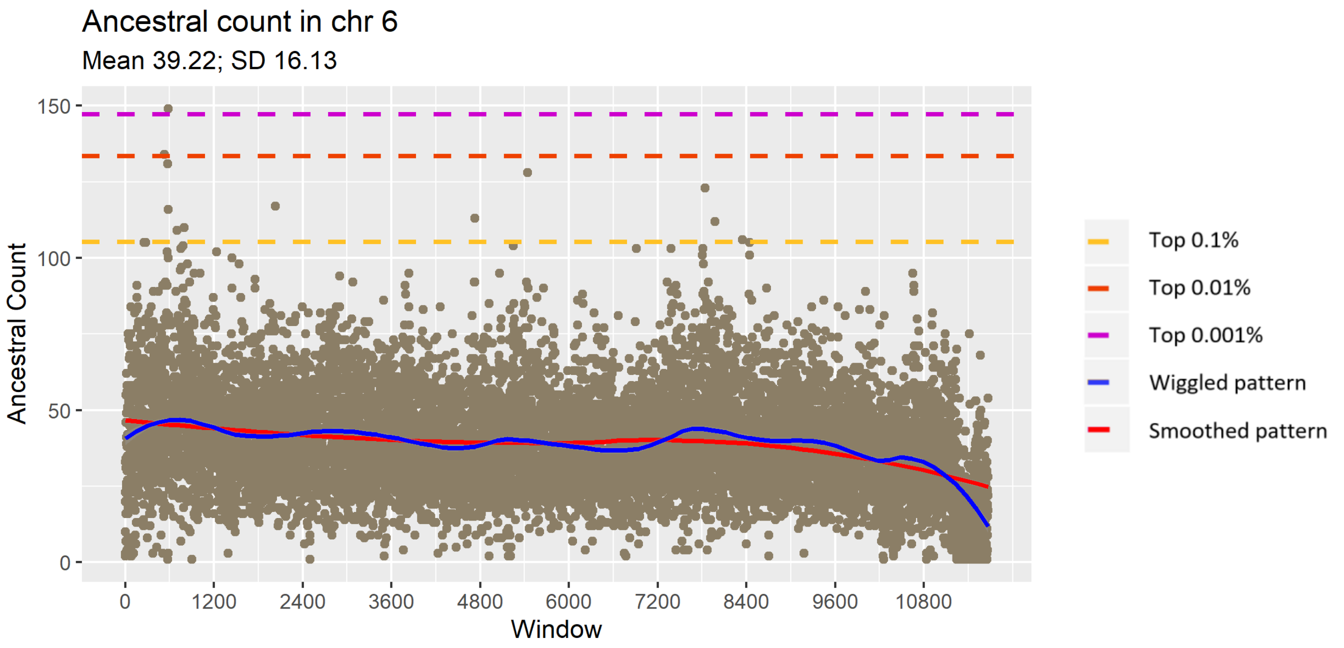
Ancestral count in chr 6
Mean 39.22; SD 16.13
Ancestral Count
Window
0
50
100
150
0
1200
2400
3600
4800
6000
7200
8400
9600
10800
Top 0.1%
Top 0.01%
Top 0.001%
Wiggled pattern
Smoothed pattern

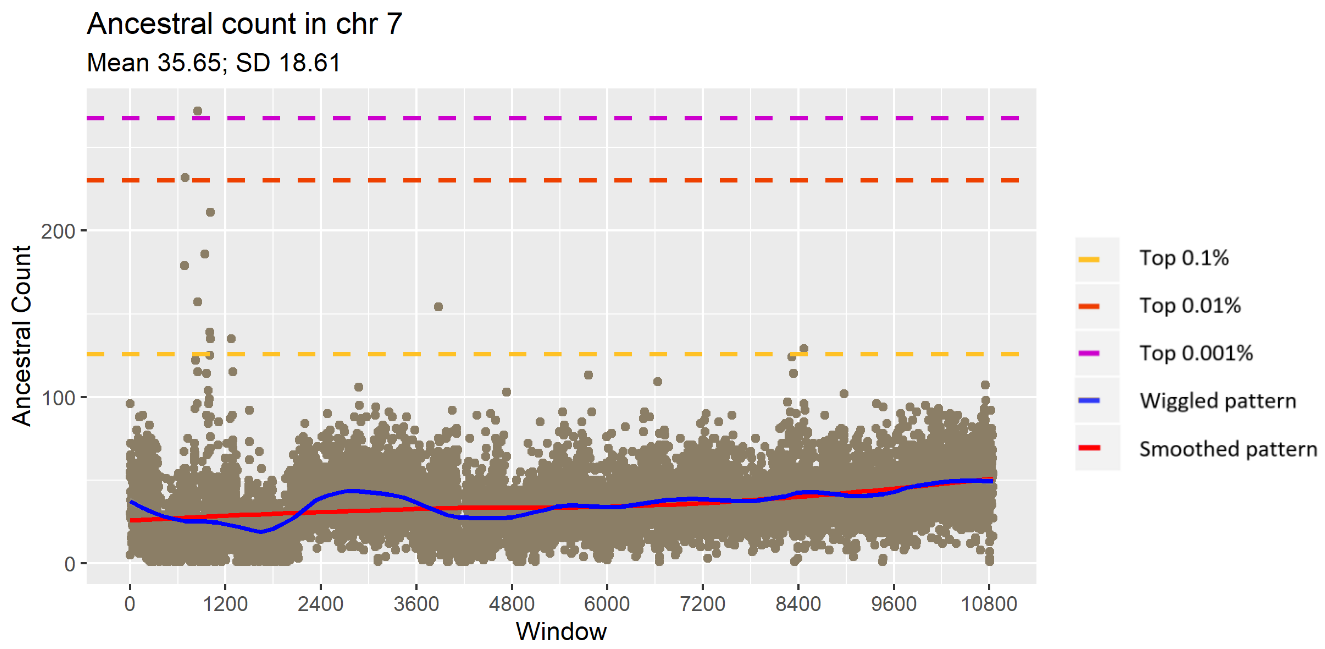

Ancestral count in chr 7
Mean 35.65; SD 18.61
Ancestral Count
200
100
0
0
1200
2400
3600
4800
6000
7200
8400
9600
10800
Window
Top 0.1%
Top 0.01%
Top 0.001%
Wiggled pattern
Smoothed pattern

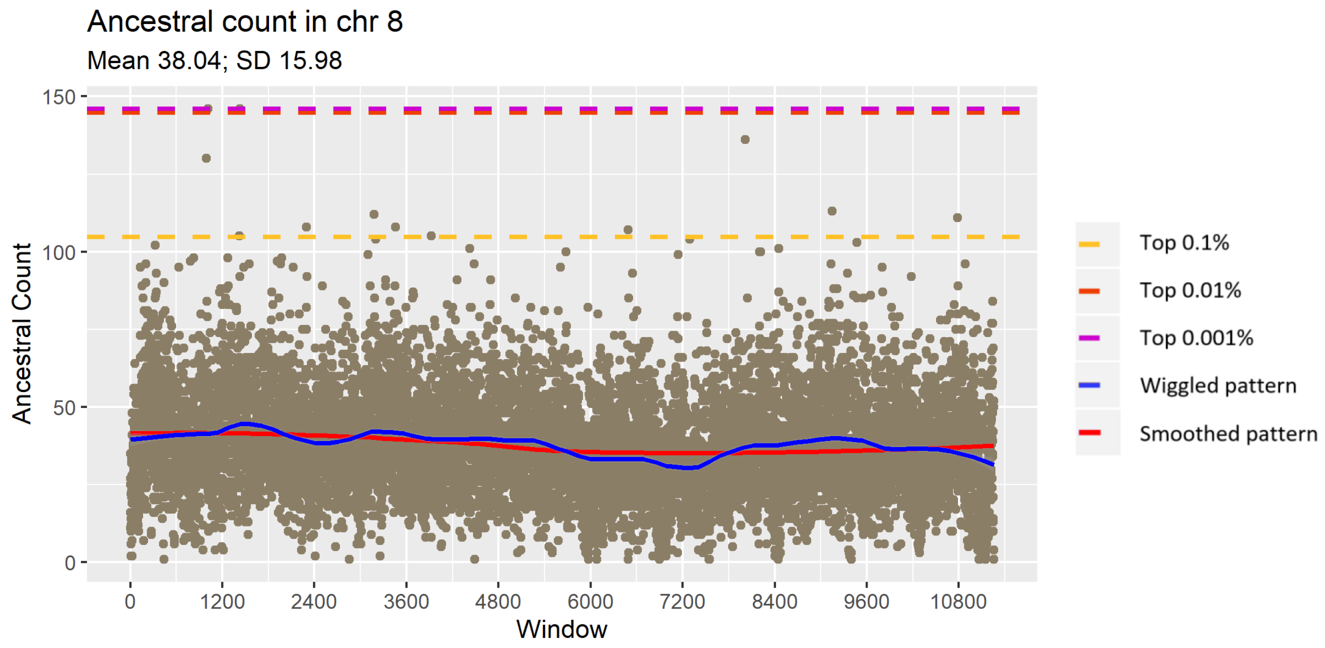

Ancestral count in chr 8
Mean 38.04; SD 15.98
Ancestral Count
150
100
50
0
0
1200
2400
3600
4800
6000
7200
8400
9600
10800
Window
Top 0.1%
Top 0.01%
Top 0.001%
Wiggled pattern
Smoothed pattern

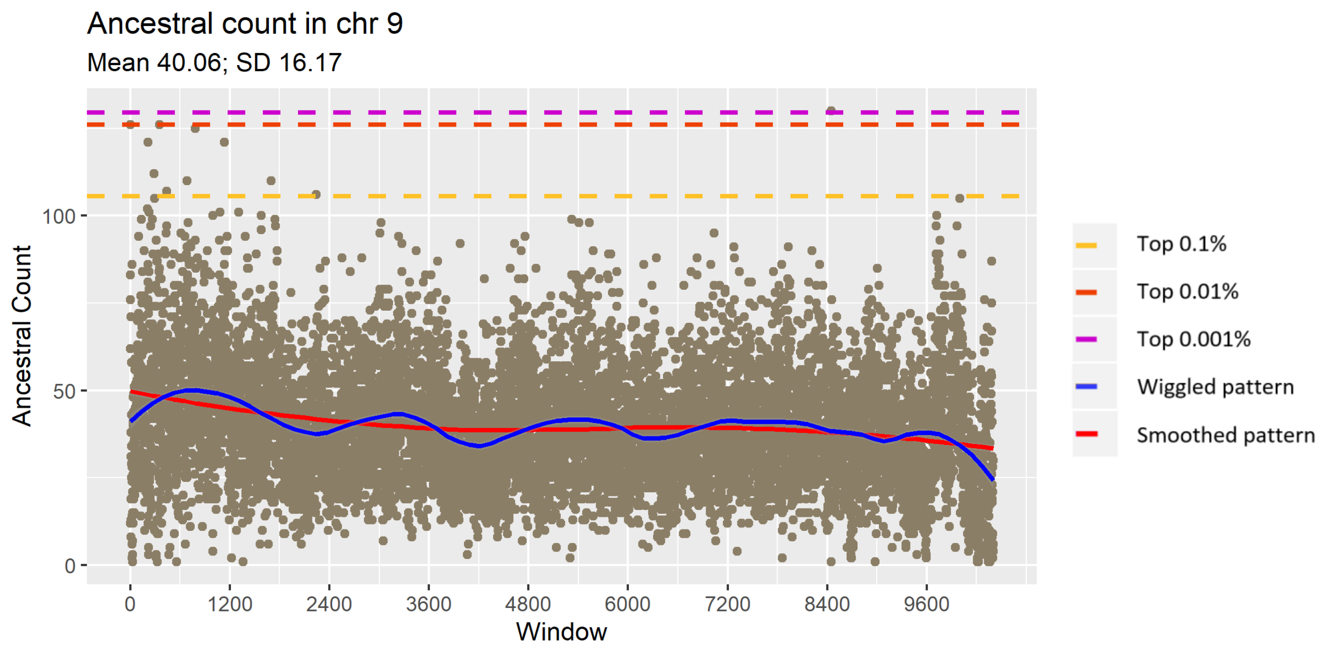
Ancestral count in chr 9
Mean 40.06; SD 16.17
Ancestral Count
100
50
0
0
1200
2400
3600
4800
6000
7200
8400
9600
Window
Top 0.1%
Top 0.01%
Top 0.001%
Wiggled pattern
Smoothed pattern

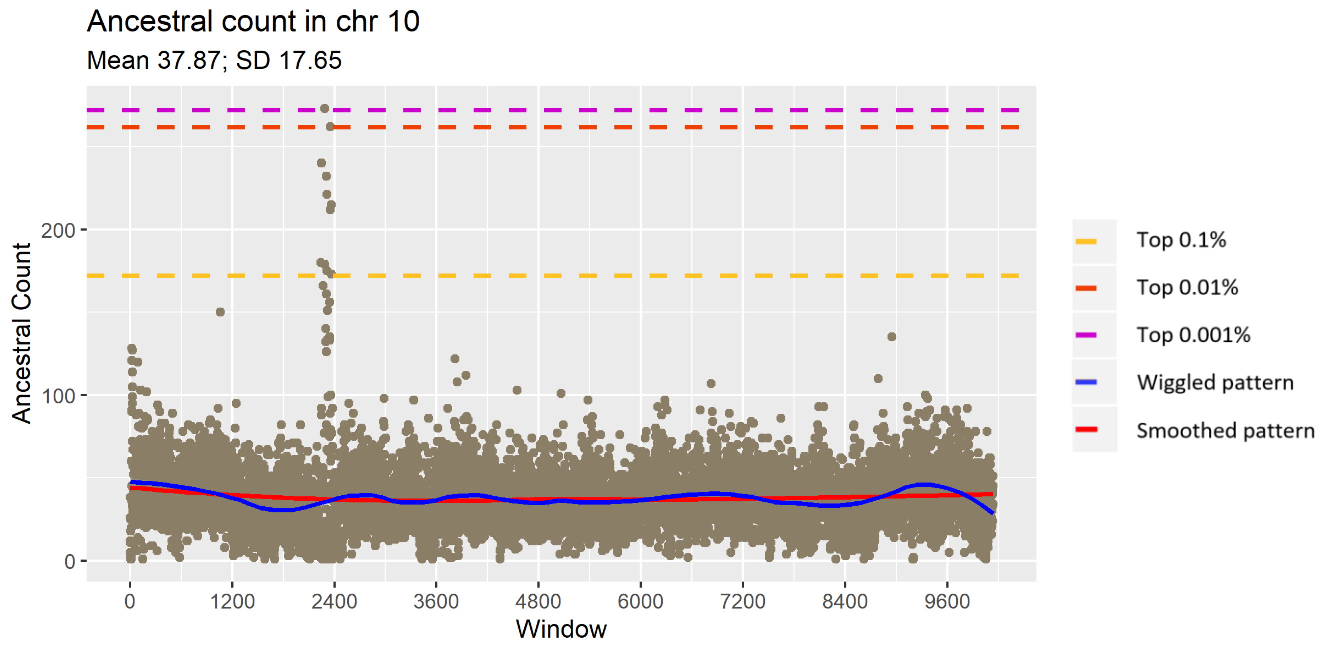
Ancestral count in chr 10
Mean 37.87; SD 17.65
Ancestral Count
200
100
0
0
1200
2400
3600
4800
6000
7200
8400
9600
Window
Top 0.1%
Top 0.01%
Top 0.001%
Wiggled pattern
Smoothed pattern

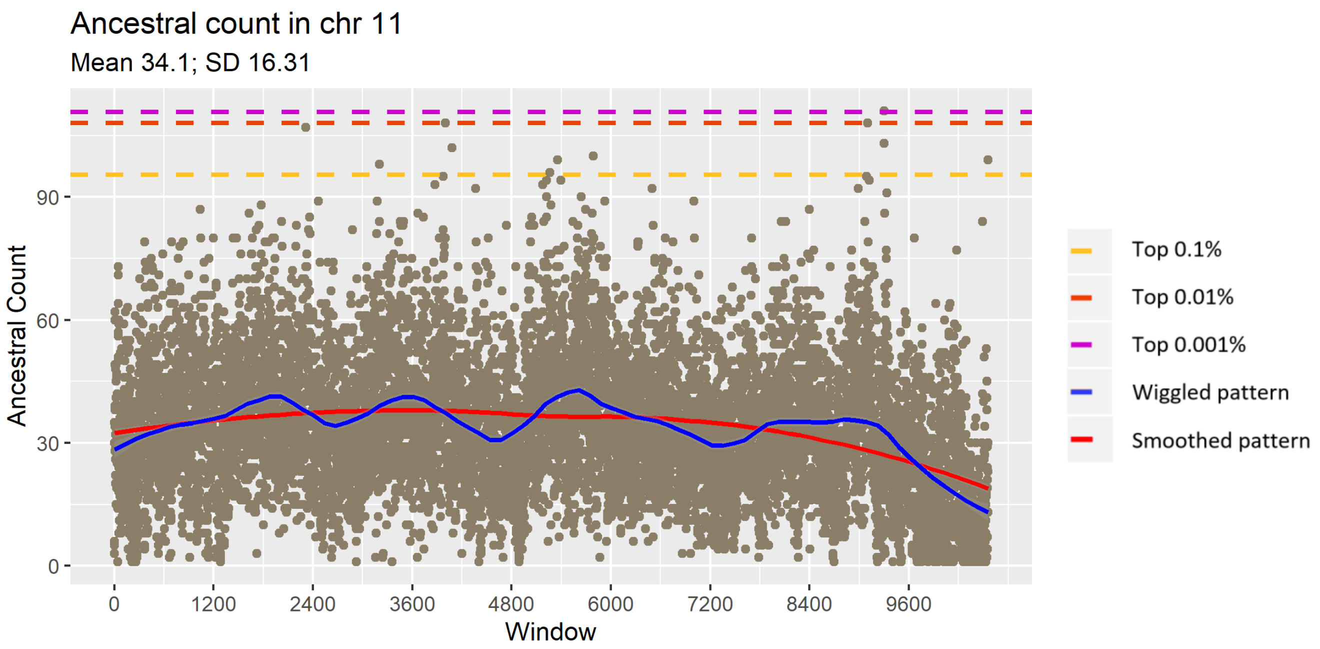
Ancestral count in chr 11
Mean 34.1; SD 16.31
Ancestral Count
0
30
60
90
0
1200
2400
3600
4800
6000
7200
8400
9600
Window
Top 0.1%
Top 0.01%
Top 0.001%
Wiggled pattern
Smoothed pattern

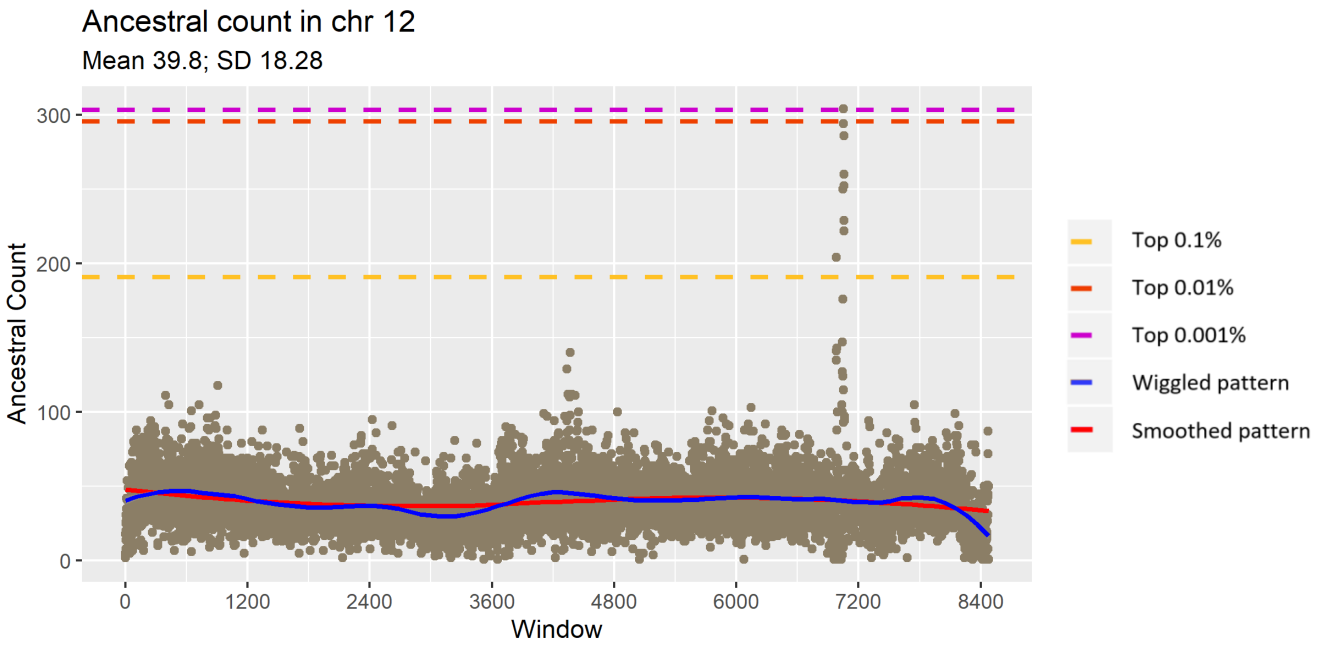
Ancestral count in chr 12
Mean 39.8; SD 18.28
Ancestral Count
0
100
200
300
0
1200
2400
3600
4800
6000
7200
8400
Window
Top 0.1%
Top 0.01%
Top 0.001%
Wiggled pattern
Smoothed pattern

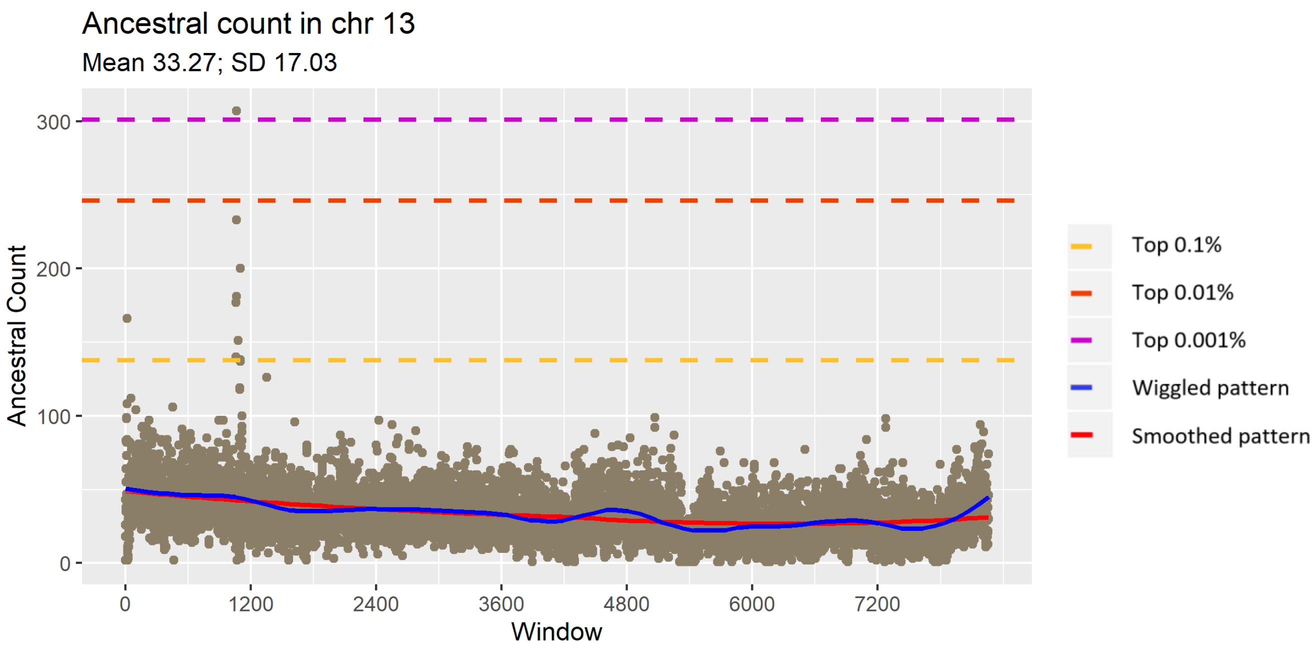
Ancestral count in chr 13
Mean 33.27; SD 17.03
Ancestral Count
Window
Top 0.1%
Top 0.01%
Top 0.001%
Wiggled pattern
Smoothed pattern

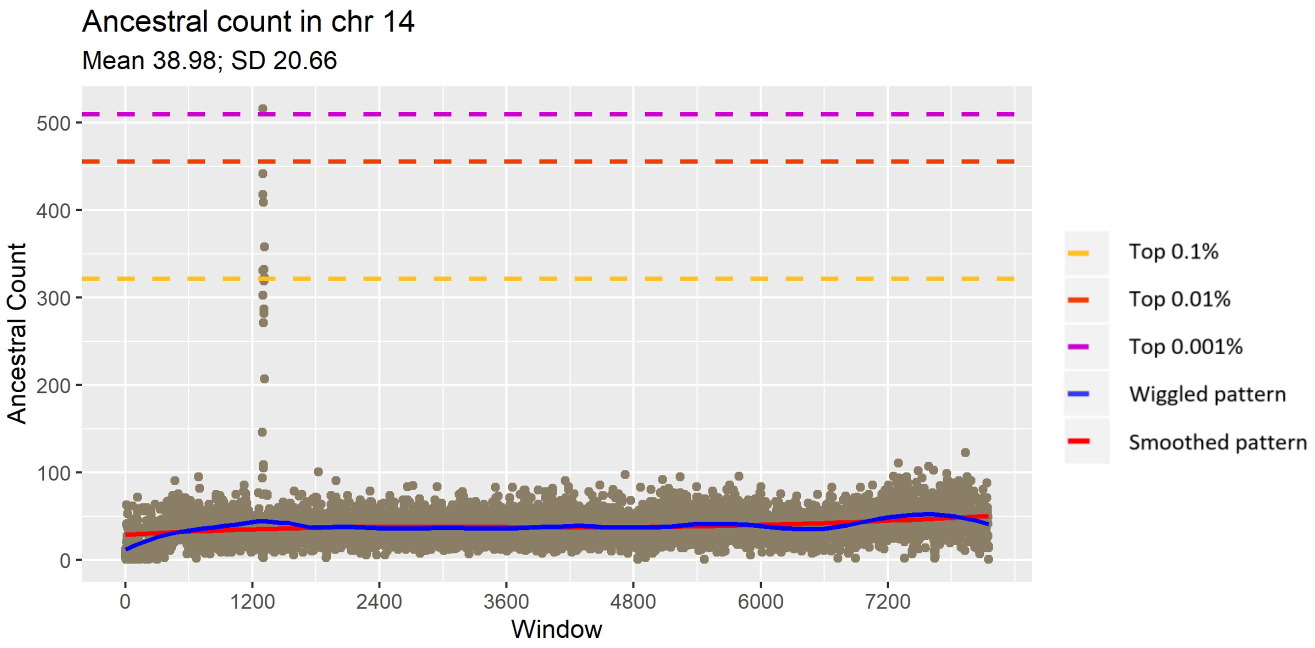
Ancestral count in chr 14
Mean 38.98; SD 20.66
Ancestral Count
Window
Top 0.1%
Top 0.01%
Top 0.001%
Wiggled pattern
Smoothed pattern

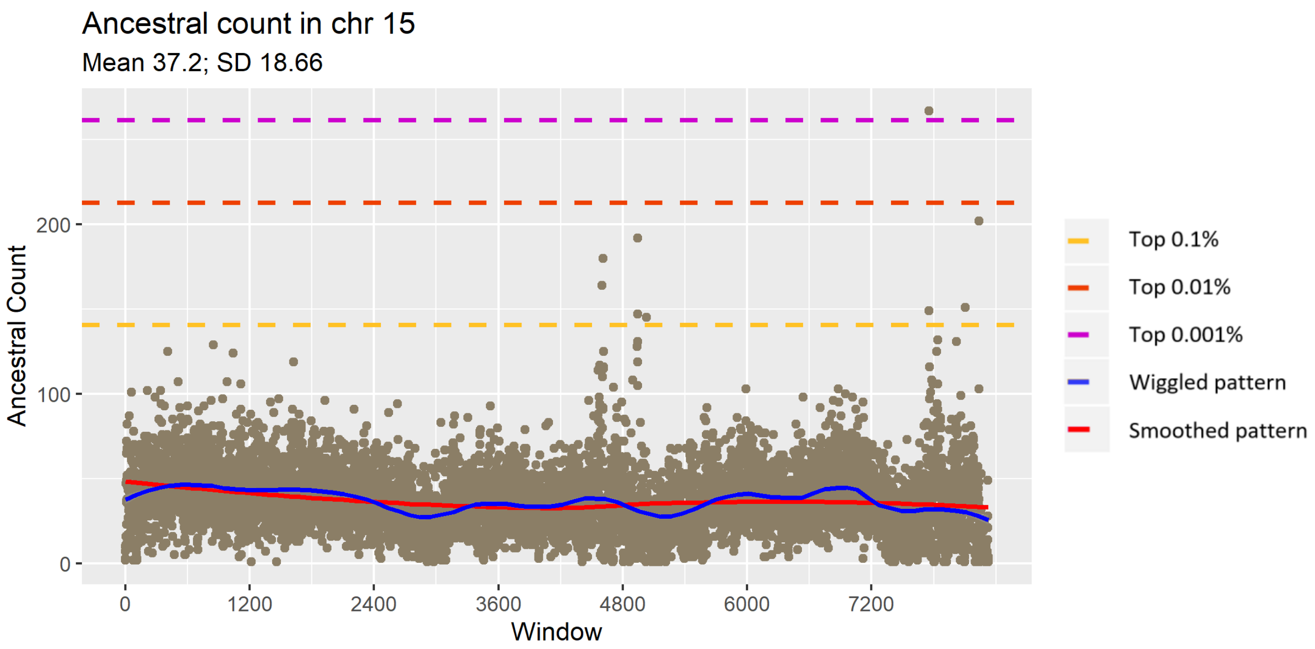
Ancestral count in chr 15
Mean 37.2; SD 18.66
Ancestral Count
0
100
200
0
1200
2400
3600
4800
6000
7200
Window
Top 0.1%
Top 0.01%
Top 0.001%
Wiggled pattern
Smoothed pattern

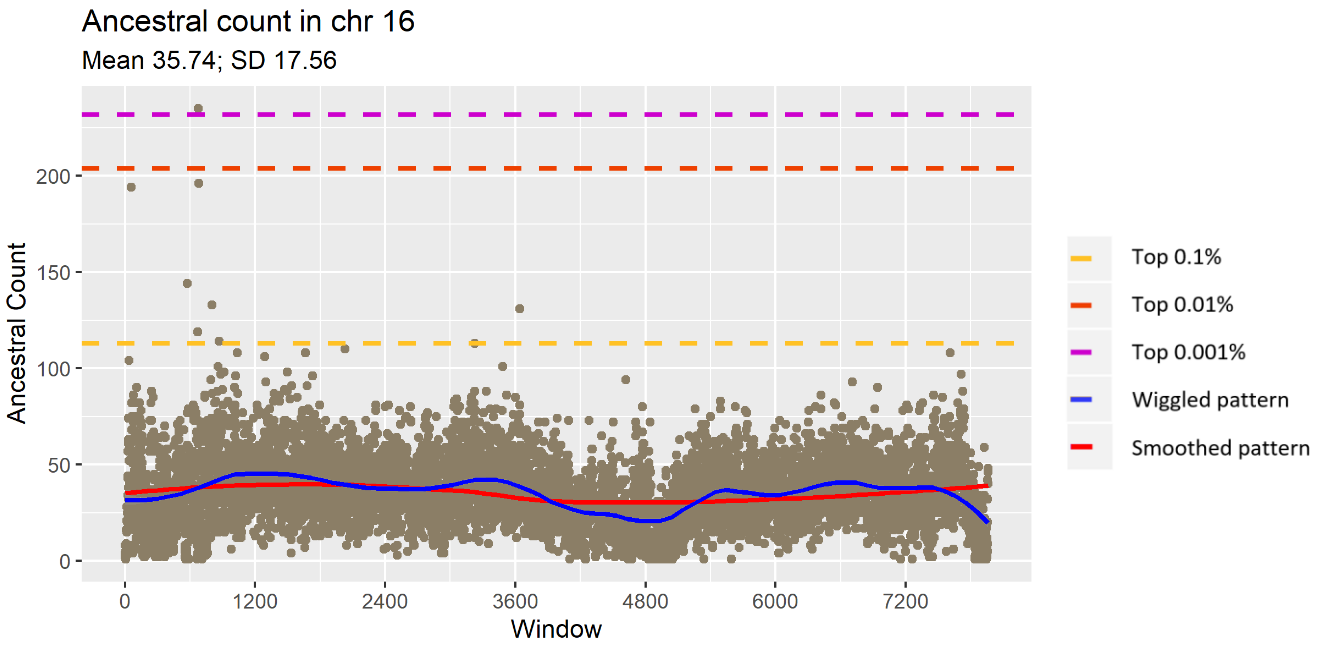
Ancestral count in chr 16
Mean 35.74; SD 17.56
Ancestral Count
0
50
100
150
200
0
1200
2400
3600
4800
6000
7200
Window
Top 0.1%
Top 0.01%
Top 0.001%
Wiggled pattern
Smoothed pattern

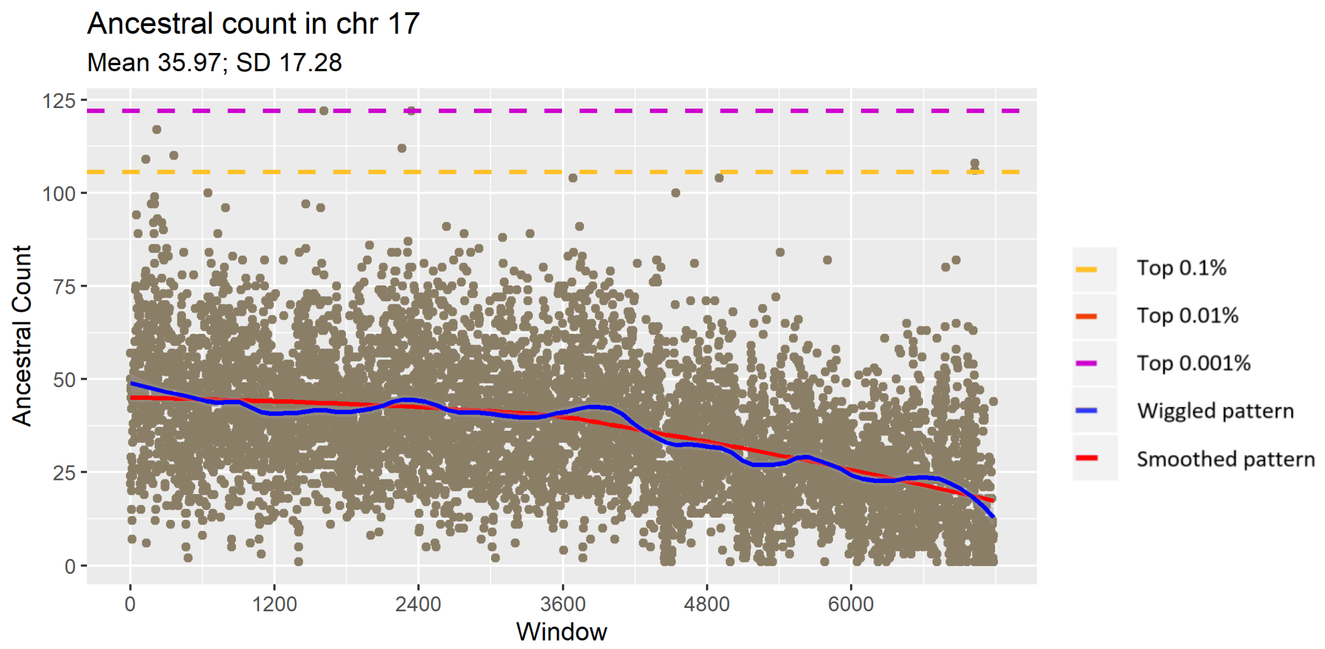

Ancestral count in chr 17
Mean 35.97; SD 17.28
125
100
75
50
25
0
Ancestral Count
0
1200
2400
3600
4800
6000
Window
Top 0.1%
Top 0.01%
Top 0.001%
Wiggled pattern
Smoothed pattern

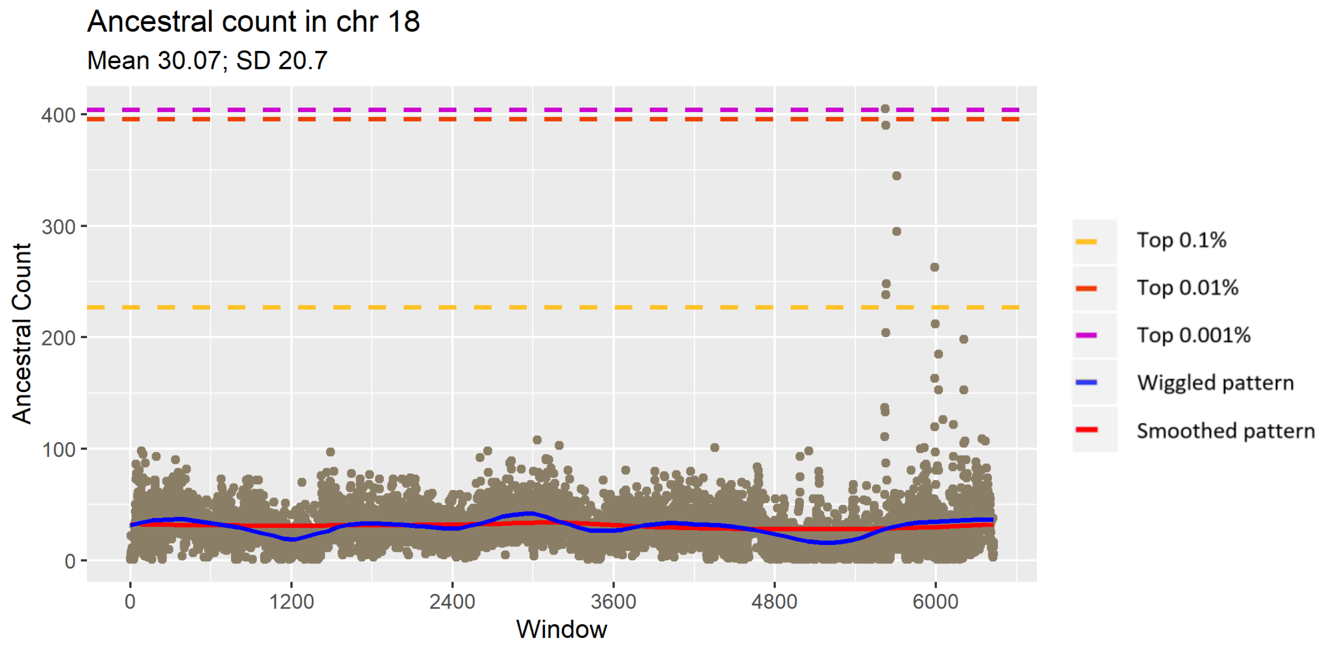

Ancestral count in chr 18
Mean 30.07; SD 20.7
400
300
200
100
0
Ancestral Count
0
1200
2400
3600
4800
6000
Window
Top 0.1%
Top 0.01%
Top 0.001%
Wiggled pattern
Smoothed pattern

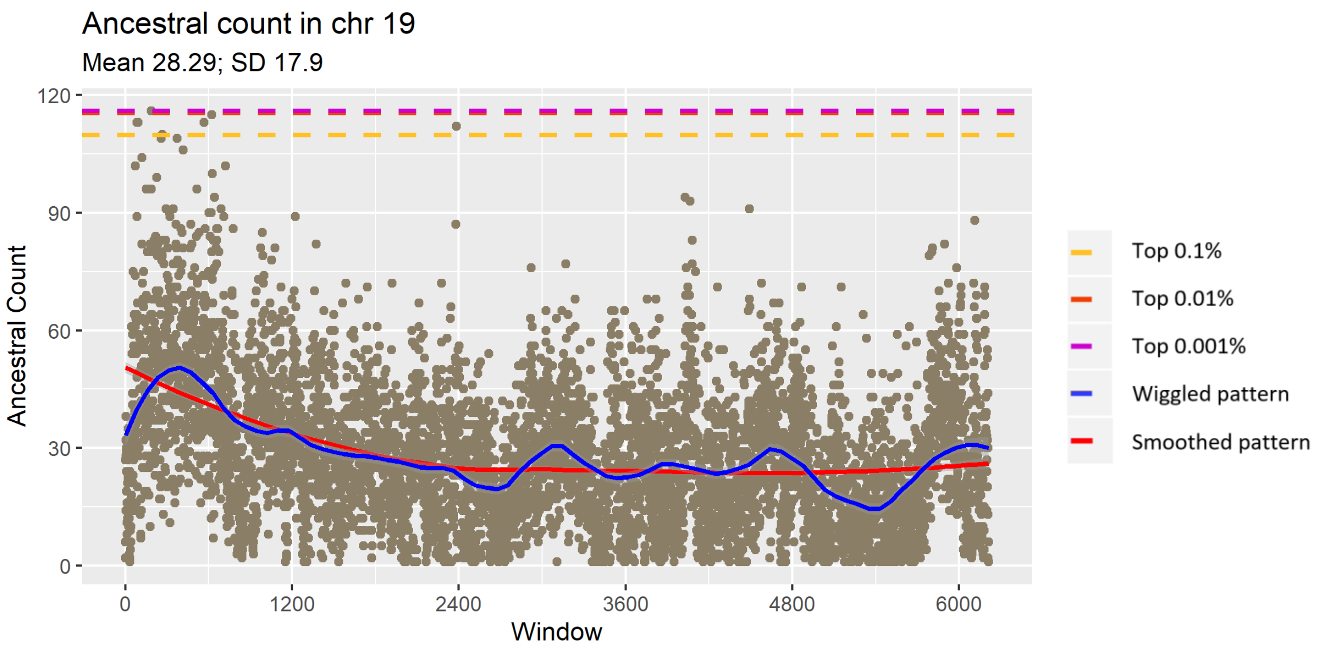
Ancestral count in chr 19
Mean 28.29; SD 17.9
Ancestral Count
Window
0
30
60
90
120
0
1200
2400
3600
4800
6000
Top 0.1%
Top 0.01%
Top 0.001%
Wiggled pattern
Smoothed pattern

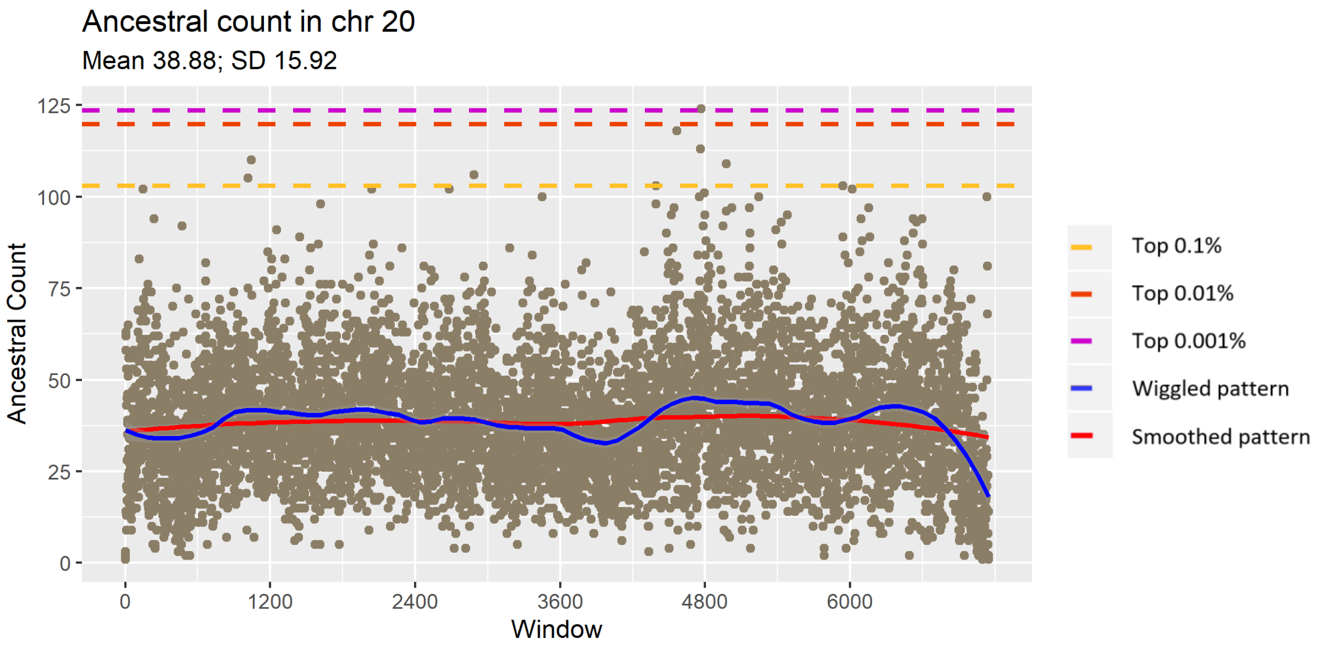
Ancestral count in chr 20
Mean 38.88; SD 15.92
Ancestral Count
Window
0
25
50
75
100
125
0
1200
2400
3600
4800
6000
Top 0.1%
Top 0.01%
Top 0.001%
Wiggled pattern
Smoothed pattern

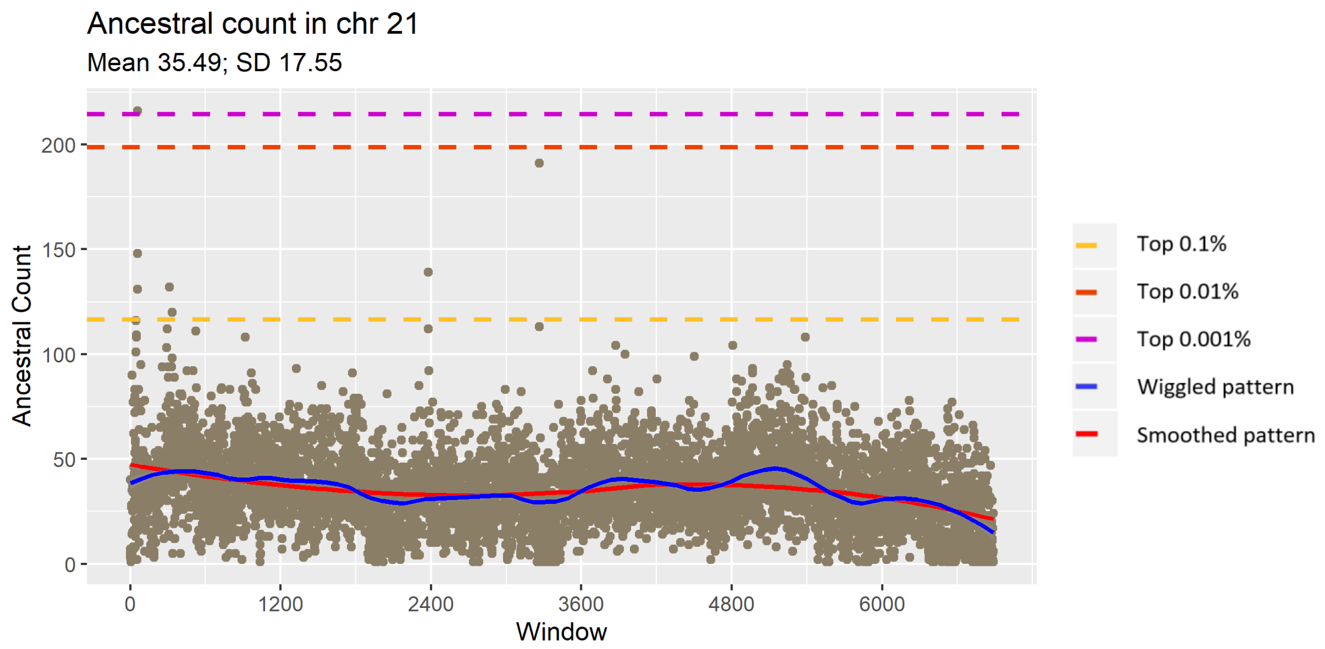
Ancestral count in chr 21
Mean 35.49; SD 17.55
Ancestral Count
0
50
100
150
200
0
1200
2400
3600
4800
6000
Window
Top 0.1%
Top 0.01%
Top 0.001%
Wiggled pattern
Smoothed pattern

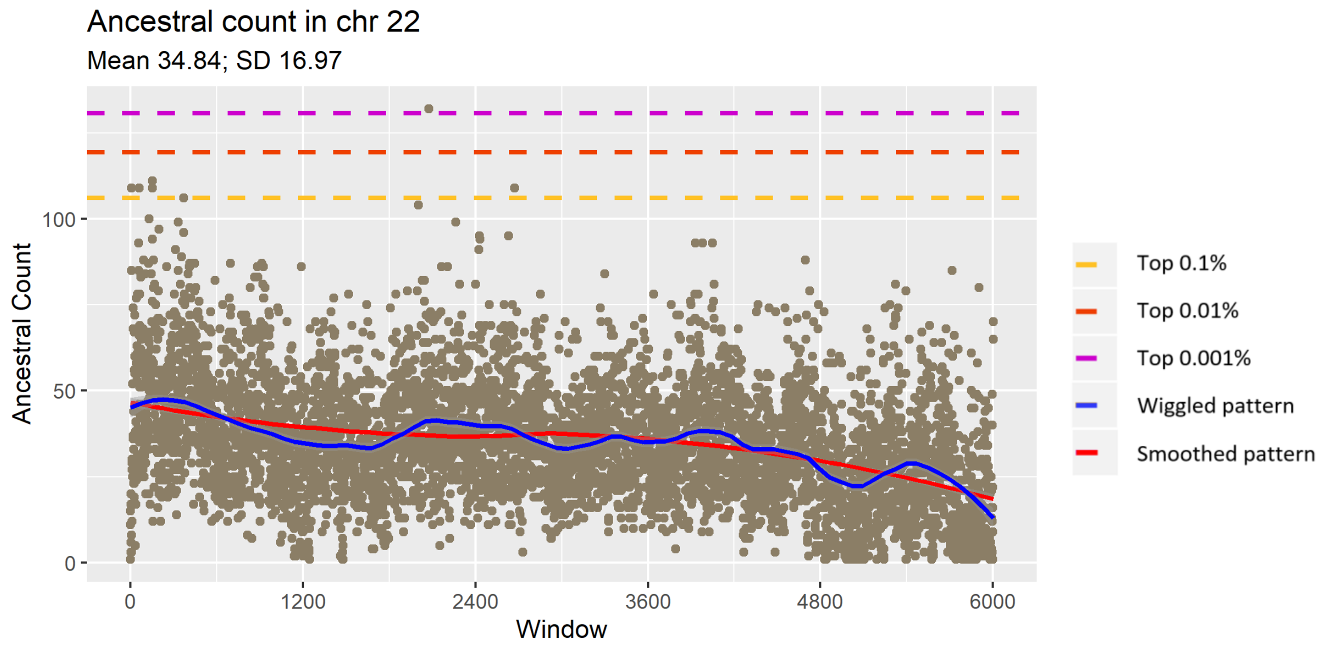
Ancestral count in chr 22
Mean 34.84; SD 16.97
Ancestral Count
0
50
100
0
1200
2400
3600
4800
6000
Window
Top 0.1%
Top 0.01%
Top 0.001%
Wiggled pattern
Smoothed pattern

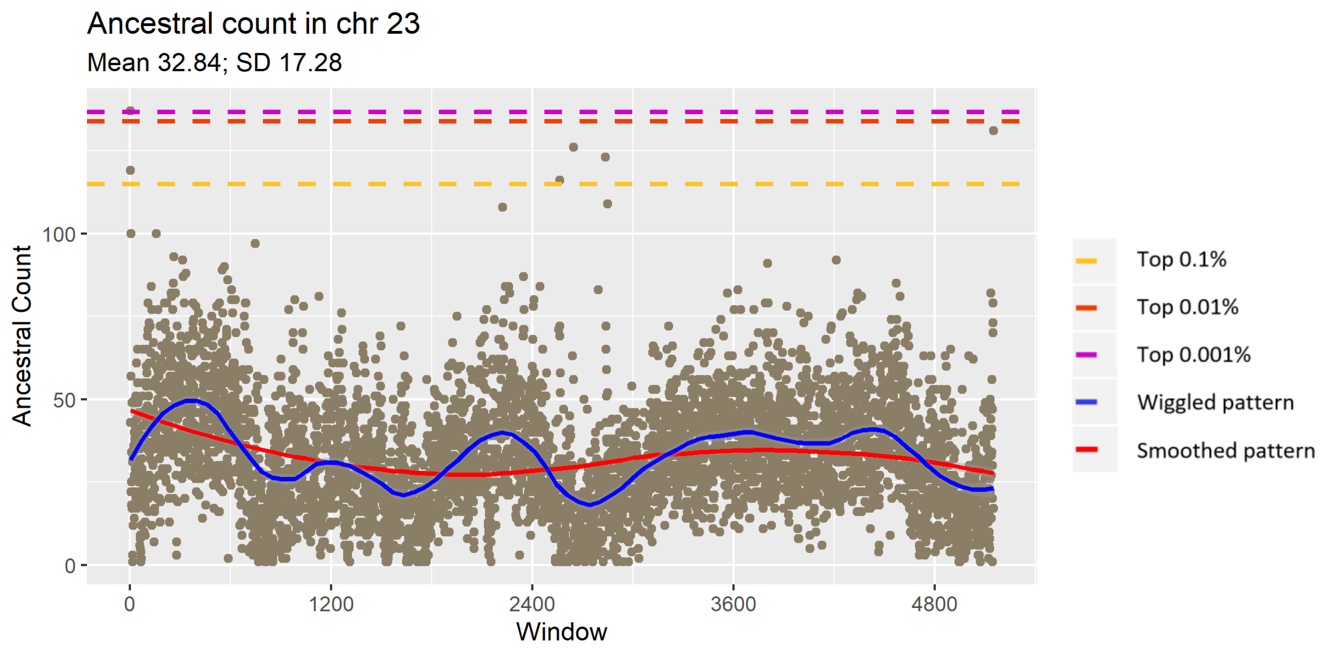
Ancestral count in chr 23
Mean 32.84; SD 17.28
Ancestral Count
Window
0
50
100
0
1200
2400
3600
4800
Top 0.1%
Top 0.01%
Top 0.001%
Wiggled pattern
Smoothed pattern

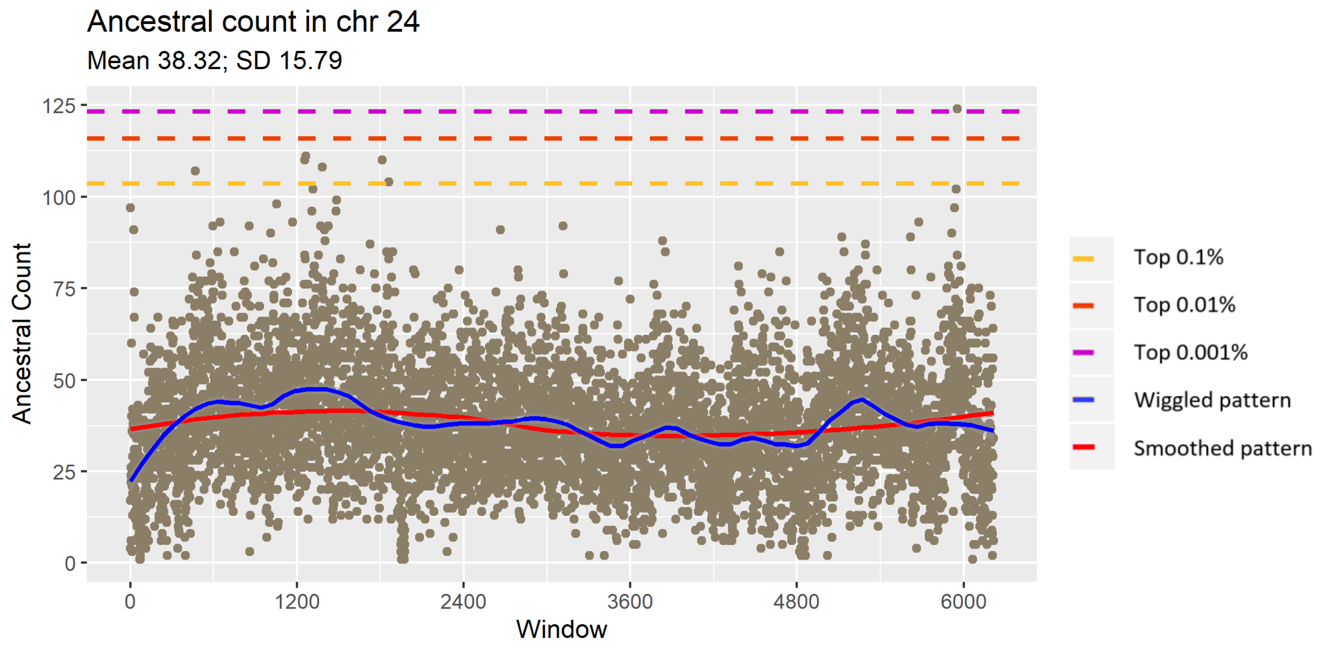
Ancestral count in chr 24
Mean 38.32; SD 15.79
Ancestral Count
Window
0
25
50
75
100
125
0
1200
2400
3600
4800
6000
Top 0.1%
Top 0.01%
Top 0.001%
Wiggled pattern
Smoothed pattern

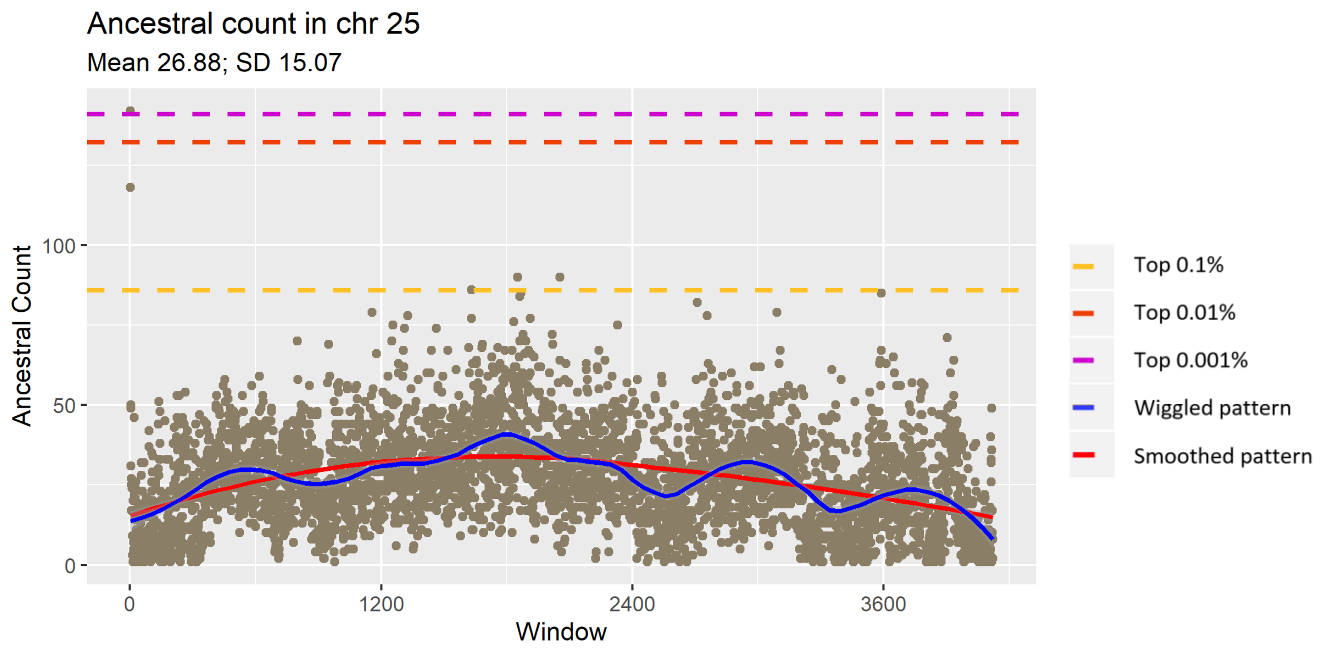
Ancestral count in chr 25
Mean 26.88; SD 15.07
Ancestral Count
100
50
0
0
1200
2400
3600
Window
Top 0.1%
Top 0.01%
Top 0.001%
Wiggled pattern
Smoothed pattern

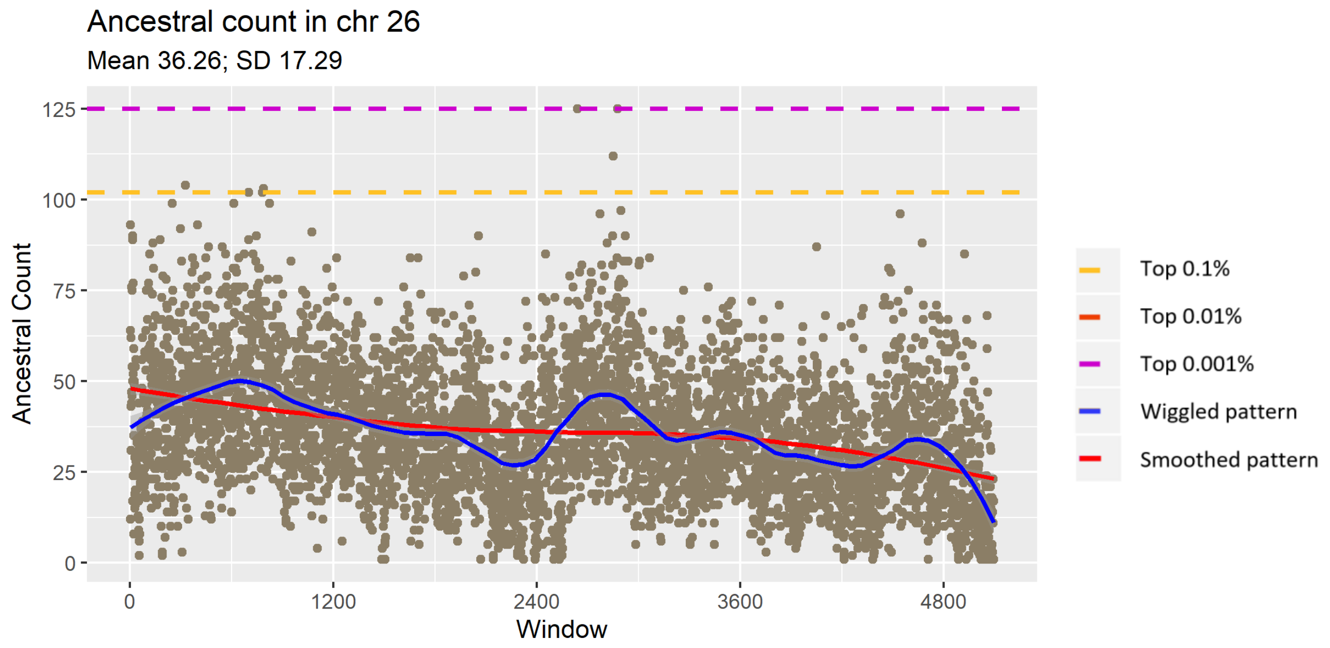
Ancestral count in chr 26
Mean 36.26; SD 17.29
Ancestral Count
125
100
75
50
25
0
0
1200
2400
3600
4800
Window
Top 0.1%
Top 0.01%
Top 0.001%
Wiggled pattern
Smoothed pattern

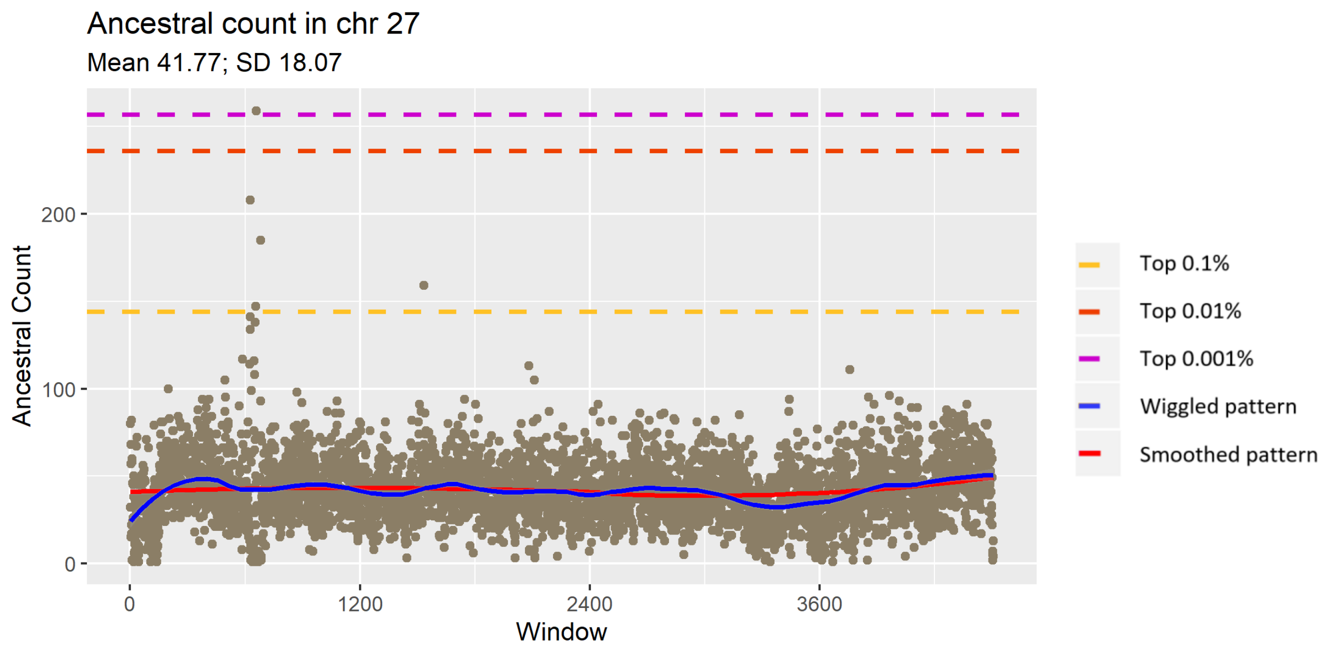
Ancestral count in chr 27
Mean 41.77; SD 18.07
Ancestral Count
200
100
0
0
1200
2400
3600
Window
Top 0.1%
Top 0.01%
Top 0.001%
Wiggled pattern
Smoothed pattern

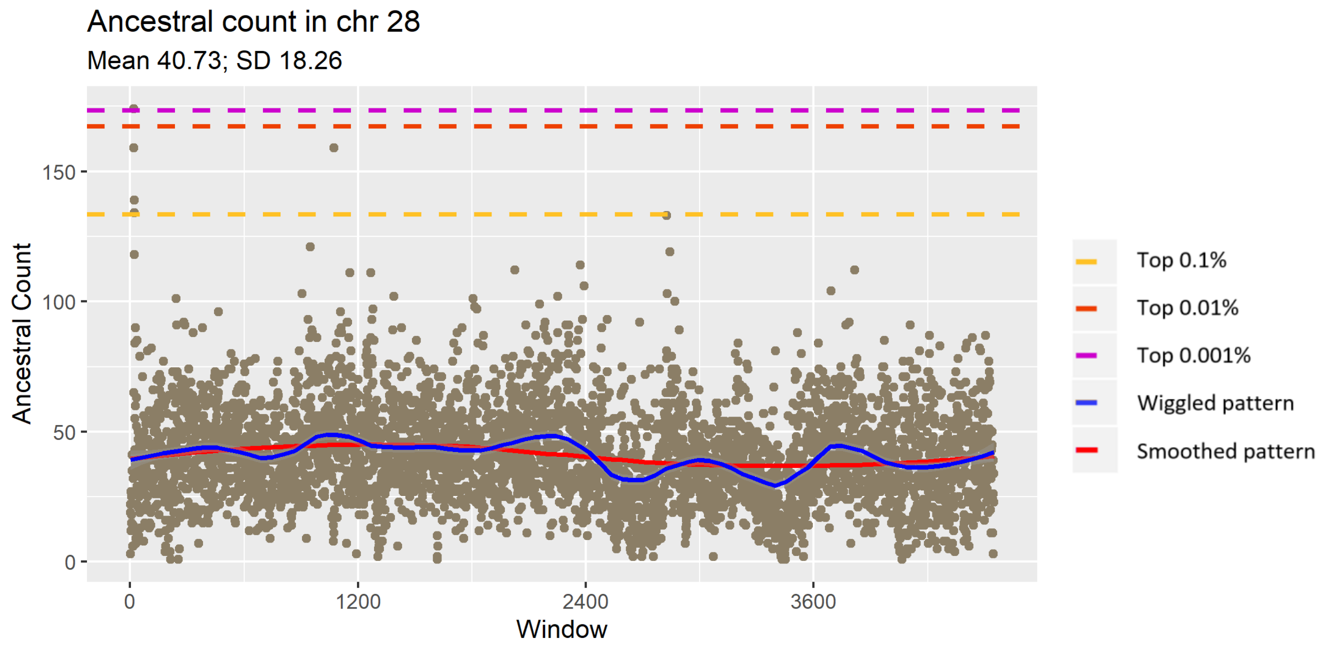
Ancestral count in chr 28
Mean 40.73; SD 18.26
Ancestral Count
150
100
50
0
0
1200
2400
3600
Window
Top 0.1%
Top 0.01%
Top 0.001%
Wiggled pattern
Smoothed pattern

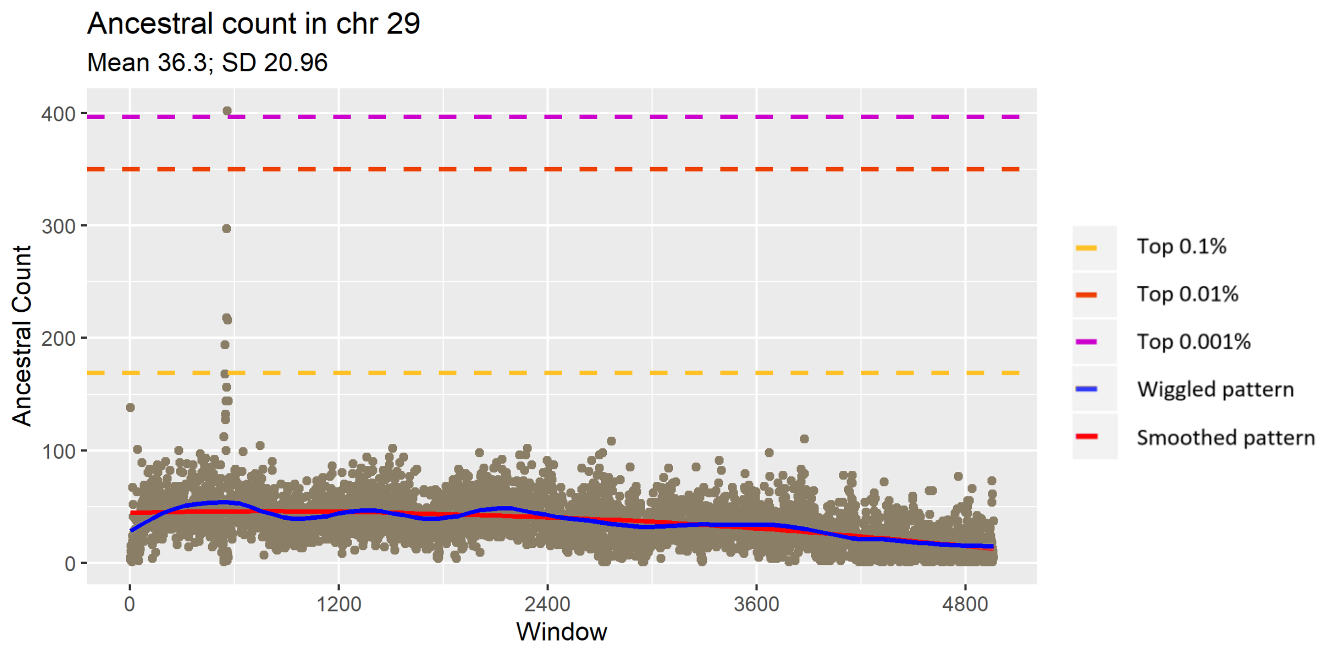
Ancestral count in chr 29
Mean 36.3; SD 20.96
Ancestral Count
0
100
200
300
400
0
1200
2400
3600
4800
Window
Top 0.1%
Top 0.01%
Top 0.001%
Wiggled pattern
Smoothed pattern

Zebu cattle

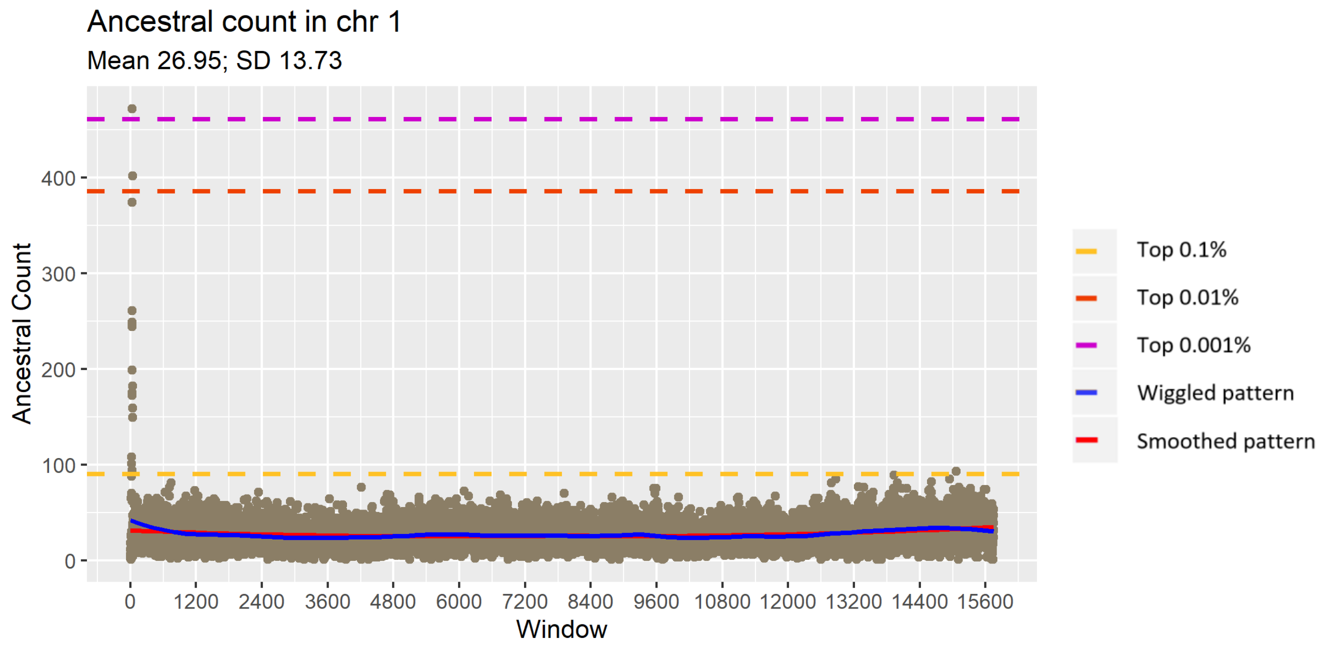
Ancestral count in chr 1
Mean 26.95; SD 13.73
Ancestral Count
0
100
200
300
400
0
1200
2400
3600
4800
6000
7200
8400
9600
10800
12000
13200
14400
15600
Window
Top 0.1%
Top 0.01%
Top 0.001%
Wiggled pattern
Smoothed pattern

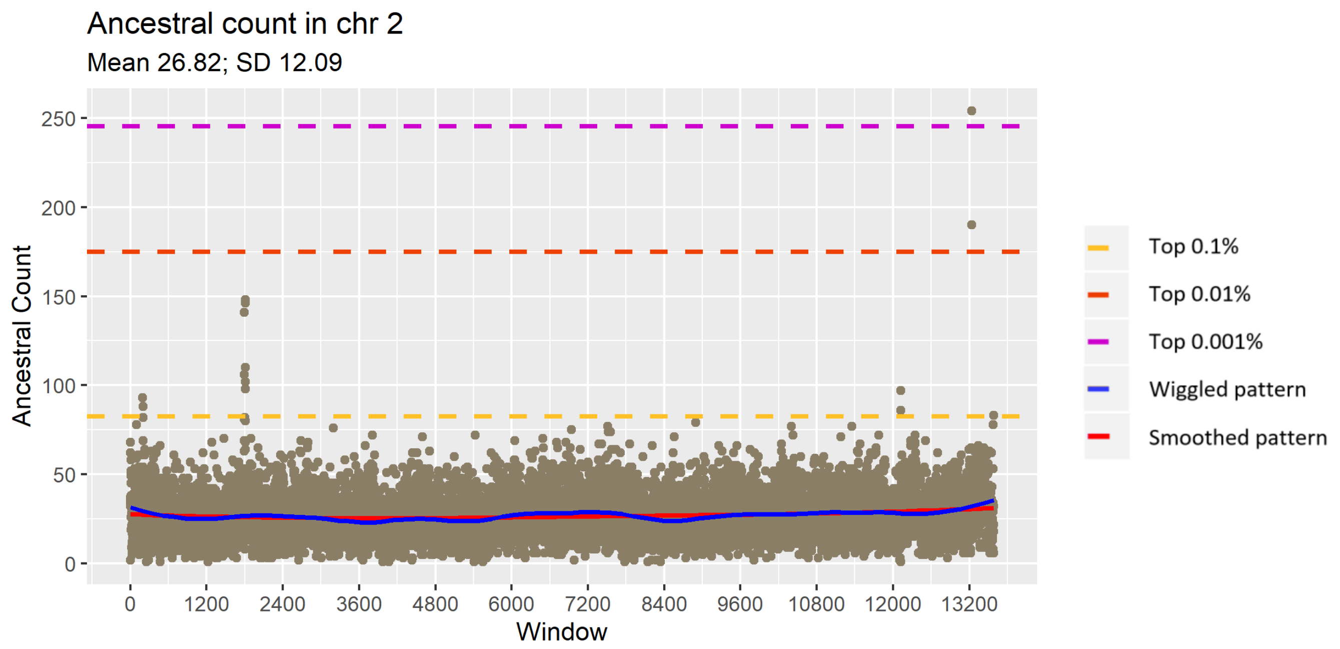
Ancestral count in chr 2
Mean 26.82; SD 12.09
Ancestral Count
250
200
150
100
50
0
0
1200
2400
3600
4800
6000
7200
8400
9600
10800
12000
13200
Window
Top 0.1%
Top 0.01%
Top 0.001%
Wiggled pattern
Smoothed pattern

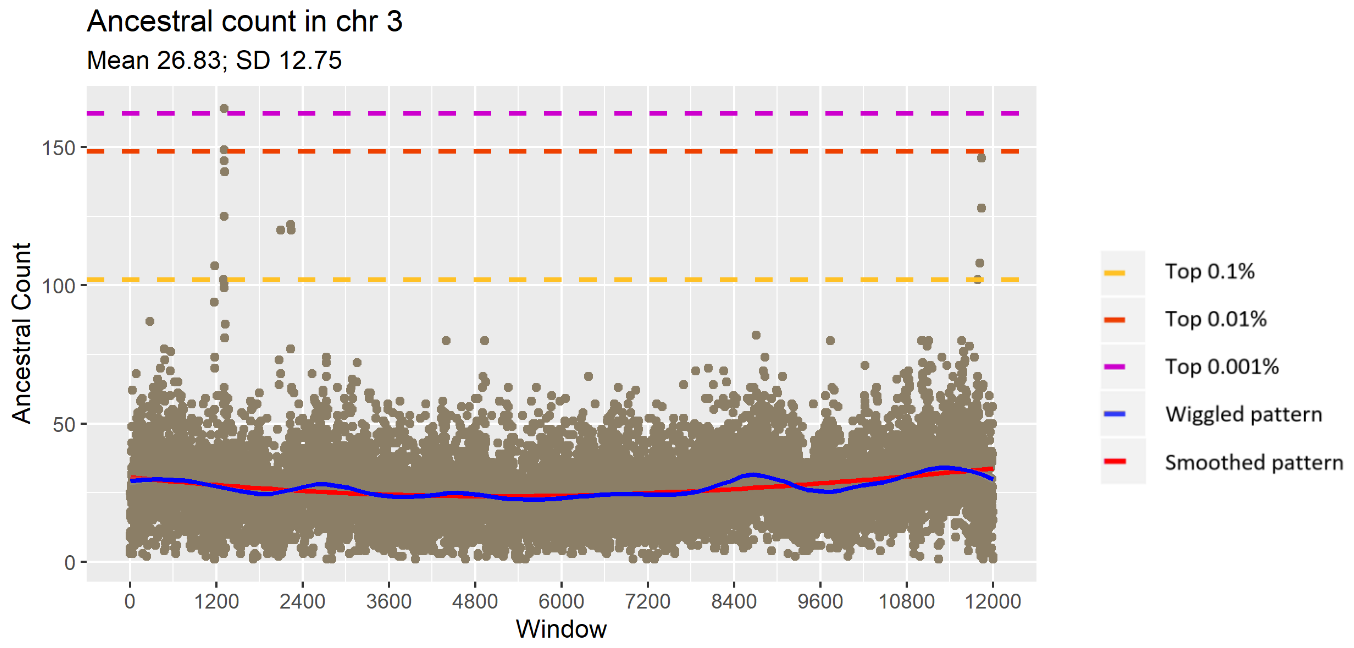
Ancestral count in chr 3
Mean 26.83; SD 12.75
Ancestral Count
150
100
50
0
0
1200
2400
3600
4800
6000
7200
8400
9600
10800
12000
Window
Top 0.1%
Top 0.01%
Top 0.001%
Wiggled pattern
Smoothed pattern

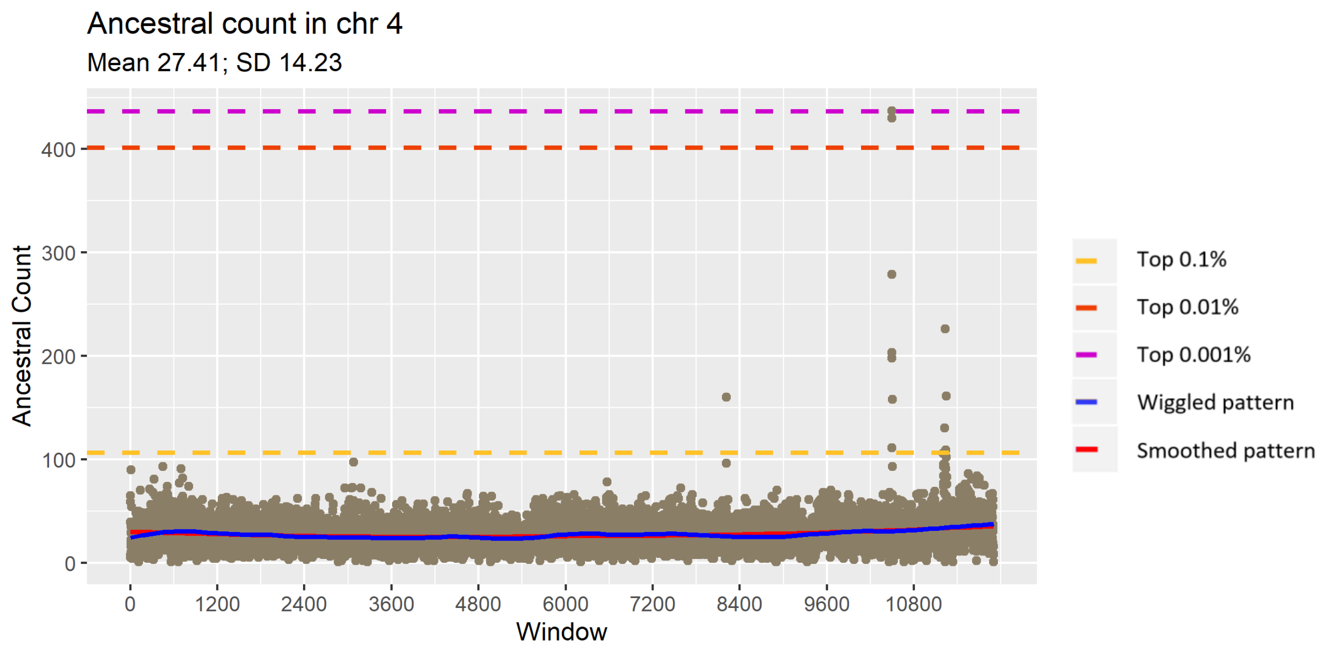

Ancestral count in chr 4
Mean 27.41; SD 14.23
Ancestral Count
Window
0
100
200
300
400
0
1200
2400
3600
4800
6000
7200
8400
9600
10800
Top 0.1%
Top 0.01%
Top 0.001%
Wiggled pattern
Smoothed pattern

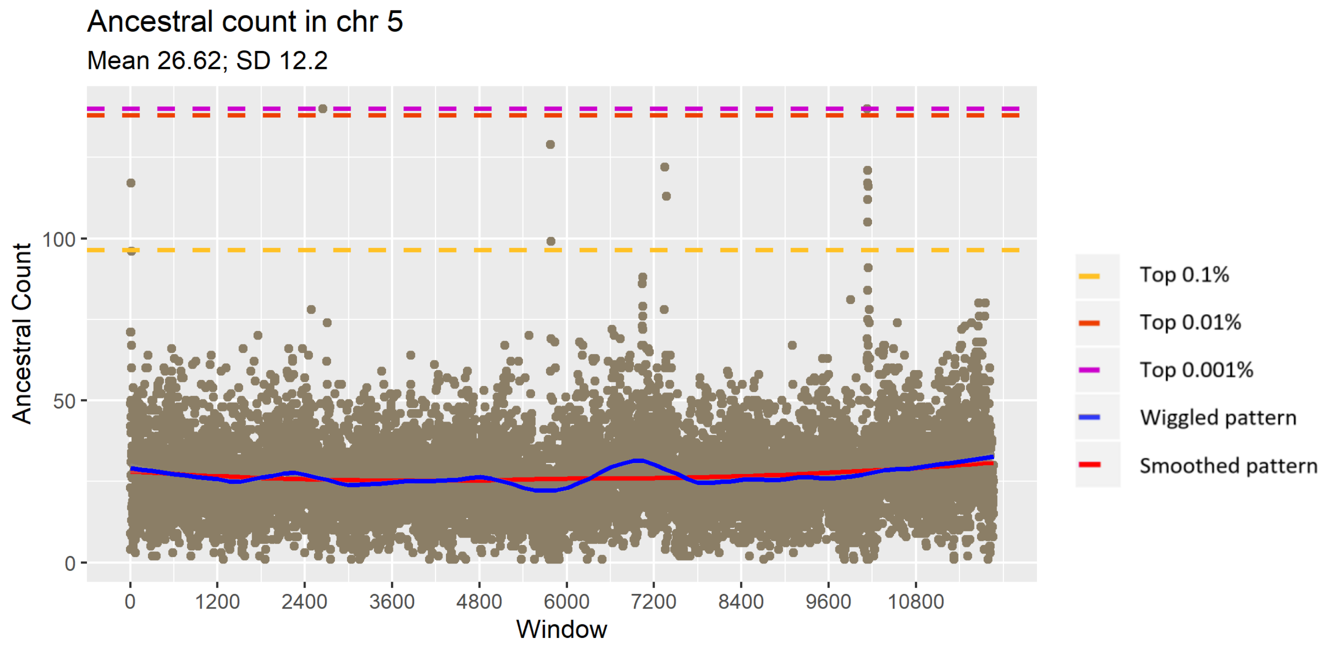

Ancestral count in chr 5
Mean 26.62; SD 12.2
Ancestral Count
Window
0
50
100
0
1200
2400
3600
4800
6000
7200
8400
9600
10800
Top 0.1%
Top 0.01%
Top 0.001%
Wiggled pattern
Smoothed pattern

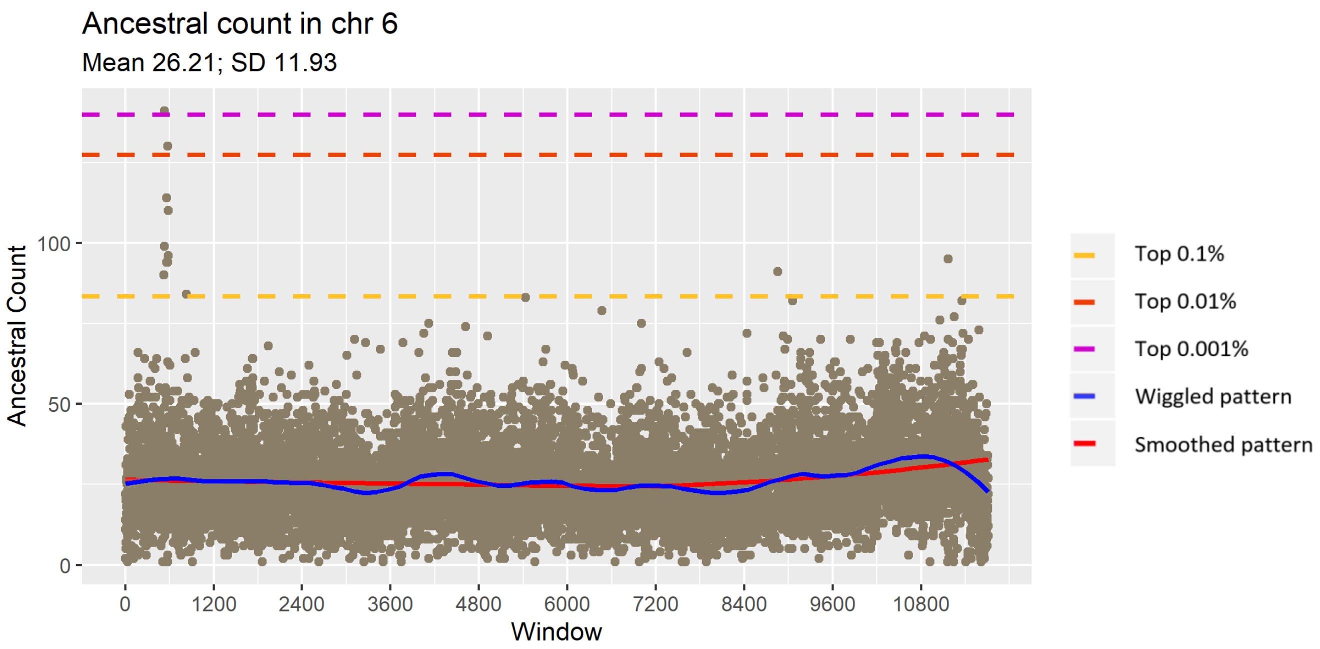
Ancestral count in chr 6
Mean 26.21; SD 11.93
Ancestral Count
Window
Top 0.1%
Top 0.01%
Top 0.001%
Wiggled pattern
Smoothed pattern

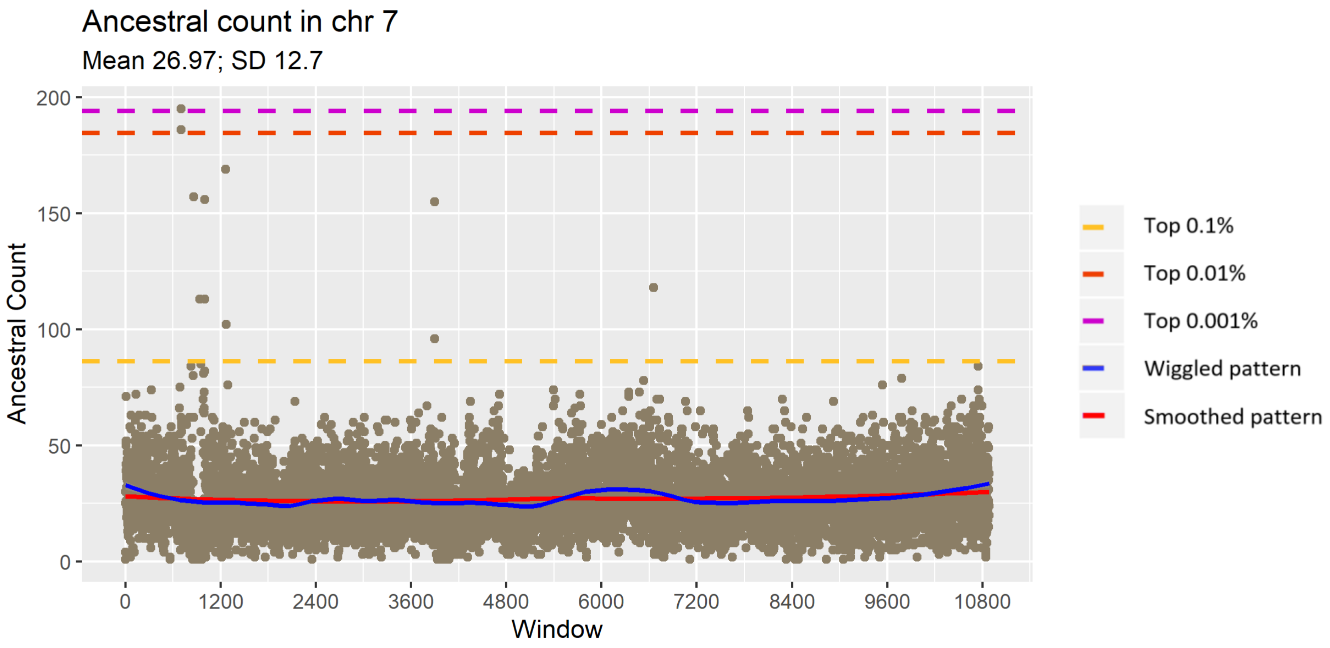
Ancestral count in chr 7
Mean 26.97; SD 12.7
Ancestral Count
Window
Top 0.1%
Top 0.01%
Top 0.001%
Wiggled pattern
Smoothed pattern

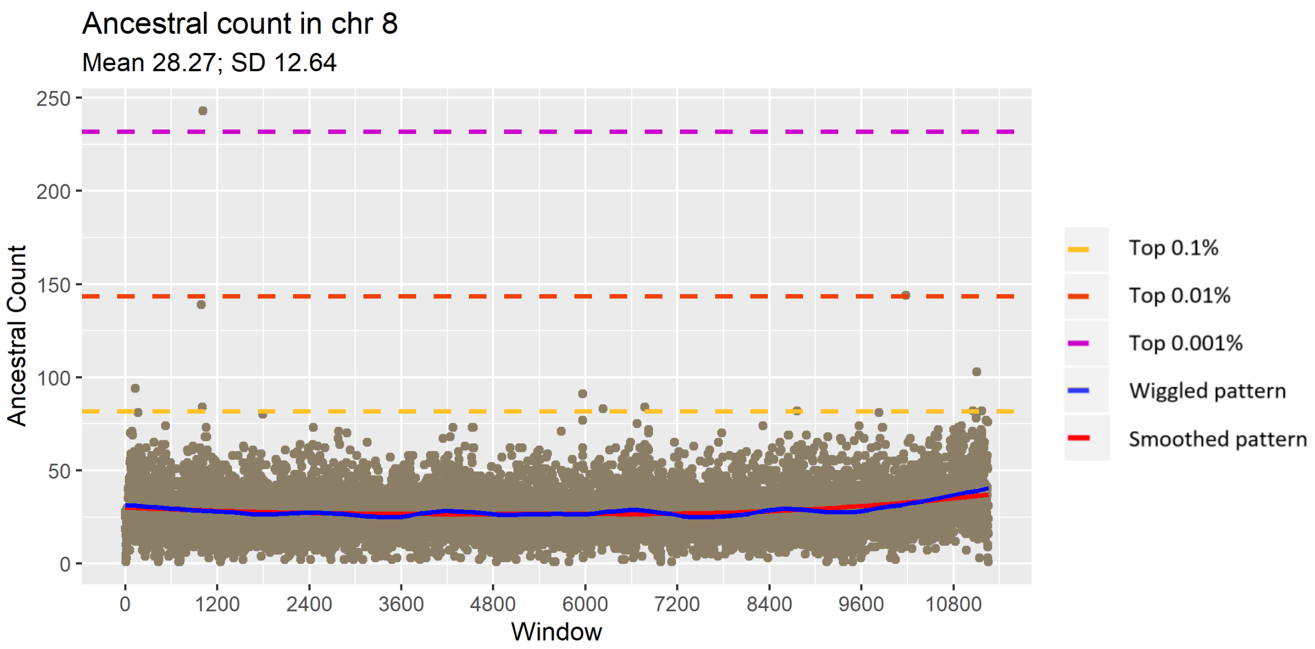
Ancestral count in chr 8
Mean 28.27; SD 12.64
Ancestral Count
Window
Top 0.1%
Top 0.01%
Top 0.001%
Wiggled pattern
Smoothed pattern

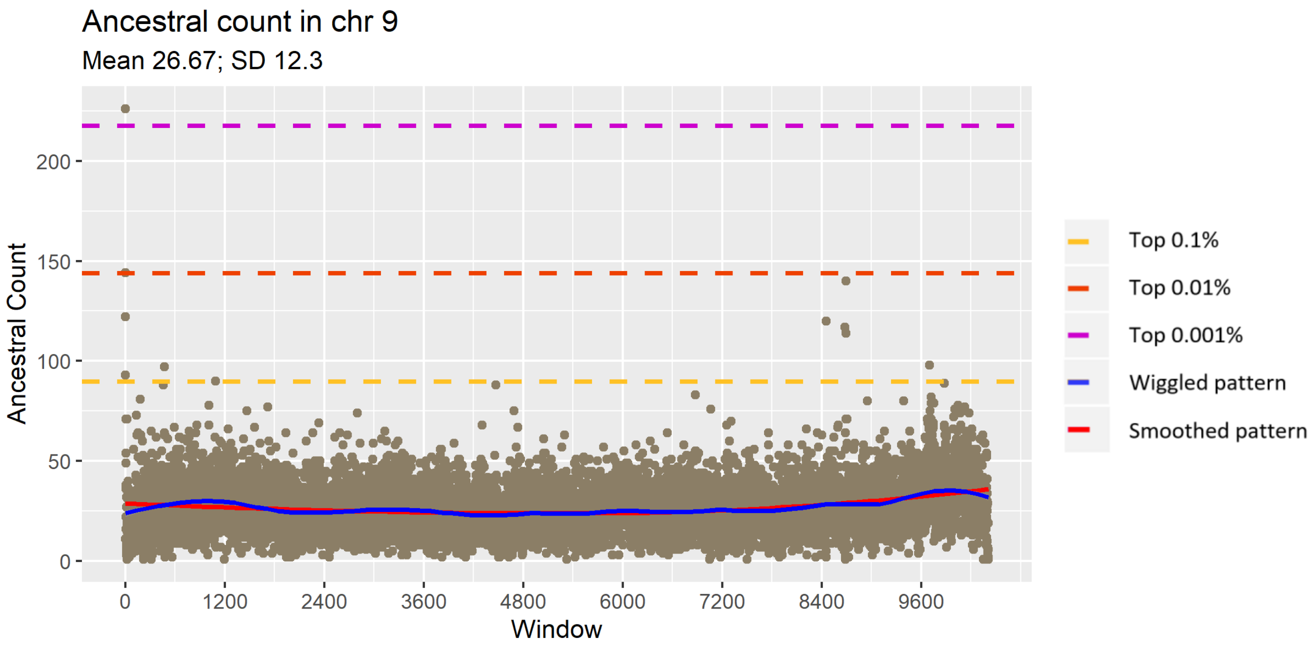
Ancestral count in chr 9
Mean 26.67; SD 12.3
Ancestral Count
Window
Top 0.1%
Top 0.01%
Top 0.001%
Wiggled pattern
Smoothed pattern

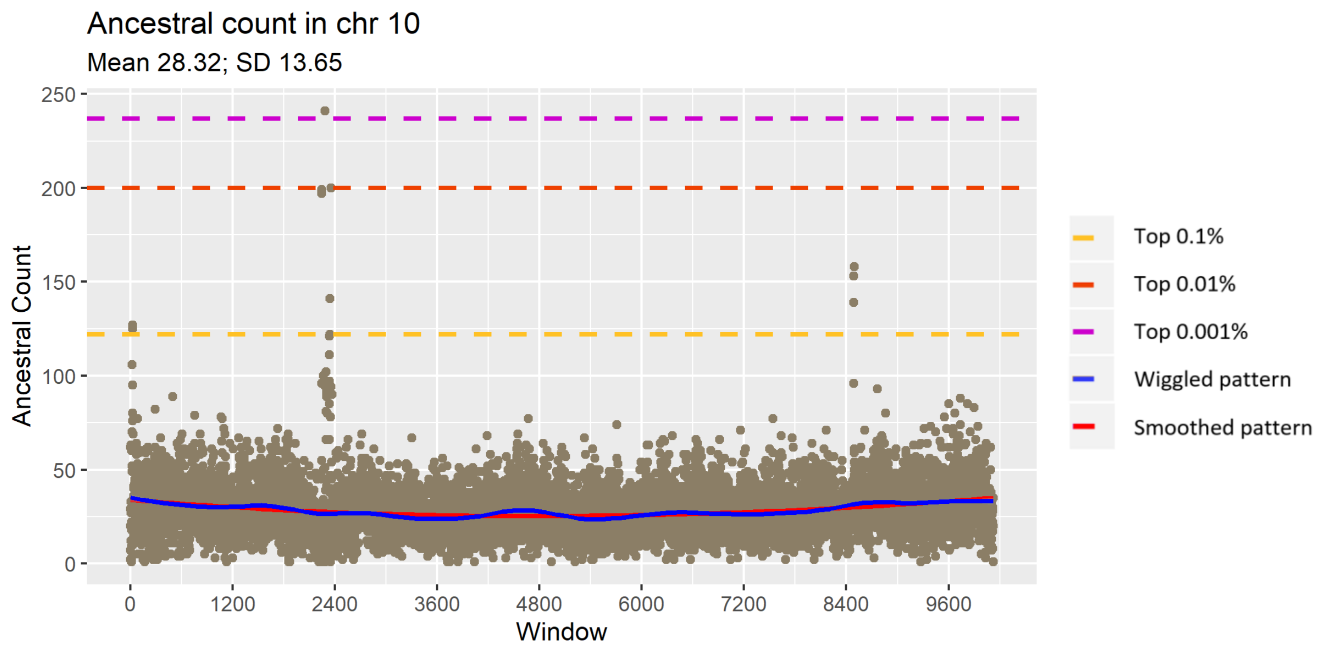
Ancestral count in chr 10
Mean 28.32; SD 13.65
Ancestral Count
Window
0
50
100
150
200
250
1200
2400
3600
4800
6000
7200
8400
9600
Top 0.1%
Top 0.01%
Top 0.001%
Wiggled pattern
Smoothed pattern

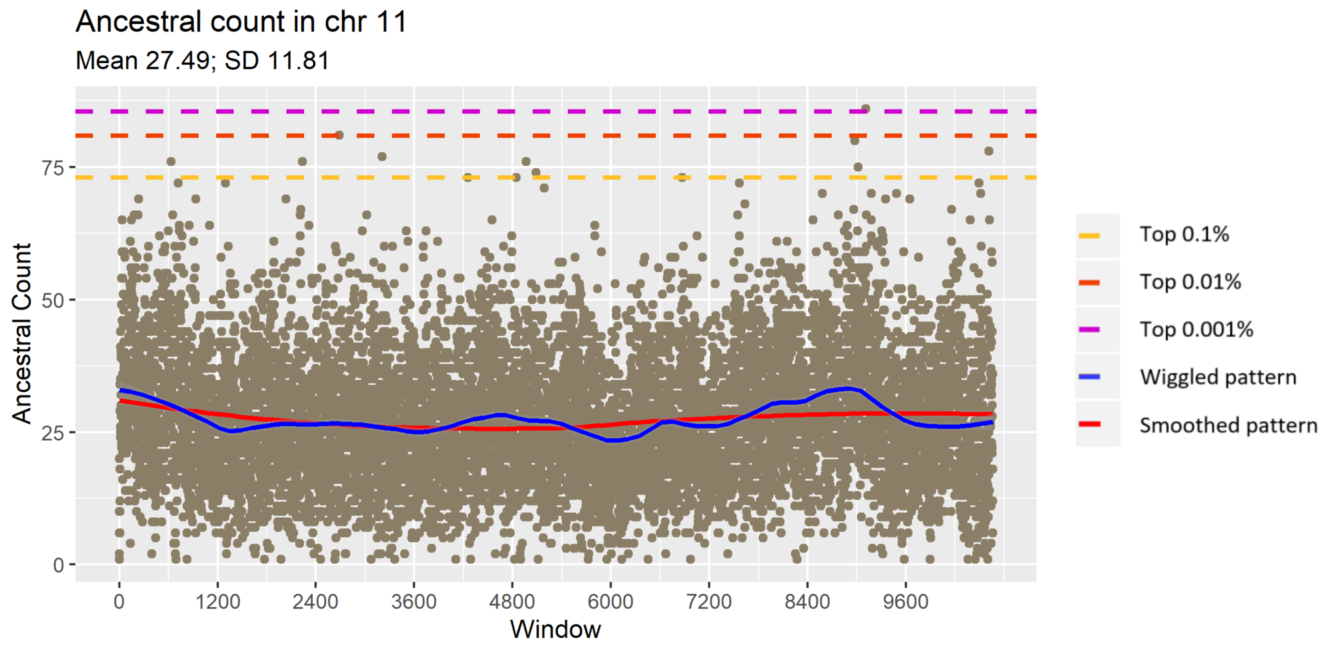
Ancestral count in chr 11
Mean 27.49; SD 11.81
Ancestral Count
Window
0
25
50
75
1200
2400
3600
4800
6000
7200
8400
9600
Top 0.1%
Top 0.01%
Top 0.001%
Wiggled pattern
Smoothed pattern

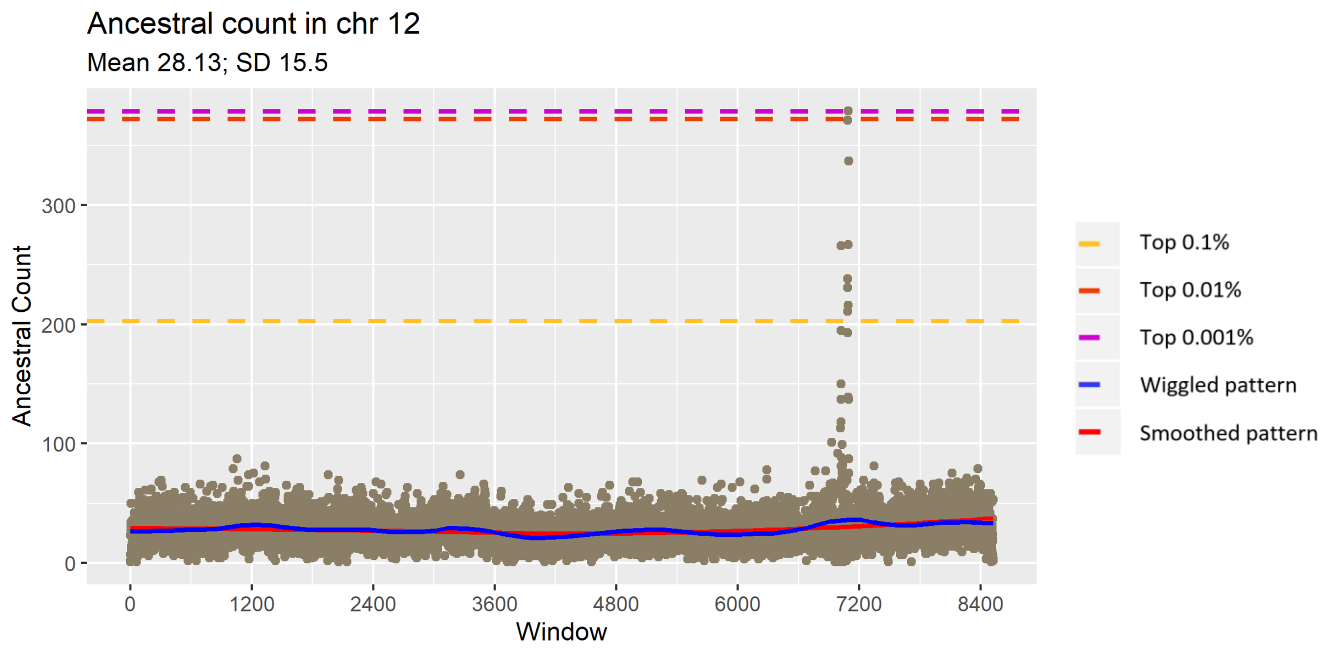
Ancestral count in chr 12
Mean 28.13; SD 15.5
Ancestral Count
300
200
100
0
0
1200
2400
3600
4800
6000
7200
8400
Window
Top 0.1%
Top 0.01%
Top 0.001%
Wiggled pattern
Smoothed pattern

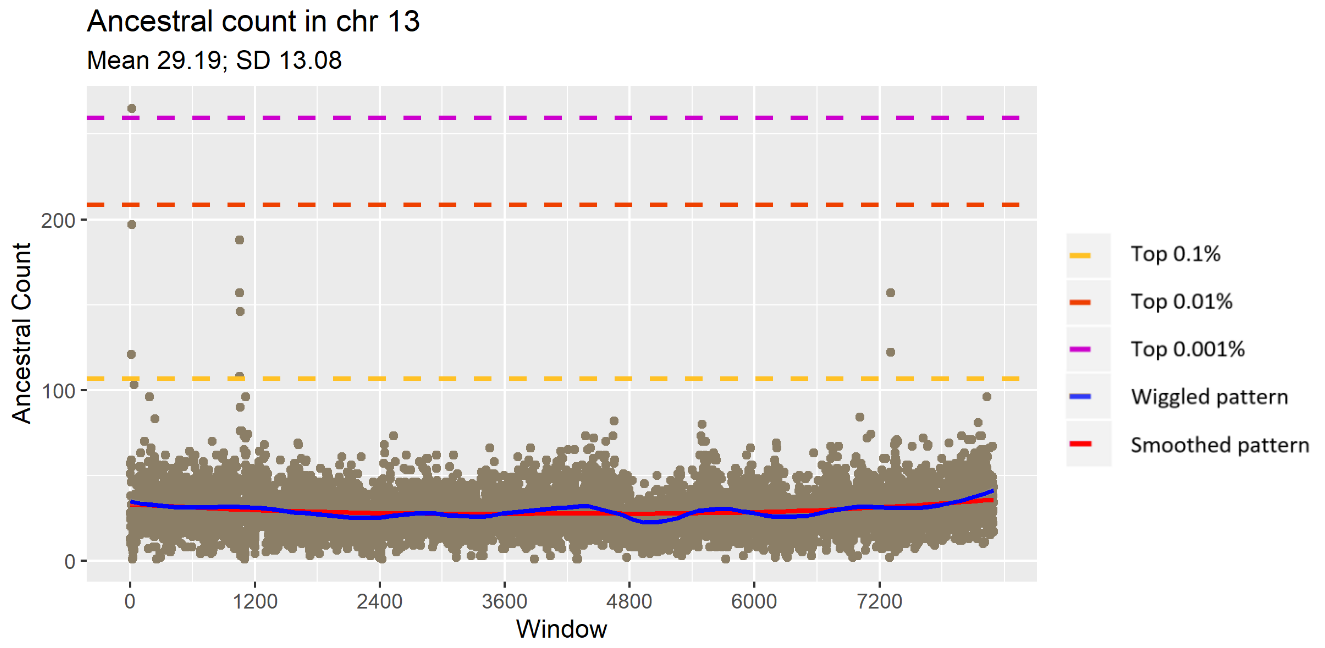
Ancestral count in chr 13
Mean 29.19; SD 13.08
Ancestral Count
200
100
0
0
1200
2400
3600
4800
6000
7200
Window
Top 0.1%
Top 0.01%
Top 0.001%
Wiggled pattern
Smoothed pattern

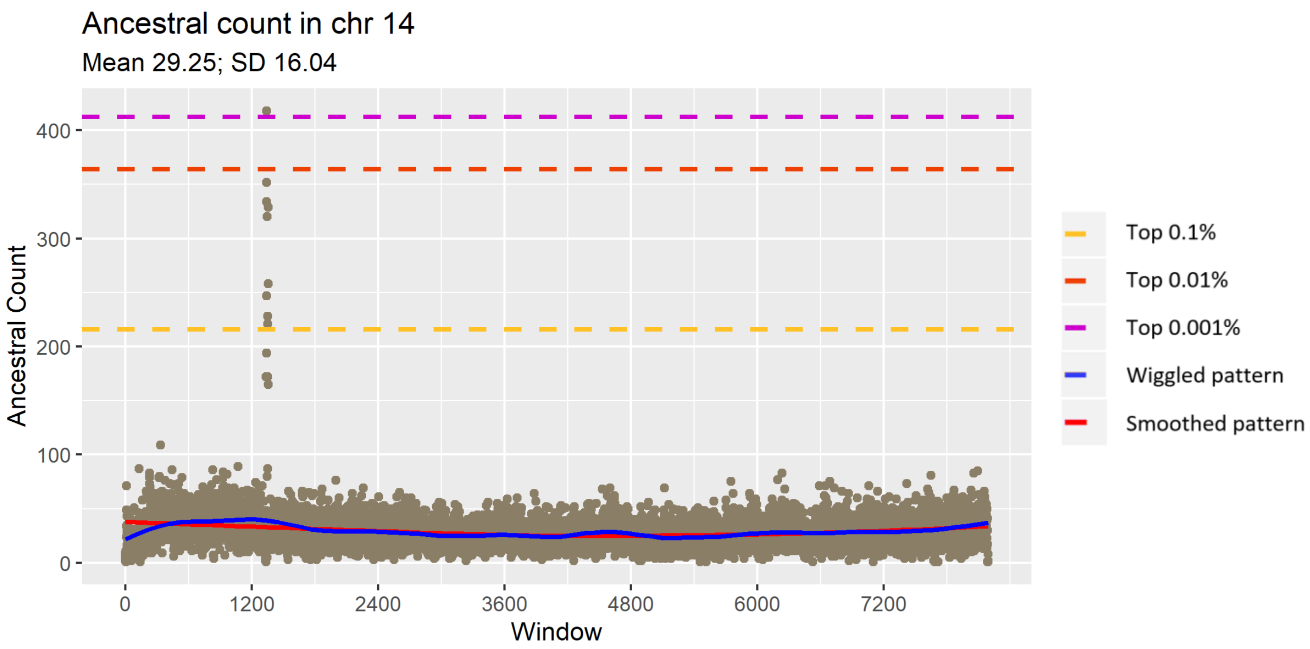
Ancestral count in chr 14
Mean 29.25; SD 16.04
Ancestral Count
400
300
200
100
0
0
1200
2400
3600
4800
6000
7200
Window
Top 0.1%
Top 0.01%
Top 0.001%
Wiggled pattern
Smoothed pattern

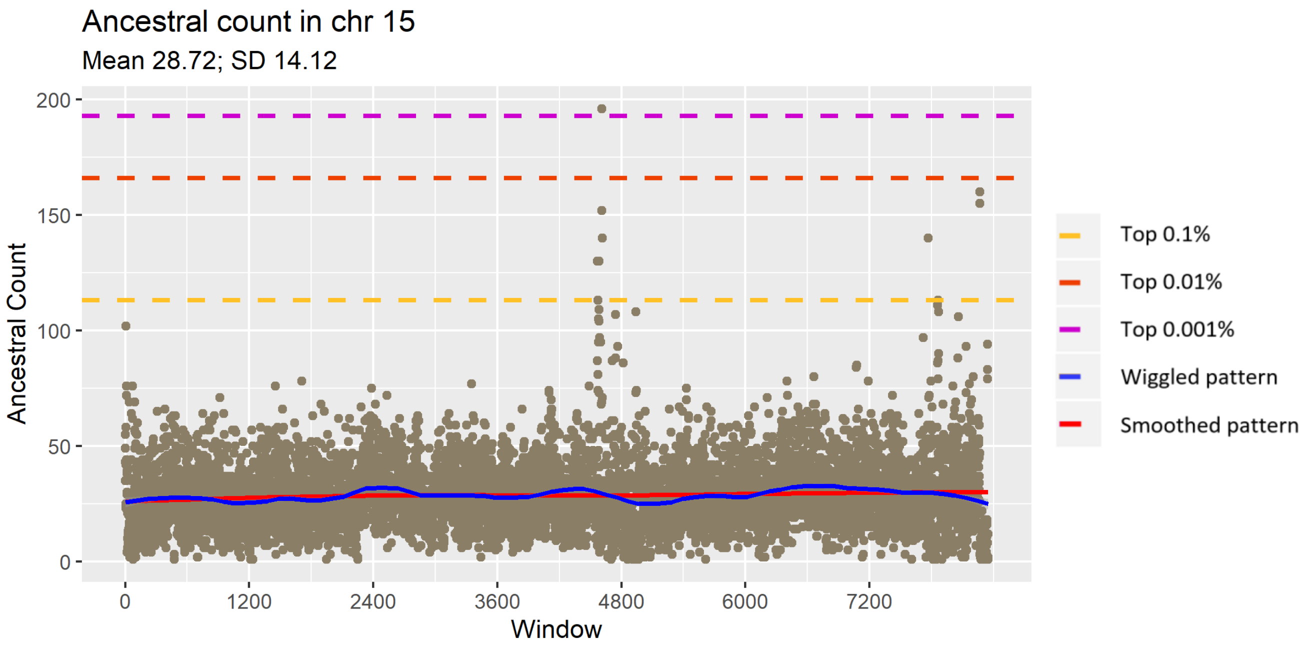
Ancestral count in chr 15
Mean 28.72; SD 14.12
Ancestral Count
200
150
100
50
0
0
1200
2400
3600
4800
6000
7200
Window
Top 0.1%
Top 0.01%
Top 0.001%
Wiggled pattern
Smoothed pattern

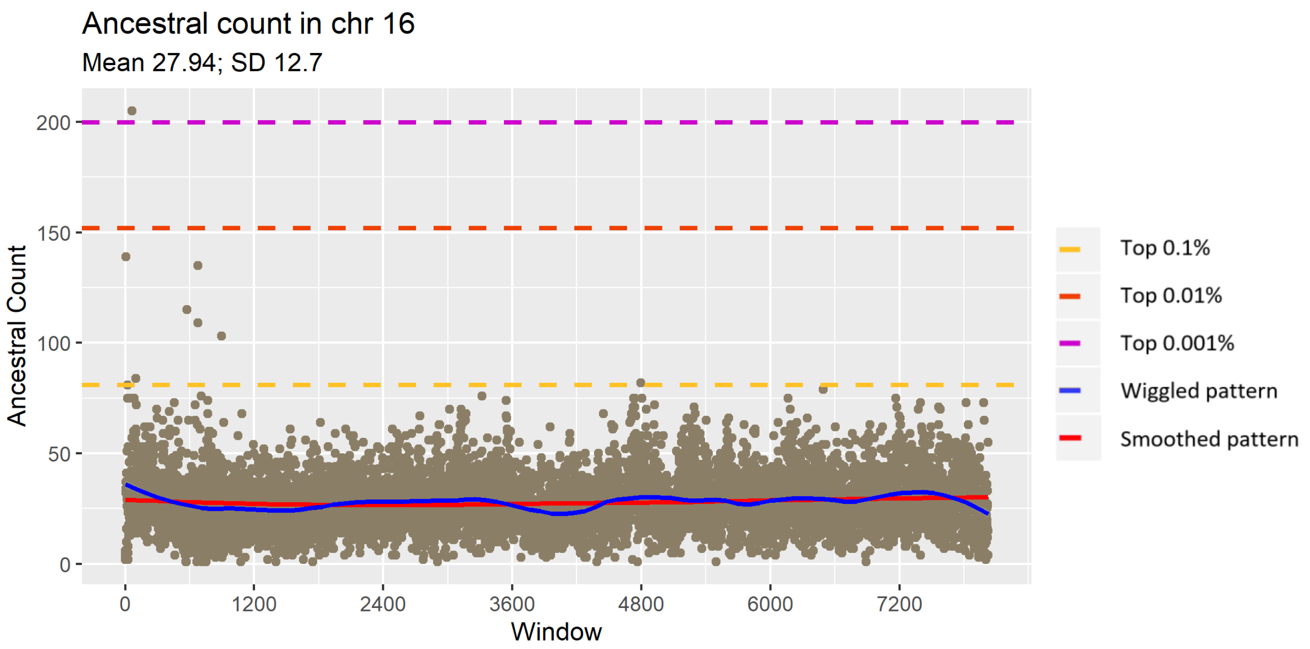
Ancestral count in chr 16
Mean 27.94; SD 12.7
Ancestral Count
Window
0
50
100
150
200
0
1200
2400
3600
4800
6000
7200
Top 0.1%
Top 0.01%
Top 0.001%
Wiggled pattern
Smoothed pattern

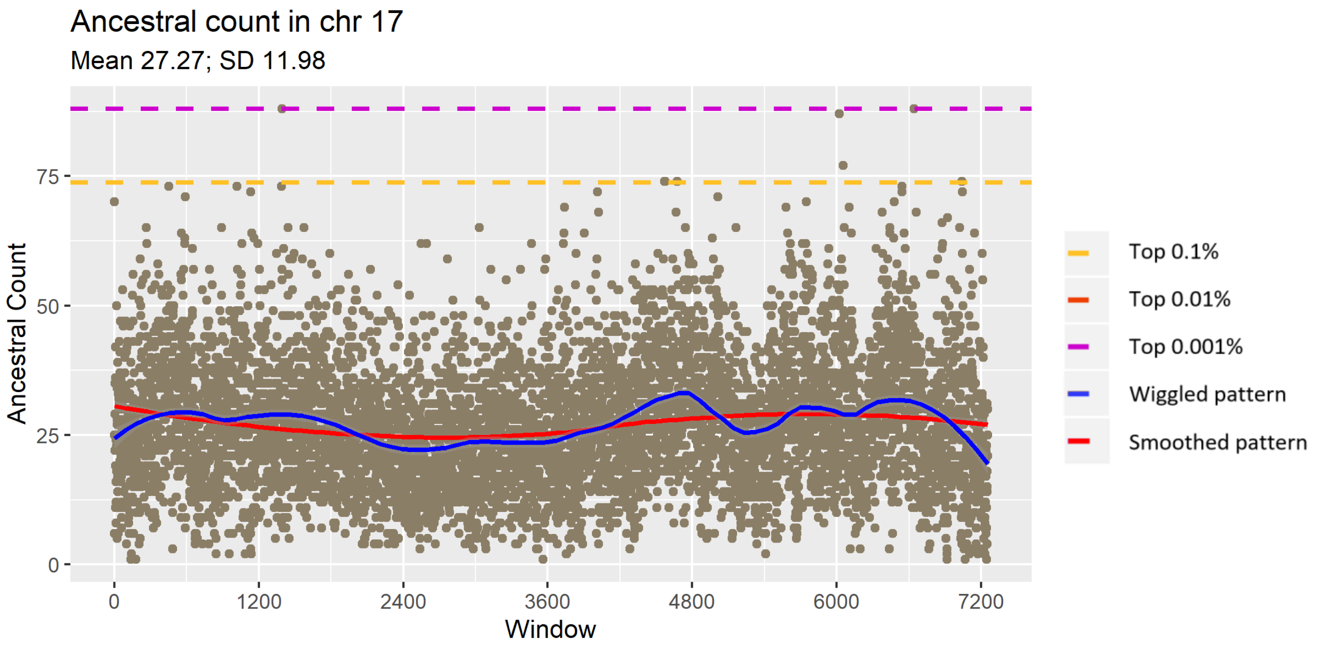
Ancestral count in chr 17
Mean 27.27; SD 11.98
Ancestral Count
Window
0
25
50
75
0
1200
2400
3600
4800
6000
7200
Top 0.1%
Top 0.01%
Top 0.001%
Wiggled pattern
Smoothed pattern

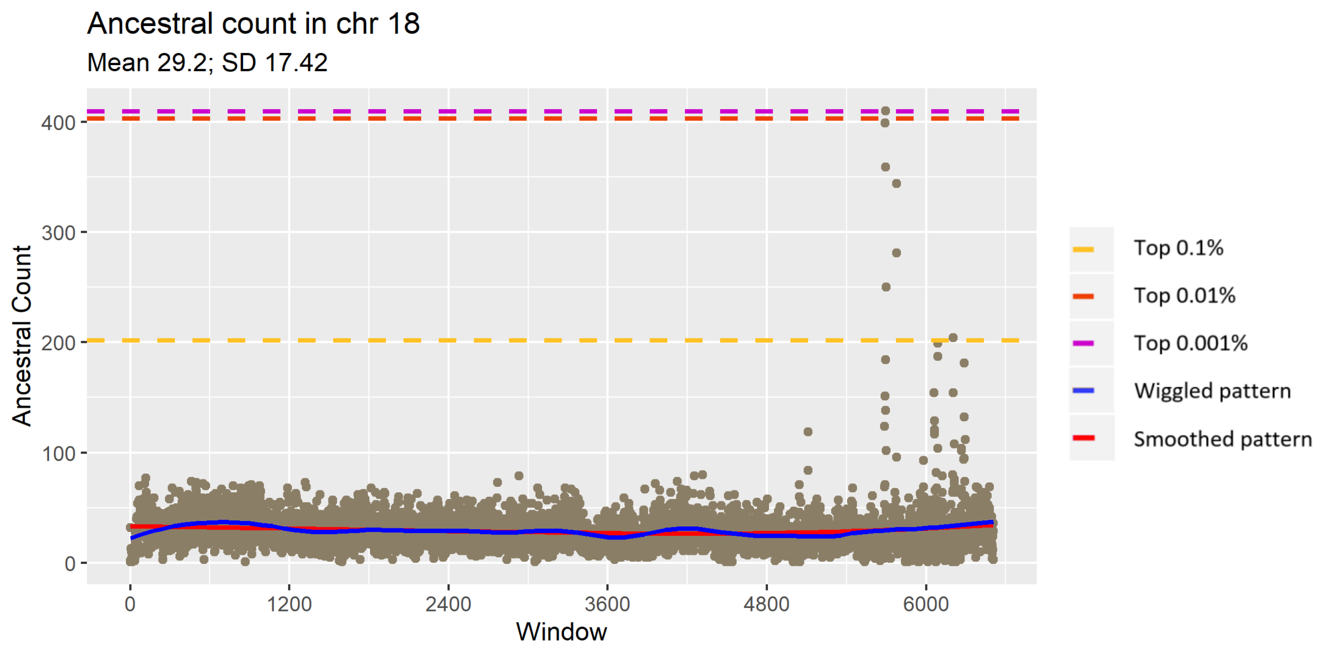

Ancestral count in chr 18
Mean 29.2; SD 17.42
Ancestral Count
400
300
200
100
0
0
1200
2400
3600
4800
6000
Window
Top 0.1%
Top 0.01%
Top 0.001%
Wiggled pattern
Smoothed pattern

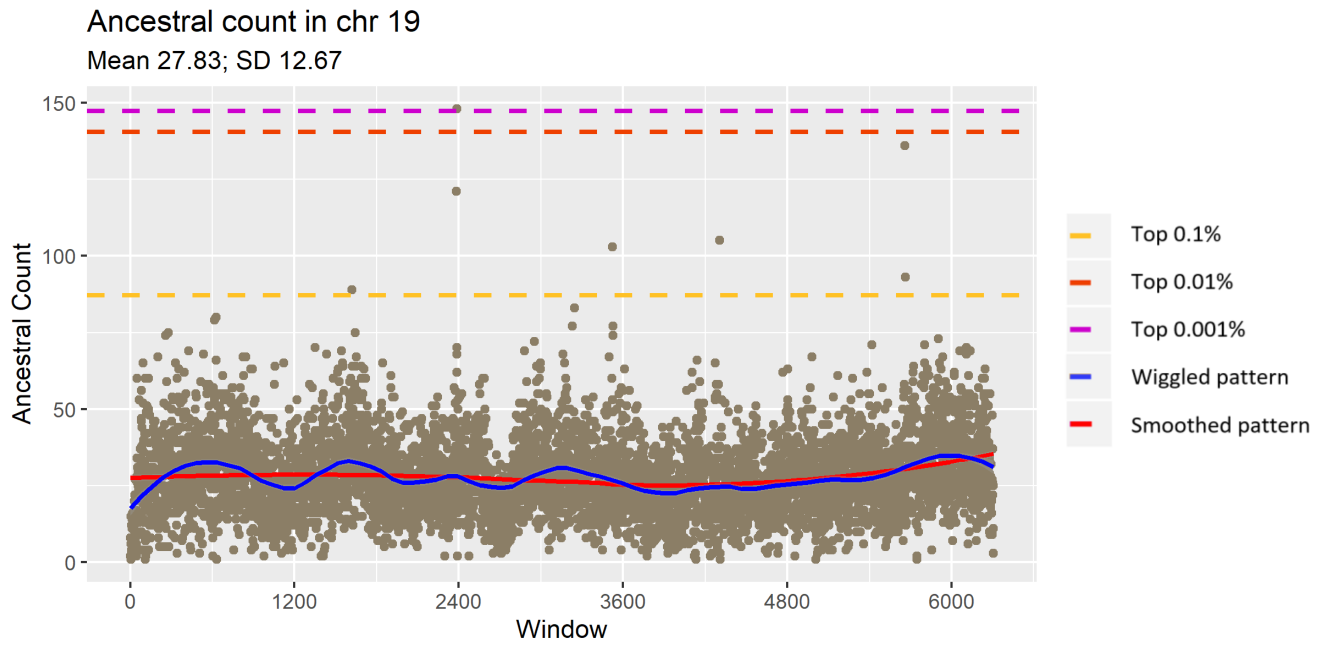

Ancestral count in chr 19
Mean 27.83; SD 12.67
Ancestral Count
150
100
50
0
0
1200
2400
3600
4800
6000
Window
Top 0.1%
Top 0.01%
Top 0.001%
Wiggled pattern
Smoothed pattern

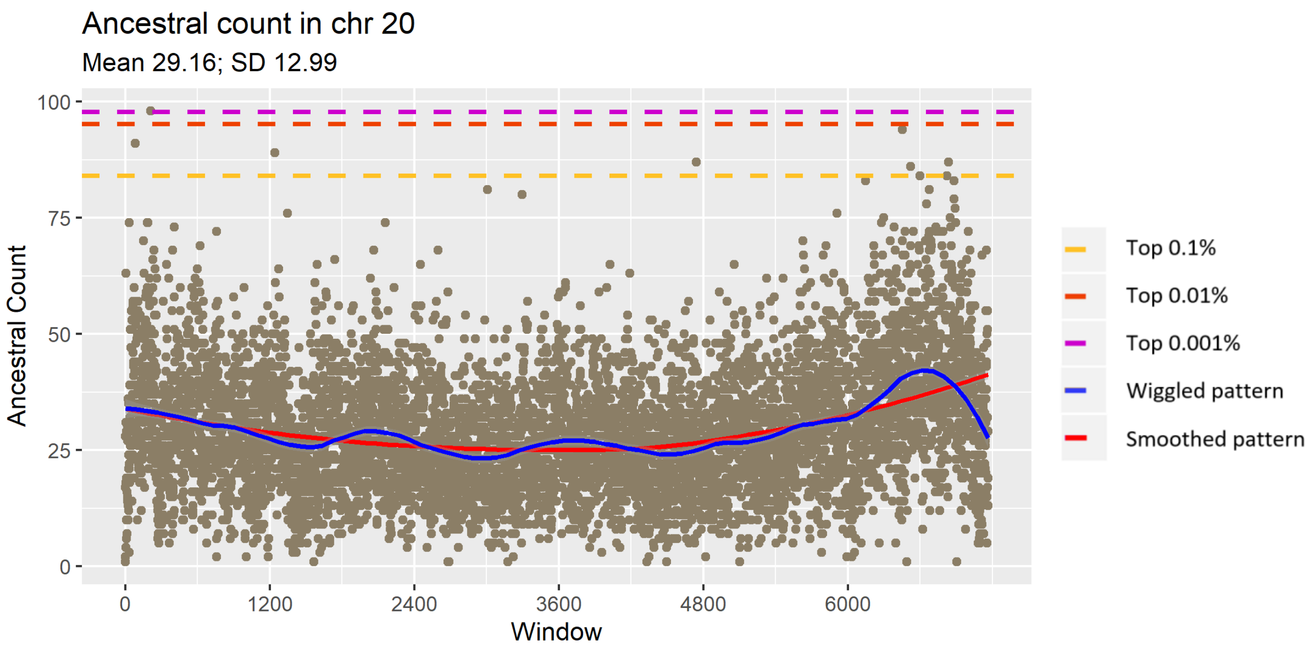
Ancestral count in chr 20
Mean 29.16; SD 12.99
Ancestral Count
100
75
50
25
0
0
1200
2400
3600
4800
6000
Window
Top 0.1%
Top 0.01%
Top 0.001%
Wiggled pattern
Smoothed pattern

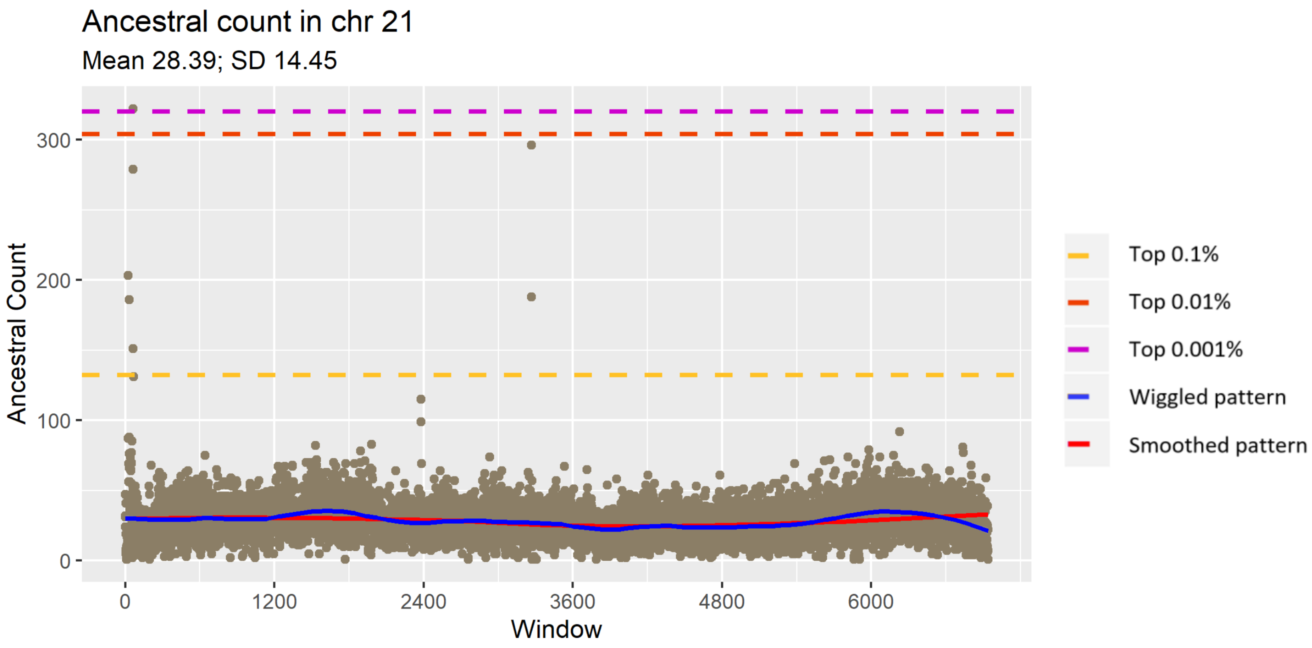
Ancestral count in chr 21
Mean 28.39; SD 14.45
Ancestral Count
300
200
100
0
0
1200
2400
3600
4800
6000
Window
Top 0.1%
Top 0.01%
Top 0.001%
Wiggled pattern
Smoothed pattern

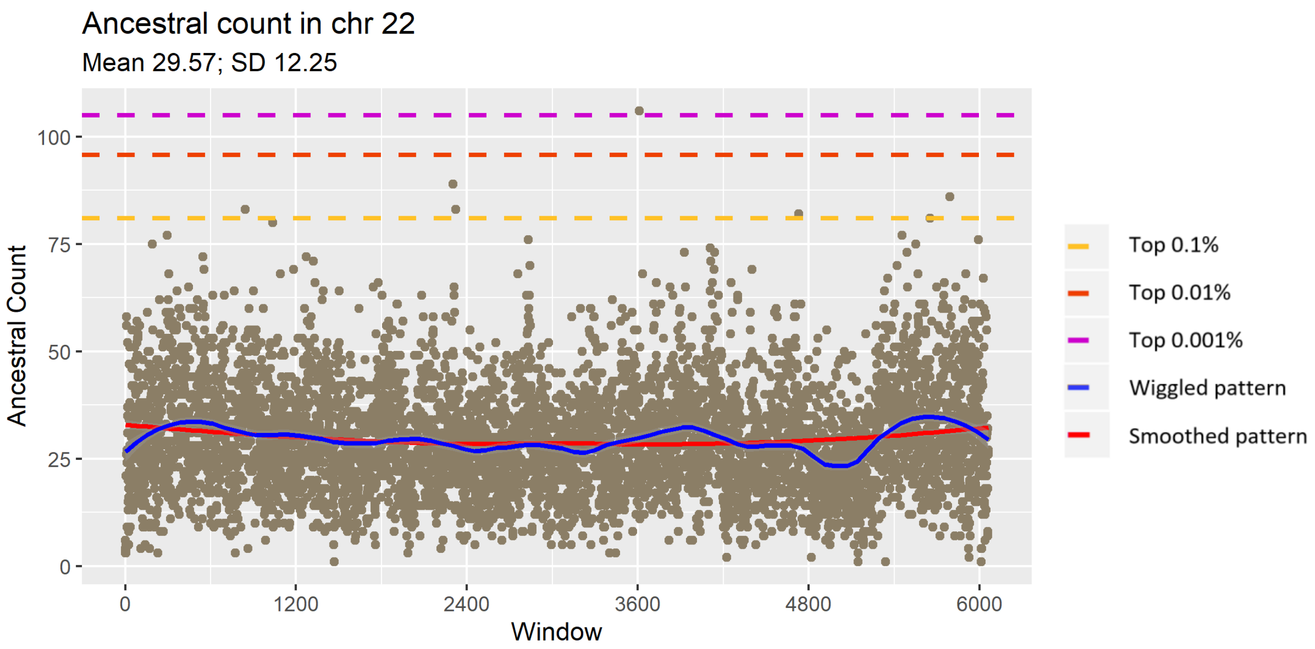
Ancestral count in chr 22
Mean 29.57; SD 12.25
Ancestral Count
Window
0
25
50
75
100
0
1200
2400
3600
4800
6000
Top 0.1%
Top 0.01%
Top 0.001%
Wiggled pattern
Smoothed pattern

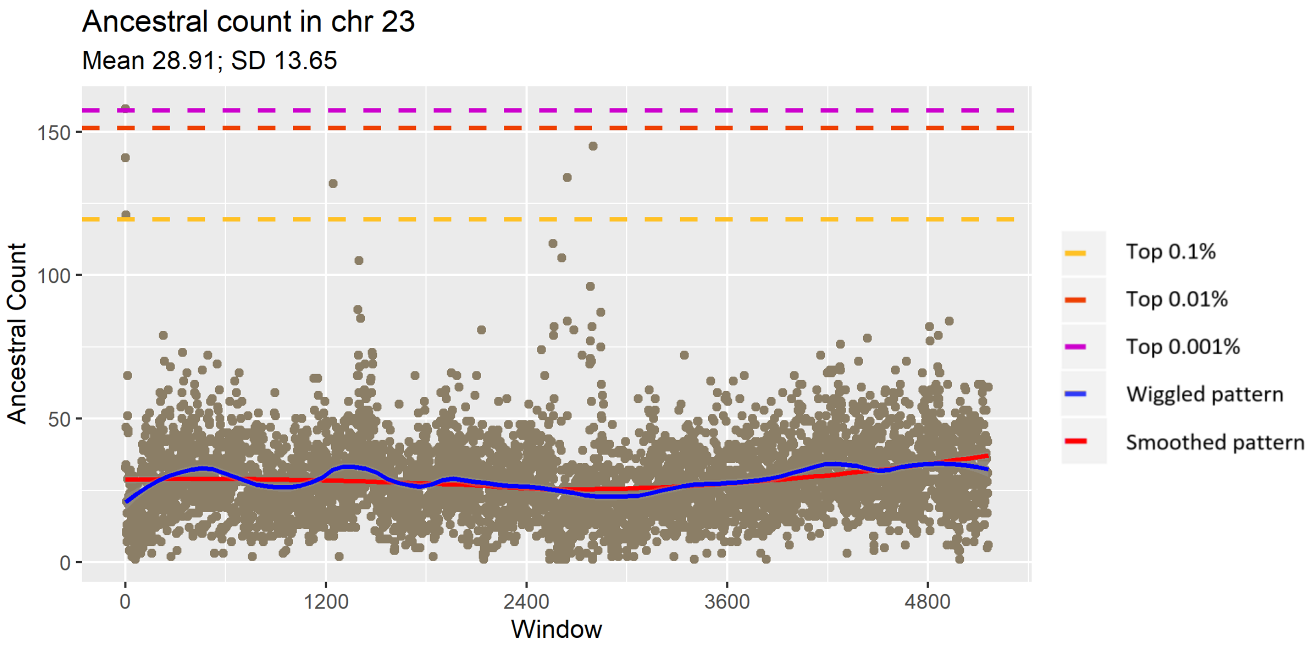
Ancestral count in chr 23
Mean 28.91; SD 13.65
Ancestral Count
Window
0
50
100
150
0
1200
2400
3600
4800
Top 0.1%
Top 0.01%
Top 0.001%
Wiggled pattern
Smoothed pattern

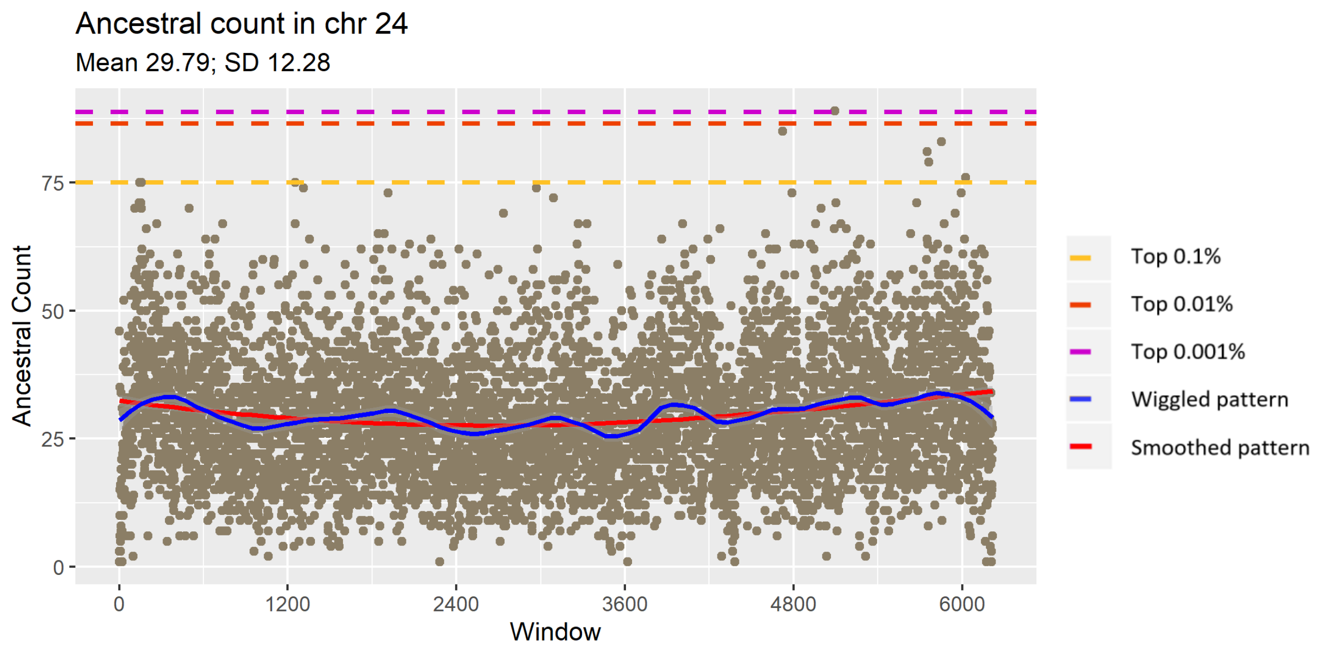

Ancestral count in chr 24
Mean 29.79; SD 12.28
Ancestral Count
0
25
50
75
0
1200
2400
3600
4800
6000
Window
Top 0.1%
Top 0.01%
Top 0.001%
Wiggled pattern
Smoothed pattern

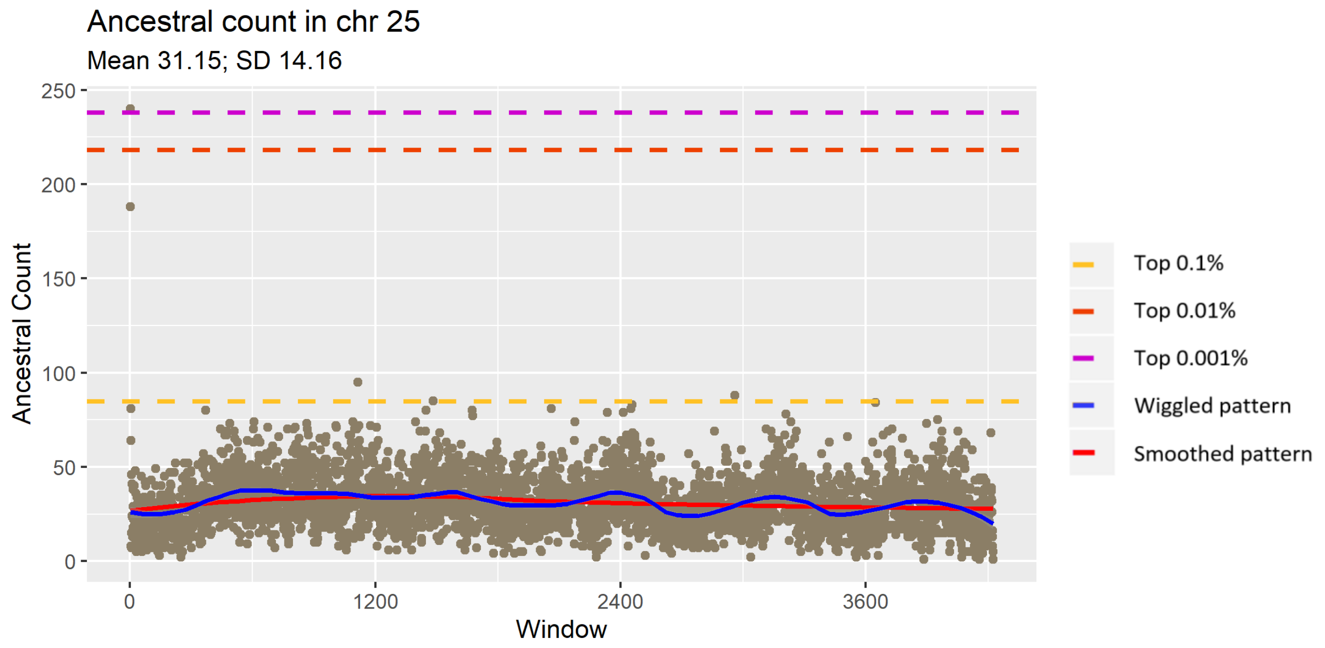

Ancestral count in chr 25
Mean 31.15; SD 14.16
Ancestral Count
0
50
100
150
200
250
0
1200
2400
3600
Window
Top 0.1%
Top 0.01%
Top 0.001%
Wiggled pattern
Smoothed pattern

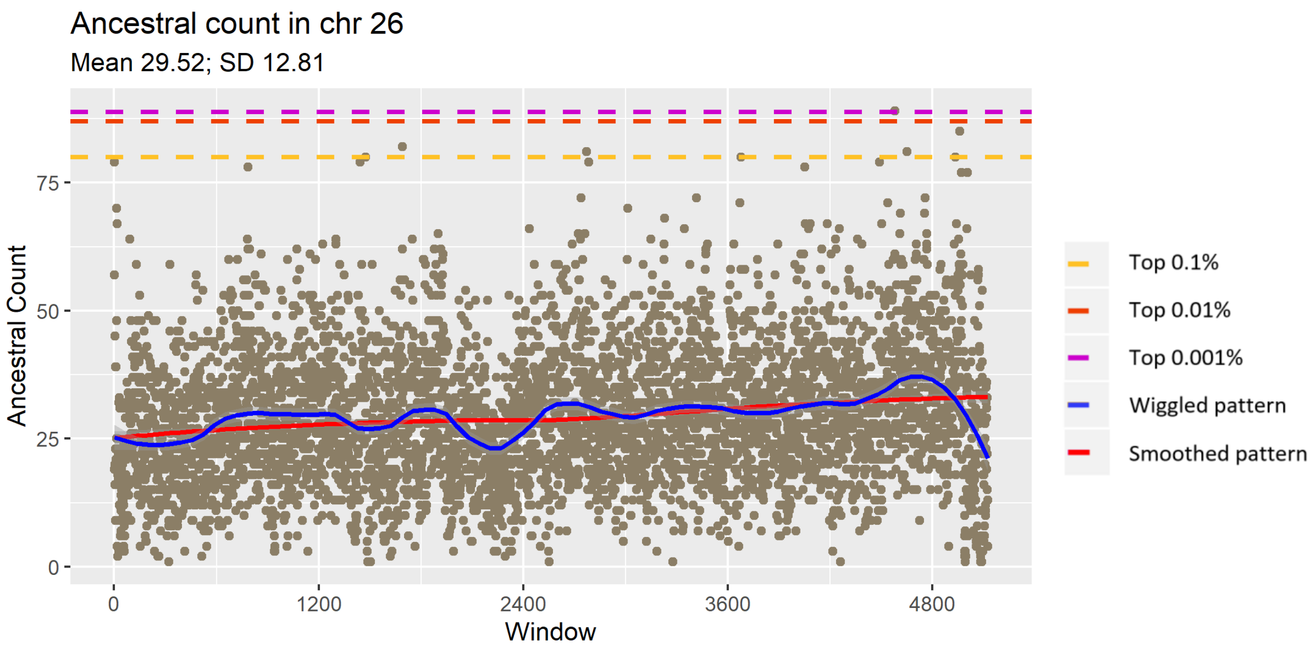
Ancestral count in chr 26
Mean 29.52; SD 12.81
Ancestral Count
Window
0
25
50
75
0
1200
2400
3600
4800
Top 0.1%
Top 0.01%
Top 0.001%
Wiggled pattern
Smoothed pattern

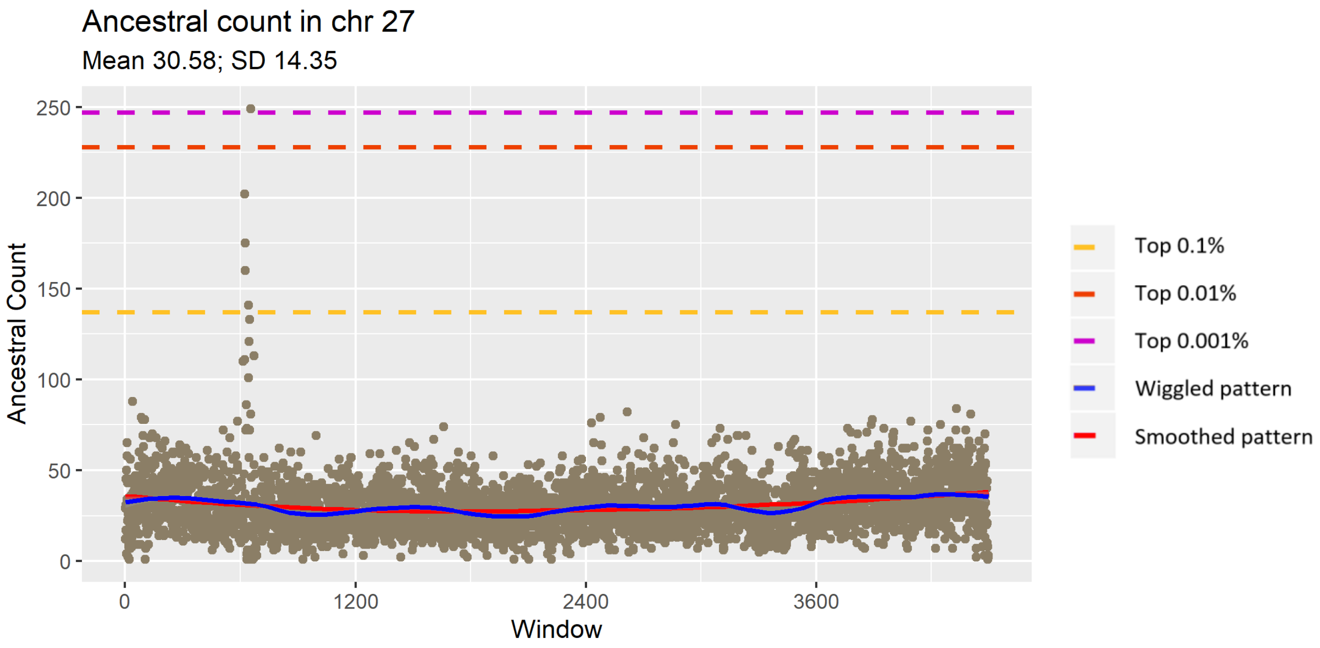
Ancestral count in chr 27
Mean 30.58; SD 14.35
Ancestral Count
Window
0
50
100
150
200
250
0
1200
2400
3600
Top 0.1%
Top 0.01%
Top 0.001%
Wiggled pattern
Smoothed pattern

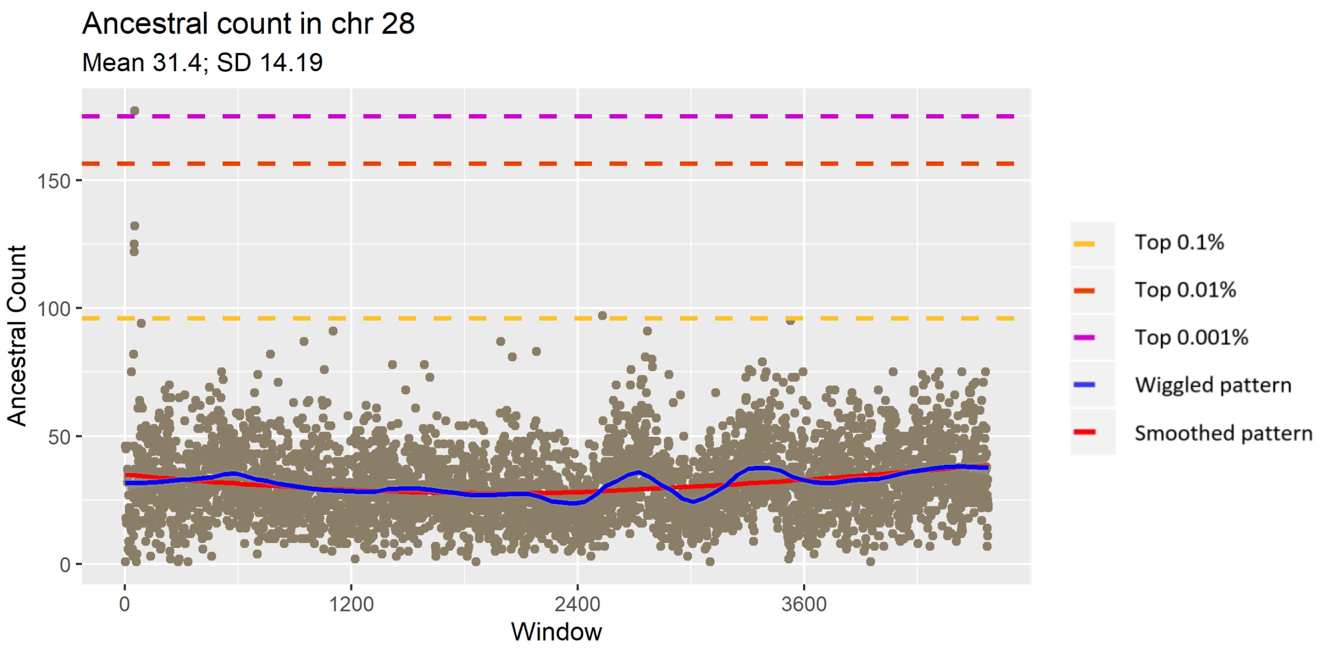
Ancestral count in chr 28
Mean 31.4; SD 14.19
Ancestral Count
Window
0
50
100
150
0
1200
2400
3600
Top 0.1%
Top 0.01%
Top 0.001%
Wiggled pattern
Smoothed pattern

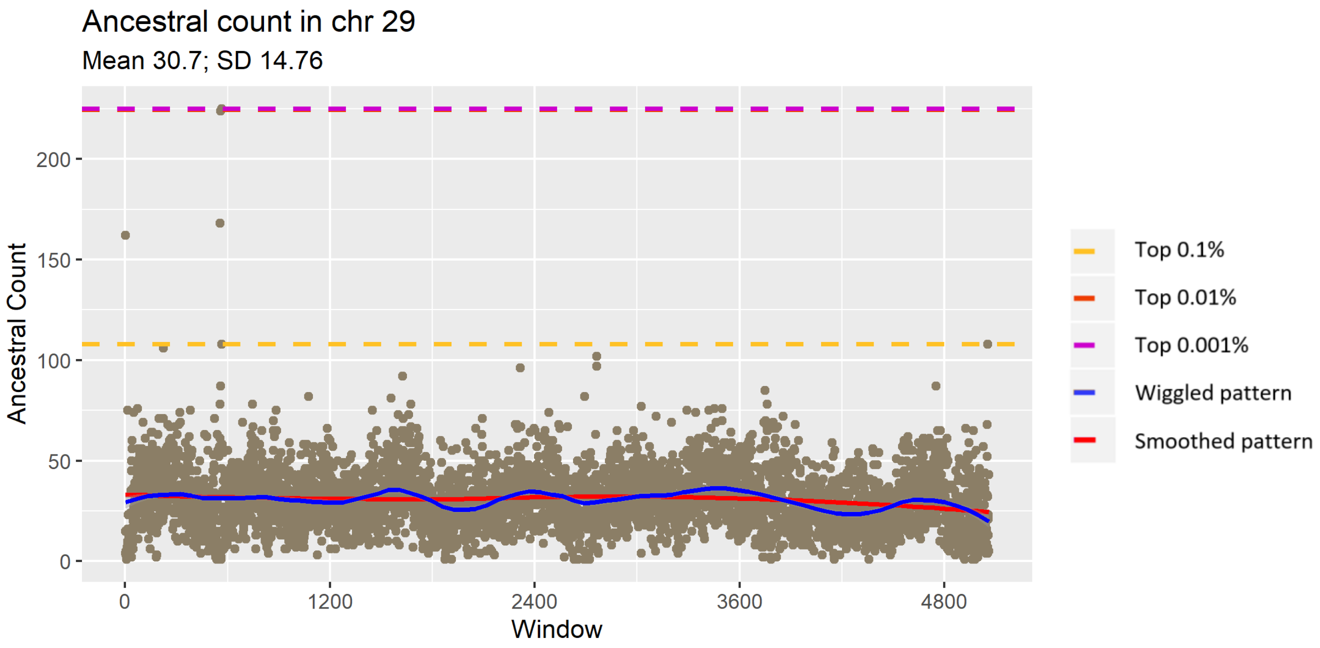
Ancestral count in chr 29
Mean 30.7; SD 14.76
Ancestral Count
Window
0
50
100
150
200
0
1200
2400
3600
4800
Top 0.1%
Top 0.01%
Top 0.001%
Wiggled pattern
Smoothed pattern
